# Optimizing rare disorder trials: a phase 1a/1b randomized study of KL1333 in adults with mitochondrial disease

**DOI:** 10.1093/brain/awae308

**Published:** 2024-12-09

**Authors:** Chiara Pizzamiglio, Renae J Stefanetti, Robert McFarland, Naomi Thomas, George Ransley, Matilda Hugerth, Alvar Grönberg, Sonia Simon Serrano, Eskil Elmér, Michael G Hanna, Magnus J Hansson, Gráinne S Gorman, Robert D S Pitceathly

**Affiliations:** Department of Neuromuscular Diseases, University College London Queen Square Institute of Neurology, London WC1N 3BG, UK; NHS Highly Specialised Service for Rare Mitochondrial Disorders, Queen Square Centre for Neuromuscular Diseases, The National Hospital for Neurology and Neurosurgery, London WC1N 3BG, UK; Wellcome Centre for Mitochondrial Research, Faculty of Medical Sciences, Newcastle University, Newcastle upon Tyne NE2 4HH, UK; Translational and Clinical Research Institute, Faculty of Medical Sciences, Newcastle University, Newcastle upon Tyne NE2 4HH, UK; NIHR Newcastle BRC, NHS Highly Specialised Service for Rare Mitochondrial Disorders, Newcastle upon Tyne Hospitals NHS Foundation Trust, Newcastle upon Tyne NE1 4LP, UK; Wellcome Centre for Mitochondrial Research, Faculty of Medical Sciences, Newcastle University, Newcastle upon Tyne NE2 4HH, UK; Translational and Clinical Research Institute, Faculty of Medical Sciences, Newcastle University, Newcastle upon Tyne NE2 4HH, UK; NIHR Newcastle BRC, NHS Highly Specialised Service for Rare Mitochondrial Disorders, Newcastle upon Tyne Hospitals NHS Foundation Trust, Newcastle upon Tyne NE1 4LP, UK; Wellcome Centre for Mitochondrial Research, Faculty of Medical Sciences, Newcastle University, Newcastle upon Tyne NE2 4HH, UK; Translational and Clinical Research Institute, Faculty of Medical Sciences, Newcastle University, Newcastle upon Tyne NE2 4HH, UK; NIHR Newcastle BRC, NHS Highly Specialised Service for Rare Mitochondrial Disorders, Newcastle upon Tyne Hospitals NHS Foundation Trust, Newcastle upon Tyne NE1 4LP, UK; Leonard Wolfson Experimental Neurology Centre, University College London Queen Square Institute of Neurology and The National Hospital for Neurology and Neurosurgery, London WC1N 3BG, UK; Abliva AB, SE-223 81 Lund, Sweden; Abliva AB, SE-223 81 Lund, Sweden; Abliva AB, SE-223 81 Lund, Sweden; Mitochondrial Medicine, Department of Clinical Sciences, Lund University, SE-221 84 Lund, Sweden; Abliva AB, SE-223 81 Lund, Sweden; Mitochondrial Medicine, Department of Clinical Sciences, Lund University, SE-221 84 Lund, Sweden; Department of Neuromuscular Diseases, University College London Queen Square Institute of Neurology, London WC1N 3BG, UK; NHS Highly Specialised Service for Rare Mitochondrial Disorders, Queen Square Centre for Neuromuscular Diseases, The National Hospital for Neurology and Neurosurgery, London WC1N 3BG, UK; Abliva AB, SE-223 81 Lund, Sweden; Mitochondrial Medicine, Department of Clinical Sciences, Lund University, SE-221 84 Lund, Sweden; Wellcome Centre for Mitochondrial Research, Faculty of Medical Sciences, Newcastle University, Newcastle upon Tyne NE2 4HH, UK; Translational and Clinical Research Institute, Faculty of Medical Sciences, Newcastle University, Newcastle upon Tyne NE2 4HH, UK; NIHR Newcastle BRC, NHS Highly Specialised Service for Rare Mitochondrial Disorders, Newcastle upon Tyne Hospitals NHS Foundation Trust, Newcastle upon Tyne NE1 4LP, UK; Department of Neuromuscular Diseases, University College London Queen Square Institute of Neurology, London WC1N 3BG, UK; NHS Highly Specialised Service for Rare Mitochondrial Disorders, Queen Square Centre for Neuromuscular Diseases, The National Hospital for Neurology and Neurosurgery, London WC1N 3BG, UK

**Keywords:** primary mitochondrial disease, rare diseases, clinical trial, phase 1 trial, KL1333

## Abstract

Over the past two decades there has been increased interest in orphan drug development for rare diseases. However, hurdles to clinical trial design for these disorders remain. This phase 1a/1b study addressed several challenges, while evaluating the safety and tolerability of the novel oral molecule KL1333 in healthy volunteers and subjects with primary mitochondrial disease.

KL1333 aims to normalize the NAD^+^:NADH ratio that is critical for ATP production. The trial incorporated innovative design elements with potential translatability to other rare diseases including patient involvement, adaptive design and exploratory objectives, all of which have subsequently informed the protocol of an ongoing phase 2, pivotal efficacy study of KL1333.

Results indicate KL1333 is safe and well tolerated, with dose-dependent gastrointestinal side effects, and validate potential novel outcome measures in primary mitochondrial disease including the 30-s Sit to Stand, and the patient-reported fatigue scales. Importantly, the data from the trial support efficacy of KL1333 based on improvements in fatigue and functional strength and endurance. Furthermore, the study highlights the value in using phase 1 studies to capture data that helps optimize later phase efficacy trial design.

## Teaser

Pizzamiglio *et al*. present the results of a phase 1a/b study evaluating the safety of KL1333, a new oral drug for primary mitochondrial disease that aims to normalize the NAD+:NADH ratio critical for ATP production. The trial, which incorporated patient input and adaptive design, showed KL1333 to be safe and well tolerated.

## Introduction

Rare diseases affect ∼300 million people worldwide, but only 5% have approved treatments.^1^ In the past two decades, there has been increased commercial interest in developing drugs for rare diseases due to advancements in genetic understanding and legislation offering financial incentives.^2^ However, challenges to drug development across rare diseases remain, including: small sample size, further restricted by rigid eligibility criteria; incomplete understanding of natural history; and a lack of sensitive outcome measures or validated biomarkers of treatment response.^3^

Primary mitochondrial diseases (PMDs) are the most common inherited neurometabolic disorders. They are caused by impaired oxidative phosphorylation (OxPhos), which limits ATP production.^4^ Symptoms affect various organs, and there are no approved therapies, with treatment being primarily supportive.^5,6^ In contrast to progress in diagnostics, drug development for PMDs lags due to the complexity of mitochondrial genetics, the diverse catalogue of causal genes, and the lack of reliable outcome measures. These obstacles compound the inherent broader challenges to trial design in rare diseases and underscore the urgent need for innovative therapeutic approaches.^7^

Here, we report results of a phase 1a/1b study evaluating KL1333’s safety and tolerability in healthy volunteers and PMD subjects. KL1333 [2-isopropyl-3H-naphtho(2,1-d)imidazole-4,5-dione], an oral small molecule specifically developed for PMDs, has been shown to normalize the NAD^+^:NADH ratio in PMD fibroblasts.^8^ This ratio is critical for ATP production and its restoration is linked to improved OxPhos. NAD metabolism is essential, and NAD^+^ depletion has been shown to contribute to several pathological processes including neurodegeneration, ageing and tumorigenesis.^9^ A previous first-in-human study established the safety and tolerability of KL1333.^10^

Our manuscript focuses on innovative trial design, emphasizing early involvement and recruitment of PMD subjects, novel methodologies and exploratory objectives aimed at informing a later phase efficacy study. It offers insights into strategies addressing clinical trial challenges in rare diseases and highlights the significance of early-phase trials, not only to confirm safety but enable identification of appropriate end points.

## Materials and methods

### Study design

**Figure 1:**
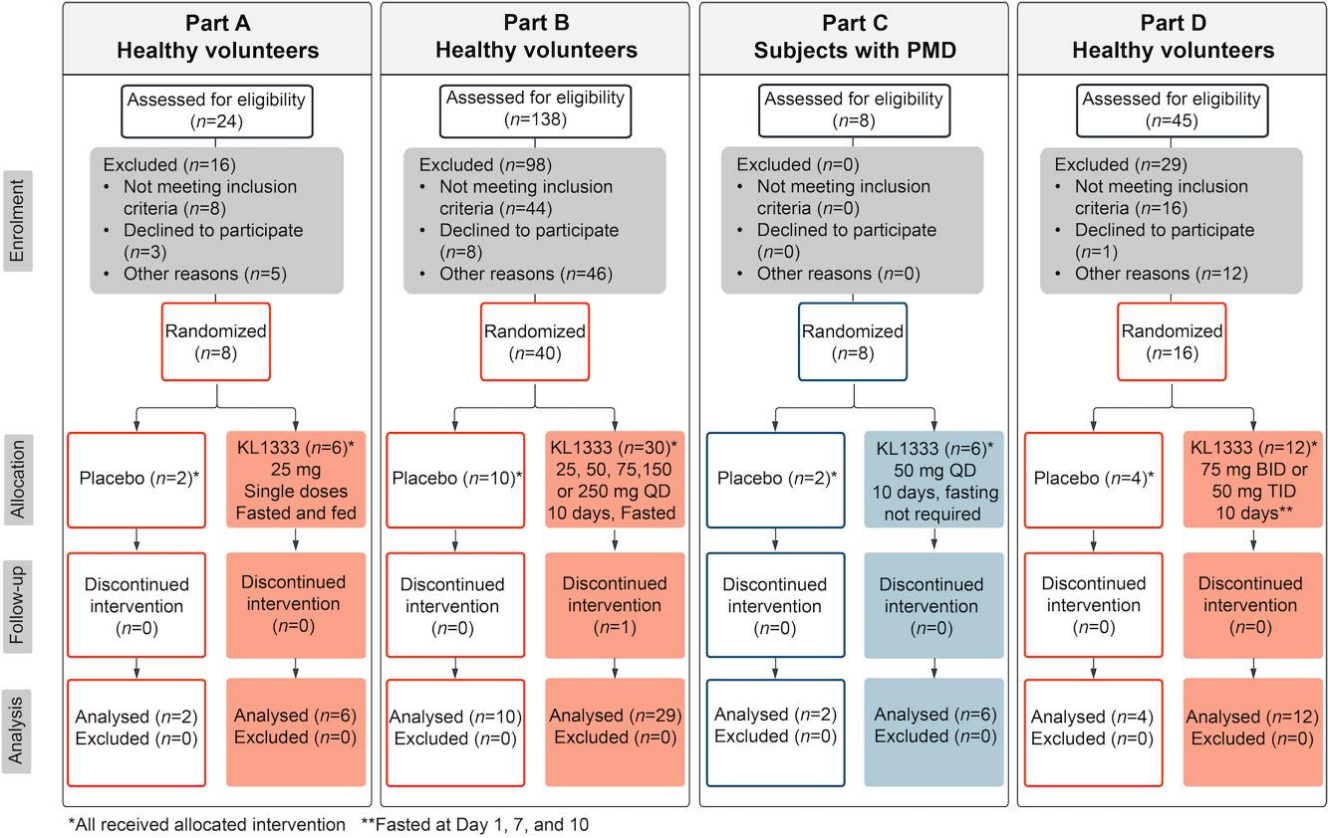
**Participant flow diagram.** PMD = primary mitochondrial disease; QD = once daily; BID = twice daily; TID = three times daily.

This double-blind, randomized, placebo-controlled, single and multiple oral dose phase 1a/1b study was conducted in four parts (A, B, C and D). Parts A, B and D included a total of eight cohorts of healthy volunteers, while Part C, conducted at completion of Parts A and B, included one cohort of subjects with genetically confirmed PMDs. The primary objective of the study was to evaluate the safety and tolerability of KL1333 in healthy subjects and people with PMD. Other objectives were to explore pharmacokinetics (PK), food effect and pharmacodynamics (PD) of KL1333. An overview of the study design is shown in Fig. 1, Supplementary Table 1 and the Supplementary material. The investigational medicinal products were provided as 25 and 100 mg KL1333 encapsulated tablets and matching placebo tablets.

The two healthy cohorts in Part D, receiving the same daily dose of 150 mg divided in two or three doses, were added later to refine the tolerability profile of KL1333, after reviewing preliminary results from Parts B and C.

### Standard protocol approvals, registrations and patient consents

The study (ClinicalTrials.gov: NCT03888716, EudraCT: 2018-001794-24) was conducted in the UK, at Labcorp Clinical Research Unit, Leeds (Parts A, B and D of study) and at two national specialist PMD centres in Newcastle upon Tyne and London (Part C). The study was run in accordance with the Declaration of Helsinki and the International Conference on Harmonization Good Clinical Practice guidelines following regulatory and ethical approval from the Medicines and Healthcare products Regulatory Agency and the South-Central Berkshire B Research Ethics Committee (Ref: 18/SC/0286). Written informed consent was obtained from all participants.

### Study participants

Parts A, B and D included healthy volunteers aged 18–65 years, body weight ≥50 kg and body mass index (BMI) 18.0–32.0 kg/m^2^. Part C included subjects aged 18–75 years with any genetically confirmed PMD who were clinically stable and had a BMI of 15.0–32.0 kg/m^2^. A full list of inclusion and exclusion criteria is in the Supplementary material, ‘Protocol’ section.

### Study assessments

Participants were monitored for adverse events (AEs). Other safety assessments included vital signs, clinical laboratory tests in blood and urine, and 12-lead ECGs.

Blood samples to assess plasma KL1333 concentrations and pharmacokinetic parameters were obtained. Pharmacodynamic biomarkers were obtained pre-dose in Parts B to D and at 0.5 and 2-h post-dose in Part D. These included lactate and pyruvate concentrations and ratio in whole blood, FGF21 and GDF15 concentrations in serum, total NAD^+^ and NADH concentrations and ratio in whole blood (Supplementary material).

Exploratory clinical outcome assessments in PMD (Part C) included the 30-s Sit to Stand (30 s STS) test, to assess functional lower body muscle strength and endurance, and two patient-rated scales evaluating fatigue.^11,12^ The Daily Fatigue Impact Scale (DFIS)^13^ was completed daily from Day −1 to Day 11, and the Neuro-QoL Short Form Fatigue^14^ was completed at Day −1 and Day 10. For both scales, higher scores indicate greater fatigue. Patient status was assessed by using the Newcastle Mitochondrial Disease Adult Scale (NMDAS),^15^ the Patient Global Impression (PGI) and Clinician Global Impression (CGI) of disease severity (Supplementary material).

Importantly, patients with PMDs were involved in the selection of clinically meaningful outcome measures through externally led patient-focused drug development meetings.

### Statistical analysis

No sample size calculation was conducted, as is standard for phase 1 studies.

Adverse events were summarized by treatment, severity and relationship to study drug. An adverse event was treatment-related if it was assessed by the investigator as related or possibly related to the study treatment.

For pharmacodynamic assessments, summary statistics were calculated for observed values and change from baseline. Changes from baseline for biomarkers were estimated from a longitudinal repeated measures mixed effects model (Supplementary material).

Data analysis was performed using SAS® version 9.4 (SAS Institute, Cary, NC, USA). Exploratory analyses of biomarkers and correlations were performed using GraphPad Prism version 9.3.1 or higher (GraphPad Software, San Diego, CA, USA).

## Results

### Study population

Across all study parts, 72 participants were randomized and dosed. Of these, eight had PMD (Fig. 1). Fifty-four participants received KL1333 (six with PMD) and 18 received the matched placebo (two with PMD).

Baseline characteristics were similar between cohorts, except mean body weight and BMI, which were notably lower in subjects with PMDs compared to healthy volunteers (Table 1). All PMD subjects had genetically confirmed disease caused by mtDNA point mutations (the most common cause of PMD in adults), six with tRNA and two with complex I mutations. The baseline NMDAS section I–III total score ranged 8–48 (Table 1 and Supplementary Fig. 1). Exercise intolerance and/or proximal myopathy was a common feature, being present in seven subjects (87.5%) (Supplementary material and Supplementary Fig. 1).

**Table 1:** Summary of demographics and baseline characteristics

	Part A Healthy subjects (*n* = 8)	Part B Healthy subjects (*n* = 40)	Part C PMD (*n* = 8)	Part D Healthy subjects (*n* = 16)
Age (years), mean (SD)	52.6 (9.24)	41.6 (14.70)	45.6 (15.63)	40.1 (14.25)
Sex, *n* (%)				
Male	4 (50.0%)	27 (67.5%)	4 (50.0%)	11 (68.8%)
Female	4 (50.0%)	13 (32.5%)	4 (50.0%)	5 (31.3%)
Race, *n* (%)				
White	8 (100.0%)	38 (95.0%)	8 (100.0%)	15 (93.8%)
Multiple	–	1 (2.5%)	–	–
Black or African American	–	1 (2.5%)	–	1 (6.3%)
Ethnicity, *n* (%)				
Not Hispanic or Latino	8 (100%)	40 (100%)	8 (100%)	16 (100.0%)
Height (cm), mean (SD)	169.0 (10.11)	172.5 (8.63)	170.53 (9.71)	172.44 (8.92)
Weight (kg), mean (SD)	77.55 (8.26)	76.23 (11.63)	69.09 (16.43)	73.94 (12.57)
BMI (kg/m^2^), mean (SD)	27.16 (1.78)	25.57 (2.92)	23.56 (4.28)	24.83 (3.58)
**Genotype, *n* (%)**				
mtDNA-encoded respiratory chain proteins				
m.3761C>A (*MT-ND5*)	–	–	1 (12.5%)	–
m.13513G>A (*MT-ND1*)	–	–	1 (12.5%)	–
mtDNA-encoded tRNA				
m.8344A>G (*MT-TK*)	–	–	4 (50.0%)	–
m.3243A>G (*MT-TL1*)	–	–	1 (12.5%)	–
m.14709T>C (tRNA Glu)	–	–	1 (12.5%)	–
NMDAS, mean (range)				
Total score	–	–	22.4 (8–48)	–
Current function (I)	–	–	10.1 (2–25)	–
System specific involvement (II)	–	–	5.88 (3–8)	–
Current clinical assessment (III)	–	–	6.38 (1–16)	–

BMI = body mass index; mtDNA = mitochondrial DNA; PMD = primary mitochondrial disease; NMDAS = Newcastle Mitochondrial Disease Adult Scale; SD = standard deviation.

### Safety and tolerability

There were no clinically significant findings for laboratory evaluations, vital signs, 12-lead ECGs or physical examinations. Overall, KL1333 was well tolerated when administered up to 75 mg once daily (QD) for 10 days in healthy participants, and when administered as 50 mg QD to subjects with PMDs. There were no serious adverse events (SAEs) or participant discontinuation due to adverse events (Supplementary Table 2), although one participant in Part B withdrew consent on Day 5 after receiving four doses of 150 mg KL1333 QD.

Gastrointestinal disorders were the most common dose-related side effects. In healthy volunteers, 250 mg KL1333 QD (Part B) was poorly tolerated due to abdominal pain and diarrhoea. These side effects improved by dividing a daily 150 mg dose into two 75 mg doses or three 50 mg doses (Supplementary Table 3), as shown in Part D. A dose of 50 mg three times a day (TID) had an adverse event profile that was similar to the corresponding placebo. In PMD subjects, mild gastrointestinal side effects were reported in four (75%) KL1333-treated subjects, one episode of abdominal bloating, one of diarrhoea, and two episodes of nausea.

### Biomarkers

**Figure 2:**
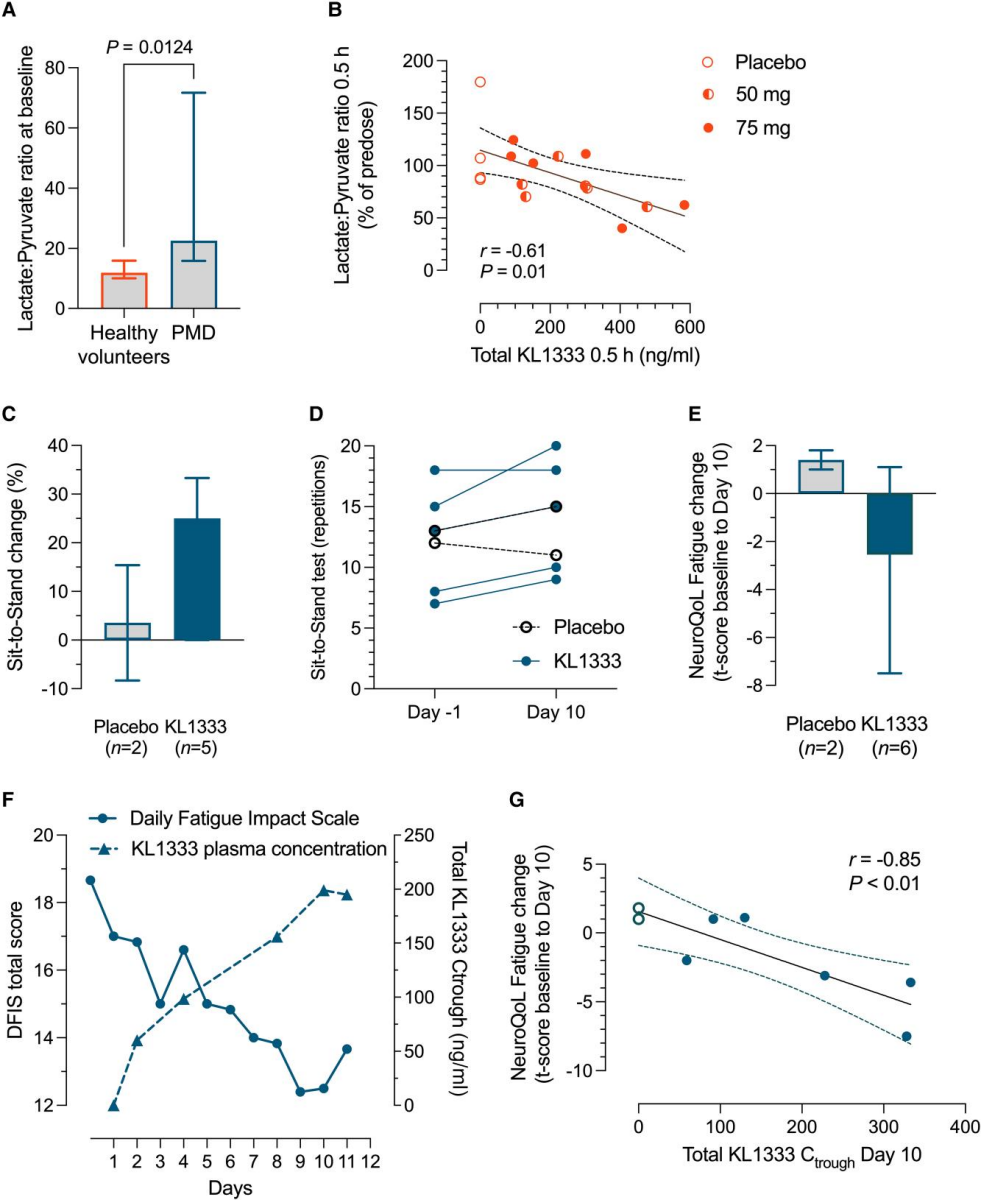
**Biomarkers and clinical outcome assessments.** (**A**) Lactate:pyruvate ratio analysed at baseline before treatment initiation in healthy volunteers (Parts B and D combined, *n* = 56) and subjects with PMD (Part C, *n* = 8). Group differences evaluated with Mann-Whitney test. (**B**) Correlation between lactate:pyruvate ratio changes from baseline to 0.5 h and total plasma KL1333 concentration following treatment initiation Day 1 in healthy volunteers (Part D, *n* = 16). (**C**) Group percentage changes in number of Sit to Stand (STS) repetitions performed during 30 s from baseline (Day −1) to Day 10 following daily oral doses of 50 mg KL1333 (*n* = 5, one individual could not perform test due to severe myopathy) or placebo (*n* = 2) and (**D**) changes in individual number of STS repetitions. (**E**) Group NeuroQoL Fatigue t-scores changes in patients treated with KL1333 (*n* = 6) or placebo (*n* = 2). (**F**) Time profile of Daily Fatigue Impact Scale (DFIS) mean scores and arithmetic mean total KL1333 pre-dose/Ctrough concentrations in KL1333-treated subjects with PMD (*n* = 6). (**G**) Changes in individual NeuroQoL Fatigue t-scores from baseline to follow-up at Day 10 plotted against total KL1333 Ctrough values at Day 10. All bar graphs present medians ± 95% confidence interval (CI). Ctrough = KL1333 concentration immediately before the next dose.

As expected, based on previously published studies,^16^ baseline blood lactate, lactate:pyruvate ratio, serum GDF15 and FGF21 were significantly elevated in PMD subjects compared to healthy volunteers (Fig. 2A and Supplementary Table 4). Total NAD^+^ and NADH content in whole blood did not differ between patients and healthy volunteers, and these biomarkers were characterized by large day-to-day variations (Supplementary Table 4). FGF21 and GDF15 were relatively stable over time and did not show any change with treatment (Supplementary Table 5). Lactate and pyruvate levels were also characterized by substantial day-to-day variation. However, in one of the healthy volunteer cohorts (Part D), blood samples were drawn early after treatment initiation and a negative correlation (*r* = −0.61) between lactate:pyruvate ratio changes from baseline to 0.5 h post-treatment and total KL1333 plasma concentration at the same time point was seen (Fig. 2B).

### Clinical outcome assessments

Subjects with PMDs identified fatigue and muscle symptoms as their main unmet needs.^17,18^ Therefore, clinical outcome assessments were selected to align with these concerns, making them suitable for evaluation of change induced by KL1333, which regulates ATP levels.

In the PMD cohort, there were notable differences between the KL1333-treated and the placebo group with regards to fatigue patient-reported measurements and 30 s STS test (Supplementary Table 6).

With regards to the 30 s STS test, the mean improvement was 2.2 repetitions (20.5%) for the five participants in the KL1333 group (one participant with severe myopathy was unable to perform the test), versus 0.5 repetitions (3.6%) in the two placebo-treated participants (Fig. 2C and Supplementary Table 6). The KL1333 group included one patient without myopathy who performed 18 repetitions at baseline and follow-up; the other four participants improved between 2–5 repetitions following 10 days of treatment (Fig. 2D).

Treated subjects demonstrated an improvement in both Neuro-QoL Short Form Fatigue and DFIS. The Neuro-QoL Short Form Fatigue showed a mean change in standardized total t-score of −2.4, indicating less fatigue, compared to the placebo-treated PMD subjects that showed a mean change of total t-score of +1.4, indicating fatigue worsening (Fig. 2E). For the DFIS, there was a gradual day-by-day reduction of fatigue in treated participants, with the mean total severity score decreasing from 18.7 on Day −1 to a nadir of 12.4 at Day 9 and 12.5 at Day 10 (Fig. 2F). For both scales, there were statistically significant correlations between KL1333 plasma concentrations at steady state and the magnitude of change in fatigue (Pearson coefficients >0.8) (Fig. 2G and Supplementary Fig. 2). There were no changes in PGI and CGI scales (Supplementary Table 6).

## Discussion

The multi-step process of drug development is particularly challenging for rare diseases, with unique hurdles that must be effectively addressed to prevent inefficient data collection and expedite therapy approval.^2^

Phase 1 trials mark the earliest human exposure to a new drug, with data collection being often limited to short-term toxicity and pharmacological data in healthy subjects.^19^ This article highlights the potential of such early phase studies to extend beyond their primary end points. Here, we have established KL1333, a NAD^+^/NADH modulator, as safe and well tolerated. We have also provided insights into potential outcome measures relevant for clinical efficacy trials of PMDs. Given the urgent need for new therapies for this population, the study adopted a hybrid design that enabled concomitant enrolment of healthy and PMD subjects to facilitate early KL1333 exposure to people with PMDs, while informing the design of later stage trials.

No safety concerns were identified. There was a dose-dependent increase in gastrointestinal side effects in healthy volunteers, and KL1333 was best tolerated when the daily dose was divided, indicating a gastrointestinal irritant effect at higher doses. An adaptive design was employed, allowing modifications based on data accumulation.^20^ This enabled addition of a fourth cohort (Part D) of healthy volunteers to refine KL1333’s tolerability profile, addressing the observed gastrointestinal side effects. This dosing regimen has now been incorporated in the ongoing phase 2 trial. Similarly, Part B adopted a flexible design, with a dose selection conference meeting reviewing blinded data prior to initiating a higher dose.

The ‘necessity’ of developing patient-centred outcome measures for rare diseases has been included in the 2016 statement from the Task Force on Patient-Centred Outcome Measures of the International Rare Diseases Research Consortium.^21^ For this reason, externally-led PMD patient-focused drug development meetings were conducted; when ranking unmet needs, a reduction in chronic fatigue and muscle weakness were scored highest by patients and were the lead symptoms that attracted patient study participation.^17,18^ Indeed, the main clinical outcome assessments—30 s STS and patient-reported fatigue scales—were selected based on both the relevance in PMD patients and the assumption that these disease concepts would be amenable to change by a compound, KL1333, that modulates energy metabolism and has been designed to treat symptoms, such as chronic fatigue and myopathy in adults with PMDs. The involvement of patients in the selection of clinical outcome assessments is a key aspect of our study, reflecting the importance of patient-centred approaches in clinical research. While including patient perspectives in phase 1 is an innovative step, we acknowledge the necessity of conducting dedicated natural history studies to ensure the success of clinical trials. This is particularly important for identifying the most sensitive and reliable measures to capture clinical changes and detecting meaningful clinical changes.

Changes in the 30 s STS test performance suggest its potential as a sensitive measure of functional capacity in PMDs. Patient performance was below normative values, and KL1333-treated participants showed a mean improvement of 2.2 repetitions, compared to 0.5 in the placebo group. Although the clinical impact of this change has not previously been evaluated, a two-repetition gain is considered clinically meaningful in other disorders.^22,23^ Fatigue, a disabling and common symptom in PMDs, decreased with KL1333 treatment, indicating an efficacy signal.

Biomarkers in rare diseases are critical, and when a strong link to the disease pathogenesis is established, they could facilitate regulatory approval.^2^ In PMDs, studies have mainly focused on diagnostic biomarkers and there are no established pharmacodynamic biomarkers.^24,25^ Despite the paucity of data on response to drug treatments, several of the investigated biomarkers have a link to NAD^+^:NADH reductive stress.^26,27^ While, as expected, blood lactate, lactate:pyruvate ratio, GDF15 and FGF21 were elevated at basal levels in subjects with PMDs compared to healthy volunteers, their role as treatment response biomarkers is uncertain. FGF21 and GDF15, despite being linked with reductive stress, did not significantly change post-dose. While blood lactate is a marker of mitochondrial dysfunction and reductive stress, it is highly non-specific and variable.^16,28^ Consequently, the significant correlation between KL1333 exposure and decreases in lactate:pyruvate ratio seen in the present study may signal a pharmacodynamic effect, but should be interpreted with caution. Of note, lactate and pyruvate changes are not necessarily expected to be mirrored by NAD^+^:NADH changes with the methodology used in the present study. First, lactate:pyruvate ratio is considered related to free intracellular NAD^+^/NADH redox status rather than total content.^29^ Second, lactate and pyruvate levels measured in blood do not primarily reflect redox status only in blood, whereas NAD(H) measured content in blood. A further limitation to interpreting NAD(H) in blood is that blood heteroplasmy levels in adults with mtDNA associated disease are typically low and below what would be expected to cause any biochemical perturbations.^16^ Taken together, the extreme variability observed in lactate, pyruvate and total NAD^+^/NADH levels in whole blood limits their reliability as biomarkers for treatment response in mitochondrial myopathies. As an alternative, FGF21 and GDF15 have emerged as promising biomarkers for disease severity and potentially for treatment response in selected mitochondrial myopathy genotypes.^30^ This is particularly relevant given the current paucity of effective treatments for mitochondrial myopathies, which has resulted in limited data on the utility of these biomarkers in clinical settings. Overall, additional studies are warranted to establish more precise biomarkers with pharmacodynamic properties in PMD.

Importantly, the findings from this phase 1a/1b trial have significantly influenced the design of the ongoing phase 2 efficacy trial (ClinicalTrials.gov: NCT05650229). This phase 1 study found that the patient-reported fatigue scales and 30 s STS were sensitive outcome measures for PMDs and thus, these have become pivotal in phase 2, guiding the selection of primary end points and inclusion criteria. Furthermore, including a genetically diverse mtDNA population informed the decision to encompass patients with mtDNA-related diseases, with the presence of fatigue and myopathic phenotype being a prerequisite for inclusion, and exclusion of neurodegenerative phenotypes.

Study limitations include the short treatment period and low number of PMD subjects with various genetic backgrounds and symptoms, with no specific phenotypic requirements in the inclusion criteria. This is in line with the phase 1 study setting and objectives to evaluate safety, tolerability and pharmacokinetics in healthy volunteers as well as in a general PMD population. The biomarkers and clinical outcome assessments were hence not powered to evaluate group differences, and the presented *post hoc* analyses were exploratory. Regarding biomarkers, while our study did not identify treatment response biomarkers for PMD, it highlighted the limitations of certain biochemical parameters and will serve as a foundation for future larger studies. This is crucial given the limited data and treatments for PMDs, which has resulted in limited utility of these biomarkers in clinical settings so far. Nevertheless, these findings support patient-reported fatigue and lower extremity functional assessments when evaluating therapy efficacy in PMD.

**Figure 3:**
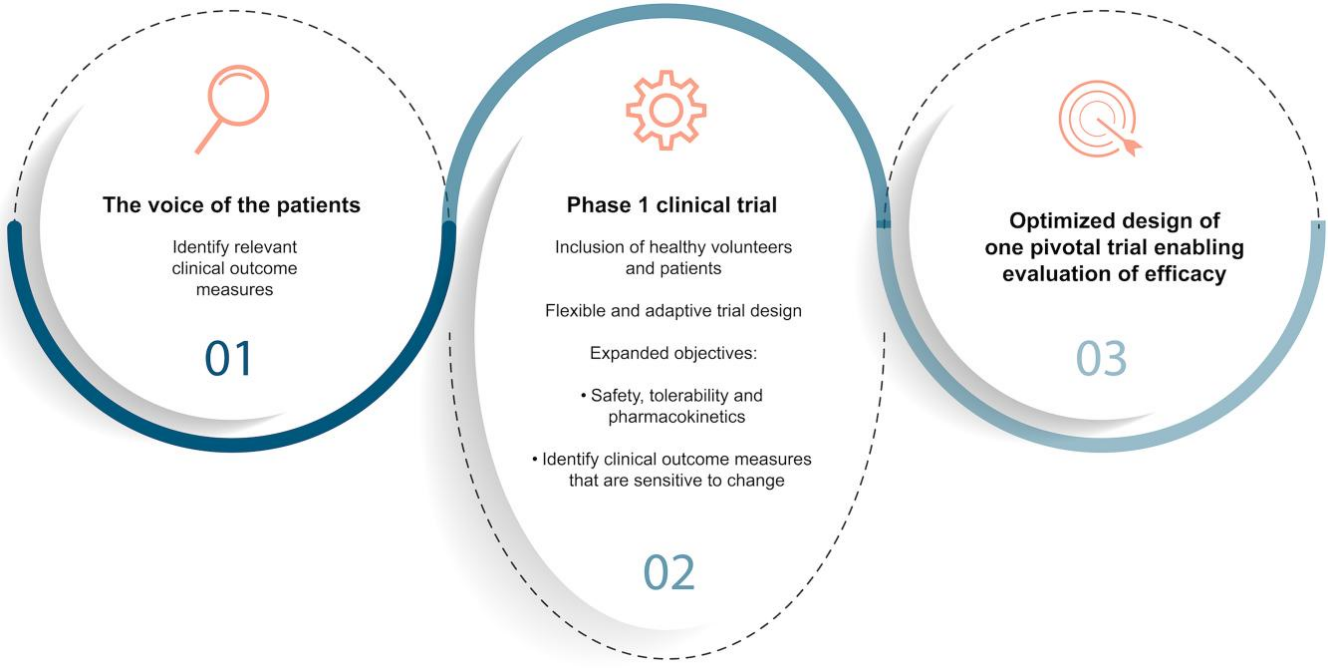
**Optimizing clinical trial design in rare disease.** Phase 1 trials have the opportunity to play a central role in the therapeutic development for rare diseases, where patient recruitment possibilities are limited.

In conclusion, KL1333 was deemed safe and well tolerated in healthy volunteers and PMDs. Furthermore, the innovative nature of this phase 1a/1b clinical trial offers insights relevant to other rare disorders (Fig. 3). Learnings include patient input on clinically meaningful outcome measures; the concurrent randomization of healthy and PMD subjects; an adaptive trial design with an additional cohort (Part D) to refine KL1333’s tolerability; and collection of biomarkers and outcome measures guiding phase 2 design. These data contribute to shaping the ongoing phase 2 trial of KL1333, underscoring the value of optimizing phase 1 trials beyond safety data acquisition.

## Supplementary Material

awae308_Supplementary_Data

## Data Availability

The data supporting findings of this study are available from the corresponding author, upon reasonable request.

## References

[1] Nguengang Wakap S , Lambert DM, Olry A, et al Estimating cumulative point prevalence of rare diseases: Analysis of the orphanet database. Eur J Hum Genet. 2020;28:165–173. 31527858 10.1038/s41431-019-0508-0 PMC6974615

[2] Pizzamiglio C , Vernon HJ, Hanna MG, Pitceathly RDS. Designing clinical trials for rare diseases: Unique challenges and opportunities. Nat Rev Methods Primers. 2022;2:s43586-022-00100-2. 36254116 10.1038/s43586-022-00100-2 PMC7613711

[3] Winter E , Schliebner S. Current advances in clinical trials for rare disease populations: Spotlight on the patient. Curr Rev Clin Exp Pharmacol. 2022;17:39–45. 33726656 10.2174/1574884716666210316120615

[4] Ng YS , Bindoff LA, Gorman GS, et al Mitochondrial disease in adults: Recent advances and future promise. Lancet Neurol. 2021;20:573–584. 34146515 10.1016/S1474-4422(21)00098-3

[5] Pitceathly RDS , Keshavan N, Rahman J, Rahman S. Moving towards clinical trials for mitochondrial diseases. J Inherit Metab Dis. 2021;44:22–41. 32618366 10.1002/jimd.12281 PMC8432143

[6] Russell OM , Gorman GS, Lightowlers RN, Turnbull DM. Mitochondrial diseases: Hope for the future. Cell. 2020;181:168–188. 32220313 10.1016/j.cell.2020.02.051

[7] Falabella M , Minczuk M, Hanna MG, Viscomi C, Pitceathly RDS. Gene therapy for primary mitochondrial diseases: Experimental advances and clinical challenges. Nat Rev Neurol. 2022;18:689–698. 36257993 10.1038/s41582-022-00715-9

[8] Seo KS , Kim JH, Min KN, et al KL1333, a novel NAD(+) modulator, improves energy metabolism and mitochondrial dysfunction in MELAS fibroblasts. Front Neurol. 2018;9:552. 30026729 10.3389/fneur.2018.00552 PMC6041391

[9] Zapata-Perez R , Wanders RJA, van Karnebeek CDM, Houtkooper RH. NAD(+) homeostasis in human health and disease. EMBO Mol Med. 2021;13:e13943. 34041853 10.15252/emmm.202113943 PMC8261484

[10] Yang E , Yoo H, Ban M, et al Pharmacokinetics and safety/tolerability of Kl1333, a novel agent for primary mitochondrial disorders in healthy male subjects. Clinical Pharmacol Ther. 2019;105:S20.

[11] Jones CJ , Rikli RE, Beam WC. A 30-s chair-stand test as a measure of lower body strength in community-residing older adults. Res Q Exerc Sport. 1999;70:113–119. 10380242 10.1080/02701367.1999.10608028

[12] Stefanetti RJ , Blain A, Jimenez-Moreno C, et al Measuring the effects of exercise in neuromuscular disorders: A systematic review and meta-analyses. Wellcome Open Res. 2020;5:84. 32671231 10.12688/wellcomeopenres.15825.1 PMC7331112

[13] Fisk JD , Doble SE. Construction and validation of a fatigue impact scale for daily administration (D-FIS). Qual Life Res. 2002;11:263–272. 12074263 10.1023/a:1015295106602

[14] Cella D , Lai JS, Nowinski CJ, et al Neuro-QOL: Brief measures of health-related quality of life for clinical research in neurology. Neurology. 2012;78:1860–1867. 22573626 10.1212/WNL.0b013e318258f744 PMC3369516

[15] Schaefer AM , Phoenix C, Elson JL, McFarland R, Chinnery PF, Turnbull DM. Mitochondrial disease in adults: A scale to monitor progression and treatment. Neurology. 2006;66:1932–1934. 16801664 10.1212/01.wnl.0000219759.72195.41

[16] Parikh S , Goldstein A, Koenig MK, et al Diagnosis and management of mitochondrial disease: A consensus statement from the mitochondrial medicine society. Genet Med. 2015;17:689–701. 25503498 10.1038/gim.2014.177 PMC5000852

[17] United Mitochondrial Disease Foundation . Voice of the Patient Report “Mitochondrial Disease: Adults with Myopathy, Children with Neurologic Symptoms”. Published 3 December 2019. Accessed 12 November 2024. https://www.umdf.org/wp-content/uploads/2020/11/UMDF-EL-PFDD-Meeting-VOP-Report_v2019-12-03-JRG.pdf

[18] Zolkipli-Cunningham Z , Xiao R, Stoddart A, et al Mitochondrial disease patient motivations and barriers to participate in clinical trials. PLoS One. 2018;13:e0197513. 29771953 10.1371/journal.pone.0197513 PMC5957366

[19] Sramek JJ , Murphy MF, Adcock S, Stark JG, Cutler NR. Phase 1 clinical trials of small molecules: Evolution and state of the art. Rev Recent Clin Trials. 2021;16:232–241. 33563172 10.2174/1574887116666210204125844

[20] Bhatt DL , Mehta C. Adaptive designs for clinical trials. N Engl J Med. 2016;375:65–74. 27406349 10.1056/NEJMra1510061

[21] Lochmuller H , Torrent IFJ, Le Cam Y, et al The international rare diseases research consortium: Policies and guidelines to maximize impact. Eur J Hum Genet. 2017;25:1293–1302. 29158551 10.1038/s41431-017-0008-z PMC5865169

[22] Zanini A , Crisafulli E, D'Andria M, et al Minimum clinically important difference in 30-s sit-to-stand test after pulmonary rehabilitation in subjects with COPD. Respir Care. 2019;64:1261–1269. 31270178 10.4187/respcare.06694

[23] Wright AA , Cook CE, Baxter GD, Dockerty JD, Abbott JH. A comparison of 3 methodological approaches to defining major clinically important improvement of 4 performance measures in patients with hip osteoarthritis. J Orthop Sports Phys Ther. 2011;41:319–327. 21335930 10.2519/jospt.2011.3515

[24] Lehtonen JM , Forsstrom S, Bottani E, et al FGF21 is a biomarker for mitochondrial translation and mtDNA maintenance disorders. Neurology. 2016;87:2290–2299. 27794108 10.1212/WNL.0000000000003374 PMC5270510

[25] Yatsuga S , Fujita Y, Ishii A, et al Growth differentiation factor 15 as a useful biomarker for mitochondrial disorders. Ann Neurol. 2015;78:814–823. 26463265 10.1002/ana.24506 PMC5057301

[26] Fujita Y , Ito M, Kojima T, Yatsuga S, Koga Y, Tanaka M. GDF15 is a novel biomarker to evaluate efficacy of pyruvate therapy for mitochondrial diseases. Mitochondrion. 2015;20:34–42. 25446397 10.1016/j.mito.2014.10.006

[27] Sharma R , Reinstadler B, Engelstad K, et al Circulating markers of NADH-reductive stress correlate with mitochondrial disease severity. J Clin Invest. 2021;131:e136055. 33463549 10.1172/JCI136055 PMC7810486

[28] Titov DV , Cracan V, Goodman RP, Peng J, Grabarek Z, Mootha VK. Complementation of mitochondrial electron transport chain by manipulation of the NAD+/NADH ratio. Science. 2016;352:231–235. 27124460 10.1126/science.aad4017 PMC4850741

[29] Williamson DH , Lund P, Krebs HA. The redox state of free nicotinamide-adenine dinucleotide in the cytoplasm and mitochondria of rat liver. Biochem J. 1967;103:514–527. 4291787 10.1042/bj1030514 PMC1270436

[30] Dominguez-Gonzalez C , Badosa C, Madruga-Garrido M, et al Growth differentiation factor 15 is a potential biomarker of therapeutic response for TK2 deficient myopathy. Sci Rep. 2020;10:10111. 32572108 10.1038/s41598-020-66940-8 PMC7308386

