# Supplementary material for: Optimizing rare disorder trials: a phase 1a/1b randomized study of KL1333 in adults with mitochondrial disease
Source: Brain. 2024 Dec 9;148(1):39–46. doi: 10.1093/brain/awae308 (PMC11706290; doi:10.1093/brain/awae308)
Supplement: awae308_Supplementary_Data [file awae308_supplementary_data.zip › Protocol.pdf]

Protocol

A Randomised, Double-blind, Parallel-group, Placebo-controlled, Phase Ia/Ib, Multiple-site Study to Assess the Safety, Tolerability, Pharmacokinetics, and Pharmacodynamics of KL1333 after a Single Oral Dose and Multiple Ascending Oral Doses in Healthy Subjects and Patients with Primary Mitochondrial Disease

Version History:
Protocol Version 1 Date: 29 August 2018
Protocol Version 2 Date: 25 September 2018, including Protocol Amendment 1
Protocol Version 3 Date: 25 January 2019, including Protocol Amendment 2
Protocol Version 4 Date: 15 May 2019, including Protocol Amendment 3
Protocol Version 5 Date: 19 August 2019, including Protocol Amendment 4
Protocol Version 6 Date: 31 October 2019, including Protocol Amendment 5
Protocol Version 7 Date: 23 October 2020, including Protocol Amendment 6
Protocol Version 8 Date: 04 February 2021, including Protocol Amendment 7

Investigational Product: KL1333

Protocol Reference Number: KL1333 2018-102
Covance Study Number: 8387160
EudraCT Number: 2018-001794-24

Sponsor:
Abliva AB
Medicon Village
223 81 Lund
Sweden

Study Site:
Multiple Sites

Sponsor Signatories:
Magnus Hansson, MD, PhD

Matilda Hugerth, MSc, Pharm

Principal Investigator:
Multiple Investigators

Information described herein is confidential and may be disclosed only with the express written permission of the Sponsor.

SPONSOR APPROVAL

I have read the protocol and approve it:

Magnus Hansson

Digitally signed by Magnus Hansson
DN: cn=Magnus Hansson, o=Abliva AB, ou, email=magnus.hansson@abliva.com, c=SE
Date: 2021.02.05 11:57:00 +01'00'

Magnus Hansson, MD, PhD<br>Chief Medical Officer, QPPV	Date

Matilda Hugerth

Elektroniskt undertecknad av Matilda Hugerth
SN: cn=Matilda Hugerth, o=Abliva AB, ou, email=matilda.hugerth@abliva.com, c=SE
Datum: 2021.02.05 11:38:20 +01'00'

Matilda Hugerth, MSc, Pharm<br>Director, Clinical and Regulatory Affairs	Date

INVESTIGATOR AGREEMENT

I have read the protocol and agree to conduct the study as described herein.

____

Investigator’s Name (Printed)

____________________________________ ________________________________

Investigator’s Signature Date

STUDY IDENTIFICATION

Sponsor	Abliva AB<br>Medicon Village<br>223 81 Lund, Sweden
Sponsor's Study Contact and Medical Contact	Magnus Hansson, MD, PhD<br>Chief Medical Officer, QPPV<br>Abliva AB<br>Medicon Village<br>223 81 Lund, Sweden
Sponsor's Study Contact	Matilda Hugerth, MSc, Pharm<br>Director, Clinical and Regulatory Affairs<br>Abliva AB<br>Medicon Village<br>223 81 Lund, Sweden
Covance Medical Monitor	Arpan Dutta, MBChB(Hons), LLM, MD, PGDip, MRCPsych, FHEA<br>Associate Medical Director<br>Covance Inc.<br>Springfield House<br>Hyde Street<br>Leeds, LS2 9LH, UK<br>Tel: +44 (0)786 613 6191
Study Site	Multiple Sites
Principal Investigator	Multiple Investigators
Bioanalytical Laboratory	Covance Clinical Pathology Services<br>Otley Road<br>Harrogate, HG3 1PY, UK
Faecal Calprotectin Laboratory	Covance Central Laboratory Services<br>Rue Moïse-Marcinhes 7<br>1217 Meyrin, Switzerland
Biomarker and Genotyping Laboratory	Complement Genomics Ltd.<br>The Durham Genome Centre<br>Park House<br>Station Road<br>Lanchester, Co. Durham, DH7 0EX, UK
Central Electrocardiogram Laboratory	eResearch Technology Inc.<br>1818 Market Street, Suite 1000<br>Philadelphia, Pennsylvania 19103, USA
Statistician	Vasileios Kiroplastis, MS<br>Covance

SYNOPSIS

Title of study: A Randomised, Double-blind, Parallel-group, Placebo-controlled, Phase Ia/Ib, Multiple-site Study to Assess the Safety, Tolerability, Pharmacokinetics, and Pharmacodynamics of KL1333 after a Single Oral Dose and Multiple Ascending Oral Doses in Healthy Subjects and Patients with Primary Mitochondrial Disease

Objectives:

The primary objectives of the study are:

- To evaluate the safety and tolerability of a single oral dose, with and without food, and multiple ascending oral doses of KL1333 in healthy subjects
- To evaluate the safety and tolerability of multiple oral doses of KL1333 in patients with mitochondrial disease.

The secondary objectives of the study are:

- To determine the single oral dose plasma pharmacokinetics (PK) of KL1333 in healthy subjects, including the effect of food intake
- To determine the multiple oral dose plasma PK of KL1333 in healthy subjects and patients with mitochondrial disease.

The exploratory objectives of the study are:

- To explore the multiple-dose pharmacodynamics (PD) of KL1333 in healthy subjects and patients with mitochondrial disease using blood biomarkers
- To explore clinician- and patient-rated outcome assessments following multiple oral doses of KL1333 in patients with mitochondrial disease
- To collect blood samples for analysis of metabolomics following multiple oral doses of KL1333 in healthy subjects and patients with mitochondrial disease
- To assess the effect of KL1333 on electrocardiogram (ECG) parameters, including concentration-QT interval corrected for heart rate (QTc) analysis, in healthy subjects
- To collect blood samples for NAD(P)H:dehydrogenase [quinone]1 genotyping from healthy subjects who receive single or multiple oral doses of KL1333 and patients with mitochondrial disease who receive multiple oral doses of KL1333.

Study design:

This will be a double-blind, randomised, placebo-controlled, single and multiple oral dose study conducted in 4 parts.

Part A:

Part A will comprise a randomised, single-dose, single-sequence, placebo-controlled study. Eight healthy subjects will be studied in a single cohort (Group A1).

Potential subjects will be screened to assess their eligibility to enter the study within 28 days prior to the first dose administration. Subjects will participate in 2 treatment periods. For each treatment period, subjects will reside at the Phase I clinical site from Days -1 to 3 (48 hours postdose). Subjects will return to the clinical site for outpatient visits on Days 4 and 5. There will be at least a 10-day washout between doses (from Period 1, Day 1 to Period 2, Day 1).

Six subjects will be randomised to receive 25 mg KL1333 and 2 subjects will be randomised to receive placebo, and subjects will receive the same treatment in both treatment periods. On Treatment Period 1, Day 1, subjects will receive a single oral dose of study drug following an overnight fast of at least 8 hours. On Treatment Period 2, Day 1, subjects will receive a single oral dose of study drug after consuming a standard high-fat breakfast. Following review of safety, tolerability, and PK data, up to 2 additional dose cohorts of healthy subjects may be added if needed to determine the study treatment for Part B. Additional single-dose cohorts may be enrolled based on data obtained from either Parts A or B. If additional cohorts are required, each cohort will consist of 8 subjects, with 6 subjects receiving KL1333 and 2 subjects receiving placebo, and will undergo a single treatment period. The dose level and dietary state for administration of KL1333 in these potential additional cohorts will be decided following review of data in Part A and any available data from Part B, and the dose level could be either less than or greater than 25 mg. The

dose level will not exceed 600 mg, and the predicted exposure following a single dose in any subject in Part A will not exceed an area under the plasma concentration-time curve [AUC] from time zero to 24 hours postdose [AUC_{0-24}] of 51,800 ng.h/mL for derived total KL1333.

Subjects will return for a Follow-up visit on Day 6, 5 days after their final dose.

Part B:

Part B will comprise a randomised, multiple-dose, sequential-group, placebo-controlled study. Sixteen healthy subjects will be studied in 2 cohorts (Groups B1 and B2), with each cohort consisting of 8 subjects. Part B may start after completion of Group A1, at a dose equal to or less than given in Part A.

Potential subjects will be screened to assess their eligibility to enter the study within 28 days prior to the first dose administration. All subjects will participate in 1 treatment period and will reside at the Phase I clinical site from Days -1 to 12 (48 hours post final dose). Subjects will return to the clinical site for outpatient visits on Days 13 and 14.

On Day 1, 6 subjects will be randomised to receive KL1333 and 2 subjects will be randomised to receive placebo. The preliminary planned doses of KL1333 for Groups B1 and B2 are 25 and 50 mg, respectively, once daily (QD) on Days 1 to 10. Dose levels, dose frequency, and dietary state will be confirmed following review of safety, tolerability, and PK data from Part A and ongoing data from Part B. Additionally, a dose selection conference meeting will be held before each cohort in Part B where blinded data from the previous cohort will be reviewed before a decision is made about proceeding to the next cohort. Following review of safety, tolerability, and PK data, up to 3 additional dose cohorts of healthy subjects may be added to further explore the PK, safety, and tolerability of KL1333. If additional cohorts are required, each cohort will consist of 8 subjects, with 6 subjects receiving KL1333 and 2 subjects receiving placebo. The dose level will not exceed 600 mg, and the predicted exposure following multiple daily dose administration in any subject in Part B will not exceed an AUC_{0-24} of 51,800 ng.h/mL for derived total KL1333. There will be a minimum of 6 days between dose escalations for each cohort (between the last dose of one cohort and the first dose of the next cohort).

Subjects will return for a Follow-up visit on Day 15, 5 days after their final dose.

Part C:

Part C will comprise a randomised, multiple-dose, single-group, placebo-controlled study. A total of 8 patients diagnosed with any mitochondrial disease will be enrolled in this part of the study. Part C may start after the dose selection conference has been completed for the final cohort of Part B, at a daily dose no higher than the highest well-tolerated dose in Part B.

Potential study patients will be screened to assess their eligibility to enter the study within 75 days prior to the first dose administration. Patients will reside at the clinical site, or nearby the clinical site at a hotel recommended by the clinical site, from Days -1 to 2 and Days 10 to 11. Patients will return to the clinical site for outpatient visits on Days 4 and 8. Patients will be randomised on Day 1.

Two patients will be initially dosed, with 1 patient receiving KL1333 and 1 patient receiving placebo. If there are no safety or tolerability concerns in these patients following the Day 4 visit, the remaining 6 patients, with 5 patients receiving KL1333 and 1 patient receiving placebo, will be enrolled on a rolling basis. In the event there are safety concerns following completion of the 2 sentinel patients without meeting stopping criteria, the Sponsor may add intermediate cohorts if the safety evaluation in intermediate cohorts will be needed. The dosing schedule of intermediate cohorts will be the same as that of the planned cohort. The schedule of safety assessments of intermediate cohorts will be the same as that of the planned cohort as a general rule. Whether or not to add safety assessments in the intermediate cohorts will be determined by the Sponsor.

It is planned for patients to receive study drug QD on Days 1 to 10. Dose levels, dose frequency, and dietary state will be confirmed following review of safety, tolerability, and PK data from Part B, and unless deemed very unfavourable for the conduct of the study, the patients will not be required to be fasting prior to dosing. Study drug will be administered by clinical site staff when the patients are resident at the clinical site or return for outpatient visits. On all other days, patients will record drug administration and any concomitant medications in a diary that will be provided

to each patient. Diaries will be reviewed and checked for compliance during the outpatient visits and as part of the check-in procedures on Day 10. Clinical symptoms that occur while patients are not resident at the site will be collected by the site using standard adverse event (AE) reporting procedures.

Patients will return for a Follow-up visit on Day 15, 5 days after their final dose.

Part D:

Part D will comprise a randomised, multiple-dose, placebo-controlled study. Sixteen healthy subjects will be studied in 2 cohorts (Groups D1 and D2), with each cohort consisting of 8 subjects. Part D will start after completion of Part B, and the Part D groups may be run in parallel.

Potential subjects will be screened to assess their eligibility to enter the study within 35 days prior to the first dose administration. All subjects will participate in 1 treatment period and will reside at the Phase I clinical site from Days -1 to 12 (48 hours post final dose). Subjects will return to the clinical site for outpatient visits on Days 13 and 14.

On Day 1, 6 subjects will be randomised to receive KL1333 and 2 subjects will be randomised to receive placebo. The doses of KL1333 for Groups D1 and D2 are 75 mg twice daily (BID) and 50 mg 3 times daily (TID), respectively, on Days 1 to 10 with a single dose administration on Day 10.

Subjects will return for a Follow-up visit on Day 15, 5 days after their final dose.

Number of subjects:

Part A: 8 healthy subjects (6 active:2 placebo) will be studied in 1 cohort (Group A1). Up to 2 further cohorts of 8 healthy subjects may be included (ie, maximum of 24 healthy subjects total in Part A).

Part B: 8 healthy subjects (6 active:2 placebo) will be studied in each of 2 cohorts (Groups B1 and B2). Up to 3 further cohorts of 8 healthy subjects may be included (ie, maximum of 40 healthy subjects total in Part B).

Part C: 8 patients with mitochondrial disease (6 active:2 placebo) will be studied in 1 dosage group.

Part D: 8 healthy subjects (6 active:2 placebo) will be studied in each of 2 cohorts (Groups D1 and D2).

Total number of subjects in Parts A to D: 48 to 88 subjects.

Diagnosis and main criteria for inclusion:

Healthy male and female subjects aged between 18 and 65 years (inclusive) with a body mass index between 18.0 and 32.0 kg/m^2 (inclusive) and weight ≥50 kg.

Male and female patients with any stable mitochondrial disease aged between 18 and 75 years (inclusive) with a body mass index between 15.0 and 32.0 kg/m^2 (inclusive).

Investigational products, dose, and mode of administration:

Test products: 25 and 100 mg KL1333 encapsulated tablets and matching encapsulated placebo tablets.

Proposed dose level for Part A: 25 mg KL1333 or placebo administered once in the fasted state and once in the fed state.

Proposed dose levels for Part B: 25 and 50 mg KL1333 or placebo QD for 10 days. The dose level, dosing frequency, and dietary state for Part B will be decided, in consultation with the Sponsor, on the basis of data from Part A of the study and emerging interim data from Part B.

Patients in Part C will be administered KL1333 or placebo QD for 10 days. The dose level, dosing frequency, and dietary state for Part C will be decided, in consultation with the Sponsor, on the basis of data from Part B of the study.

Dose levels for Part D: 75 mg BID and 50 mg TID KL1333 or placebo for 10 days with a single dose administration on Day 10. The first dose on Days 1 and 7 and the dose on Day 10 will be administered in the fasted state. All other doses can be administered without regard to food.

The dose level will not exceed 600 mg, and the predicted exposure in any subject in any cohort in this study will not exceed an AUC_{0-24} of 51,800 ng.h/mL for derived total KL1333 using the revised bioanalytical method that measures KL1333 as the sum of parent KL1333, de-conjugated glucuronidated KL1333 metabolites, and sulphated KL1333 metabolites.

Duration of subject participation in the study:
Planned duration of the study for the individual (Screening to Follow-up visit): approximately 6 weeks for Cohort A1 in Part A, approximately 5 weeks for additional optional cohorts in Part A, approximately 6 weeks for Part B, and approximately 7-11 weeks for Parts C and D.

Endpoints:
Pharmacokinetics:
Blood samples for the analysis of plasma concentrations of KL1333 will be collected, and PK parameters will be derived by noncompartmental analysis.
For Part A, the PK parameters will include:

- AUC from time zero to infinity ($AUC_{0-\infty}$)
- AUC_{0-24}
- AUC from time zero to the time of the last quantifiable concentration ($AUC_{0-tlast}$)
- C_{max}
- time of the C_{max} (T_{max})
- apparent plasma terminal elimination half-life ($t_{1/2}$)
- mean residence time (MRT)
- apparent total plasma clearance (CL/F)
- apparent volume of distribution during the terminal phase (V_z/F).

For Parts B through D, the PK parameters will include:

- $AUC_{0-\infty}$ (Day 1 only)
- AUC over a dosing interval ($AUC_{0-\tau}$; Days 1 and 10)
- temporal change parameter (TCP; $AUC_{0-\tau}/AUC_{0-\infty}$)
- C_{max}
- minimum observed plasma concentration (C_{min})
- T_{max}
- $t_{1/2}$
- MRT on Days 1 and 10
- CL/F on Days 1 and 10
- V_z/F on Days 1 and 10
- observed accumulation ratio based on $AUC_{0-\tau}$ (RA_{AUC})
- observed accumulation ratio based on C_{max} (RA_{Cmax})
- peak-to-trough ratio (PTR).

Other PK parameters will be calculated if appropriate.

Pharmacodynamics:
For Parts B through D, blood biomarker assessments will include:

- nicotinamide adenine dinucleotide (oxidized form; NAD^+)/nicotinamide adenine dinucleotide (reduced form; NADH) concentrations and ratio
- fibroblast growth factor-21 (FGF21)
- growth/differentiation factor-15 (GDF15)
- lactate/pyruvate concentrations and ratio.

For Part C, blood biomarkers assessments will also include:

- glucose
- glycated albumin/albumin concentrations and ratio.

For Part C, clinician- and patient-rated assessments will include:

- Newcastle Mitochondrial Disease Adult Scale
- Clinician Global Impression
- Patient Global Impression-Improvement

- Daily Fatigue Impact Severity
- Quality of Life in Neurological Disorders Fatigue Short Form
- 30 Second Sit-to-Stand Test.

Safety:
Safety parameters will include incidence and severity of AEs; incidence of laboratory abnormalities, based on haematology, clinical chemistry, and urinalysis test results; 12-lead ECG parameters; vital signs measurements; and physical examinations.

Continuous electrocardiograms:
Continuous ECGs will be collected during Parts A and B. Endpoints for the continuous ECG evaluation will include:

- change from baseline (Δ) heart rate (HR), QT interval corrected for HR using Fridericia's method (QTcF), and PR and QRS intervals (ΔHR, ΔQTcF, ΔPR, and ΔQRS)
- placebo-corrected change from baseline (ΔΔ) in ΔHR, ΔQTcF, ΔPR, and ΔQRS (ΔΔHR, ΔΔQTcF, ΔΔPR, and ΔΔQRS)
- categorical outliers for HR and QTcF, PR, and QRS intervals
- frequency of treatment-emergent changes of T-wave morphology and U-wave presence.

Statistical methods:

Pharmacokinetics:
Individual plasma concentrations of KL1333 will be listed and summarised using descriptive statistics. Individual and mean KL1333 concentration-time profiles will be presented graphically. In Part A, where data are available, the effect of food on KL1333 will be investigated using an analysis of variance (ANOVA) model as appropriate. In Part B, where data are available, KL1333 dose proportionality will be examined across the dose cohorts. The PK parameters will be analysed for dose proportionality using a power model approach or ANOVA model as appropriate.

Pharmacodynamics:
Pharmacodynamic parameters will be listed and summarised using descriptive statistics. Formal statistical analysis of PD data in Parts C and D is planned.

Safety:
Safety parameters will be listed and summarised using descriptive statistics. The number of subjects with abnormalities in clinical laboratory measurements and the number of subjects with vital signs measurements or ECG parameters meeting criteria of potential clinical concern from baseline through the Follow-up visit will be summarised. No formal statistical analysis of safety data is planned.

Continuous electrocardiograms (Parts A and B only):
The primary analysis will be based on concentration-QTc modelling of the relationship between KL1333 and ΔQTcF with the intent to exclude an effect >10 msec at clinically relevant KL1333 plasma concentrations. In addition, the effect of KL1333 on the ΔΔQTcF will be evaluated at each postdose timepoint ('by-timepoint' analysis) using the Intersection Union Test. An analysis of categorical outliers will be performed for changes in HR; PR, QRS, and QTcF intervals; T-wave morphology; and U-wave presence.

TABLE OF CONTENTS

TITLE PAGE......1
SPONSOR APPROVAL......2
INVESTIGATOR AGREEMENT......3
STUDY IDENTIFICATION......4
SYNOPSIS......5
TABLE OF CONTENTS......10
LIST OF TABLES AND FIGURES......12
LIST OF ABBREVIATIONS......13
1. INTRODUCTION......16
1.1. Overview......16
1.2. Summary of Nonclinical Pharmacology......16
1.3. Summary of Safety Pharmacology......17
1.4. Summary of Toxicology......17
1.5. Summary of Nonclinical Pharmacokinetics......18
1.6. Summary of Clinical Experience......19
1.7. Study Rationale......23
1.8. Benefit-risk Assessment......23
2. OBJECTIVES AND ENDPOINTS......24
2.1. Objectives......24
2.2. Endpoints......24
2.2.1. Primary Endpoints......24
2.2.2. Secondary Endpoints......25
2.2.3. Exploratory Endpoints......25
3. INVESTIGATIONAL PLAN......26
3.1. Overall Study Design and Plan......28
3.1.1. Part A......28
3.1.2. Part B......30
3.1.3. Part C......31
3.1.4. Part D......33
3.2. Study Start and End of Study Definitions......34
3.3. Additional Groups......35
3.4. Discussion of Study Design, Including the Choice of Control Groups......35
3.4.1. Dose Interval......36
3.5. Selection of Doses in the Study......36
3.6. Dose Escalation......38
3.7. Dose Escalation Stopping Criteria......39
4. SELECTION OF STUDY POPULATION......40
4.1. Inclusion Criteria......40

4.2. Exclusion Criteria 41
4.3. Subject Number and Identification 44
4.4. Subject Withdrawal and Replacement 44
4.5. Study Termination 44
5. STUDY TREATMENTS 45
5.1. Description, Storage, Packaging, and Labelling 45
5.2. Study Treatment Administration 45
5.3. Randomisation 45
5.4. Blinding 46
5.5. Treatment Compliance 46
5.6. Drug Accountability 47
6. CONCOMITANT THERAPIES AND OTHER RESTRICTIONS 47
6.1. Concomitant Therapies 47
6.2. Diet 48
6.3. Smoking 49
6.4. Exercise 49
6.5. Blood Donation 49
7. STUDY ASSESSMENTS AND PROCEDURES 49
7.1. Pharmacokinetic Assessments 50
7.1.1. Sample Collection and Processing 50
7.1.2. Analytical Methodology 50
7.2. Pharmacodynamic Assessments 50
7.2.1. Sample Collection and Processing 50
7.2.2. Analytical Methodology 51
7.2.3. Clinical Assessments 51
7.3. Safety and Tolerability Assessments 52
7.3.1. Adverse Events 52
7.3.2. Clinical Laboratory Evaluations 53
7.3.3. General and Gastrointestinal Inflammation Assessments 53
7.3.4. Vital Signs 53
7.3.5. Electrocardiogram 54
7.3.6. Physical Examination 56
7.3.7. Body Weight 56
7.3.8. Genotyping 56
7.3.9. Exit Interview 57
8. SAMPLE SIZE AND STATISTICAL DATA ANALYSIS 57
8.1. Determination of Sample Size 57
8.2. Analysis Populations 57
8.2.1. Pharmacokinetic Population 57
8.2.2. Pharmacodynamic Population 57

8.2.3. Safety Population 57
8.2.4. QT/QTc Population 57
8.2.5. PK/QTc Population 57
8.3. Pharmacokinetic Analyses 58
8.4. Pharmacodynamic Analyses 58
8.5. Safety Analysis 58
8.5.1. Cardiodynamic Electrocardiogram Evaluation 59
8.6. Interim Analysis 62
9. REFERENCES 62
10. APPENDICES 64
Appendix 1: Adverse Event Reporting 65
Appendix 2: Clinical Laboratory Evaluations 69
Appendix 3: Contraception Guidance 70
Appendix 4: Regulatory, Ethical, and Study Oversight Considerations 73
Appendix 5: Schedule of Assessments 76

LIST OF TABLES AND FIGURES

Table 1: Summary of Adverse Events in Healthy Subjects from Study KL1333_101 20
Table 2: Mean (± Standard Deviation) Pharmacokinetics Parameters of Free KL1333 in Study KL1333_101 22
Table 3: Mean (± Standard Deviation) Pharmacokinetics Parameters of Total KL1333 in Study KL1333_101 23
Table 4: Proposed Investigational Medicinal Product Dose Levels for Parts A and B 37
Table 5: High-fat Breakfast Content 48
Table 6: T-wave Morphology Categories (Assessed Manually) 56

Figure 1: Study Dose Levels 27
Figure 2: Study Schematic (Cohort A1, Fasted versus Fed [High-fat Breakfast] and Additional Optional Cohorts A2 and A3) 29
Figure 3: Study Schematic (Part B) 31
Figure 4: Study Schematic (Part C) 33
Figure 5: Study Schematic (Part D) 34

LIST OF ABBREVIATIONS

Abbreviation	Definition
AE	adverse event
AMPK	5'-adenosine monophosphate-activated protein kinase
ANOVA	analysis of variance
ATP	adenosine triphosphate
AUC	area under the plasma concentration-time curve
AUC_{0-24}	area under the plasma concentration-time curve from time zero to 24 hours postdose
$AUC_{0-\infty}$	area under the plasma concentration-time curve from time zero to infinity
$AUC_{0-tlast}$	area under the plasma concentration-time curve from time zero to the time of the last quantifiable concentration
$AUC_{0-\tau}$	area under the plasma concentration-time curve over a dosing interval
BID	twice daily
CFR	Code of Federal Regulations
CGI	Clinician Global Impression
CI	confidence interval
CL/F	apparent total plasma clearance
C_{max}	maximum observed plasma concentration
C_{min}	minimum observed plasma concentration
CNS	central nervous system
crif1	CR6-interacting factor 1
CRO	Contract Research Organisation
CRP	C-reactive protein
CYP	cytochrome P450
Δ	change from baseline
ΔΔ	placebo-corrected change from baseline
D-FIS	Daily Fatigue Impact Severity
DSS	Drug Safety Services
EC	Ethics Committee
EC_{50}	dose that produces half of the maximum effect attributable to the drug
ECG	electrocardiogram
eCRF	electronic Case Report Form
EDC	electronic data capture
E_{max}	maximum effect attributable to the drug
ESR	erythrocyte sedimentation rate
FGF21	fibroblast growth factor-21
FSH	follicle-stimulating hormone
GCP	Good Clinical Practice

GDF15	growth/differentiation factor-15
HR	heart rate
IB	Investigator's Brochure
IC_{50}	half-maximal inhibitory concentration
ICF	Informed Consent Form
ICH	International Council for/Conference on Harmonisation
IMP	investigational medicinal product
LS	least squares
MELAS	Mitochondrial Encephalomyopathy with Lactic Acidosis and Stroke-like episodes
MN	micronucleus
MRT	mean residence time
NAD^+	nicotinamide adenine dinucleotide (oxidized form)
NADH	nicotinamide adenine dinucleotide (reduced form)
Neuro-QoL Fatigue	Quality of Life in Neurological Disorders Fatigue
NMDAS	Newcastle Mitochondrial Disease Adult Scale
NOAEL	no observed adverse effect level
NQO1	NAD(P)H:dehydrogenase [quinone]1
PD	pharmacodynamic(s)
PGC-1α	peroxisome proliferator-activated receptor gamma coactivator 1-alpha
PGI-I	Patient Global Impression-Improvement
PK	pharmacokinetic(s)
PTR	peak-to-trough ratio
QD	once daily
QTc	QT interval corrected for heart rate
QTcF	QT interval corrected for heart rate using Fridericia's method
RA_{AUC}	observed accumulation ratio based on area under the plasma concentration-time curve over a dosing interval
RA_{Cmax}	observed accumulation ratio based on maximum observed plasma concentration
ROS	reactive oxygen species
SAE	serious adverse event
SAP	Statistical Analysis Plan
SD	standard deviation
SE	standard error
SIRT1	sirtuin 1
SUSAR	Suspected Unexpected Serious Adverse Reaction
$t_{1/2}$	apparent plasma terminal elimination half-life
TCP	temporal change parameter
TEAE	treatment-emergent adverse event

TID	3 times daily
T_{max}	time of the maximum observed plasma concentration
TMF	Trial Master File
V_z/F	apparent volume of distribution during the terminal phase

1. INTRODUCTION

1.1. Overview

Mitochondria are important organelles that generate most of the energy required by the human body in the form of adenosine triphosphate (ATP) via the electron transport chain. Primary mitochondrial diseases are generally triggered by dysfunction of the electron transport chain, resulting in disorders in mitochondrial energy production or excessive reactive oxygen species (ROS) generation. Hundreds of primary mitochondrial diseases are known, including Mitochondrial Encephalomyopathy with Lactic Acidosis and Stroke-like episodes (MELAS), Leber Hereditary Optic Neuropathy, Myoclonic Epilepsy with Ragged-Red Fibers, and Leigh syndrome. Clinical manifestations of primary mitochondrial diseases cover a wide spectrum of phenotypes including serious and life-threatening conditions such as organ failure, cardiorespiratory arrest, intracranial haemorrhage, leukaemia/lymphoma, myocardial ischaemia, intestinal obstruction, and immune deficiency, as well as an even wider range of other potentially debilitating conditions.

KL1333 is a novel compound under development for primary mitochondrial diseases. KL1333 acts as a substrate for NAD(P)H:dehydrogenase [quinone]1 (NQO1), which produces nicotinamide adenine dinucleotide (oxidized form; NAD^+) by transferring 2 electrons to KL1333 using nicotinamide adenine dinucleotide (reduced form; NADH) as a cofactor. KL1333 transfers these electrons to the mitochondrial electron transport system, directly promoting ATP production. Additionally, the elevated NAD^+ levels lead to activation of mitochondrial biogenesis pathways, such as sirtuin 1 (SIRT1), 5'-adenosine monophosphate-activated protein kinase (AMPK), and peroxisome proliferator-activated receptor gamma coactivator 1-alpha (PGC-1α), thereby improving mitochondrial function.

1.2. Summary of Nonclinical Pharmacology

KL1333 was shown to be a more potent substrate to NQO1 than other NQO1 active compounds developed for primary mitochondrial disease (ie, idebenone). In cellular models, including cells derived from patients with MELAS, KL1333 demonstrated increased ATP; decreased ROS; decreased lactic acid; increased NAD^+; activation of SIRT1, AMPK, and PGC-1α; and improved mitochondrial oxidative phosphorylation function.

There are multiple animal models with genetic mutations affecting different parts of the mitochondrial respiratory chain. KL1333 was evaluated in an animal model of primary mitochondrial myopathy, a conditional knockout of CR6-interacting factor 1 (c*rif1*) in skeletal muscle tissue. The c*rif1* knockout mice exhibited reduced mitochondrial oxidative phosphorylation function in skeletal muscle, progressive muscle weakness, and increased levels of the mitokines fibroblast growth factor-21 (FGF21) and growth/differentiation factor-15 (GDF15). KL1333 restored the relative grip strength in these animals and attenuated the increased FGF21 and GDF15 levels.

KL1333 has also been evaluated in animal models where mitochondrial function is compromised, but where the disease is not caused by a primary mitochondrial oxidative phosphorylation defect, such as diabetes, Duchenne muscle dystrophy, and diet-induced obesity models, and has demonstrated beneficial effects on metabolism.

1.3. Summary of Safety Pharmacology

Male Sprague Dawley rats were given a single oral dose of KL1333 up to 1400 mg/kg and effects on the central nervous system (CNS) were investigated using a modified Irwin Test with measurement of rectal temperature predose and immediately following each Irwin observation. Oral administration of 350, 700, or 1400 mg/kg KL1333 had no effect on the CNS or body temperature compared with the control group. Thus, the no observed effect level was 1400 mg/kg. The effects of KL1333 on the human ether-a-go-go-related gene tail currents in stably transfected human embryonic kidney 293 cells was investigated at concentrations up to 300 μM. At concentrations of 30 μM or higher, KL1333 significantly inhibited the tail currents. Based on the dose-response curve, the estimated half-maximal inhibitory concentration (IC_{50}) value was determined to be 138.31 μM. Male beagle dogs were given a single oral dose of KL1333 up to 400 mg/kg to determine potential cardiovascular effects. There were no effects on the electrocardiogram (ECG; including QT and QT interval corrected for heart rate [HR] using Fridericia's method [QTcF]) and no changes in haemodynamic parameters or body temperature. Male Sprague Dawley rats were given a single oral dose of KL1333 up to 1400 mg/kg and effects on the respiratory system were investigated using whole body plethysmography. Oral administration of KL1333 at dose levels of 350, 700, and 1400 mg/kg caused a number of changes in respiratory parameters, predominantly a transient decrease in respiration rate occurred from 30 to 90 minutes postdose. However, none of these changes were considered adverse, and so the no observed adverse effect level (NOAEL) was 1400 mg/kg.

1.4. Summary of Toxicology

In a 4-week, repeat-dose rat study at doses up to 160 mg/kg/day, extramedullary haemopoiesis was noted in the spleen in rats given 160 mg/kg/day and squamous epithelial hyperplasia of the forestomach and mucosal hyperplasia of the intestinal tracts occurred in rats given 80 or 160 mg/kg/day, and 1 death of undetermined cause occurred at 160 mg/kg/day. These findings may have been due to irritation of the digestive tract by KL1333 rather than toxicity since they were not evident in all cases and were reversible. In a second 4-week, repeat-dose rat study at doses up to 120 mg/kg/day, there were no deaths or adverse clinical signs, and there were no macroscopic or microscopic findings attributed to administration of KL1333. The NOAEL was determined to be 120 mg/kg/day for both sexes.

A 13-week, repeat-dose rat study was conducted at doses up to 270 mg/kg/day. Dead animals were observed in males given ≥120 mg/kg/day and in all test article groups (ie, ≥80 mg/kg/day) in females. The primary finding was ulceration of the gastrointestinal tract, neutrophil infiltrate, hyperplasia of squamous epithelium, and hyperkeratin at ≥120 mg/kg/day in males and ≥80 mg/kg/day in females. There was evidence of reversibility of these changes, which may also have been associated with the physiological irritation potential of KL1333 on the gastrointestinal tract and ultimately resulted in morbidity and death. Clinical signs and clinical pathology changes were consistent with effects on the gastrointestinal tract and/or the poor condition of the animals and were observed in both sexes across all groups. The NOAEL was 80 mg/kg/day in males and was not determined (ie, <80 mg/kg/day) in females.

In the 4-week, repeat-dose dog study with KL1333 at doses up to 400 mg/kg/day, KL1333 was administered for only up to 14 days due to vomiting, liquid faeces, and consequent

adverse effects on food consumption and body weight. These adverse findings are likely due to the irritant effects of KL1333 on the gastrointestinal tract of dogs, and the NOAEL was not determined. In the 13-week, repeat-dose study in dogs, KL1333 doses were reduced to a maximum of 40 mg/kg/day. Sporadic clinical signs of soft stool, diarrhoea, and vomiting occurred in all KL1333-treated groups and were reversible. There were no effects on body weight or food consumption or changes in any other observation or examination items. The NOAEL was determined to be 40 mg/kg/day for both sexes, and the target organ was not identified.

In genotoxicity studies, KL1333 was not mutagenic in 5 bacterial strains in the bacterial reverse mutation assay in the absence or presence of S9. KL1333 induced structural chromosome aberrations in human peripheral blood lymphocytes when tested for 3+17 hours in the absence and presence of S9 and for 20+0 hours in the absence of S9.

KL1333 was negative in the in vivo micronucleus (MN) test in male rats, but did induce DNA strand breakage in comet analysis of the liver and duodenum following oral gavage administrations at 1400 mg/kg/day for 3 days. In a second confirmatory comet assay up to 1400 mg/kg/day in rats, KL1333 also induced DNA strand breakage in the liver at the highest dose and showed that the no observable genotoxic effect level is 250 to 350 mg/kg in rats. The DNA damage in the duodenum may be secondary to localised toxic effects and an inflammatory response, whereas the genotoxic response in the liver may be due to direct damage to DNA. An additional MN assay was performed in male rats dosed up to 200 mg/kg/day for 28 days to determine if KL1333 induced MN in the liver. The MN frequencies in KL1333-treated rats were similar to control, and there was no evidence of hepatocyte toxicity. Thus, the DNA strand breaks observed in the comet assay were not converted to chromosomal breaks that would appear as MN. As 5 rats died or became moribund by Day 21, blood samples were collected from surviving rats on Day 22. The mean maximum observed plasma concentration (C_{max}) and area under the plasma concentration-time curve (AUC) from time zero to 24 hours postdose (AUC_{0-24}) of derived total KL1333 were 15,700 ng/mL and 259,000 ng.hr/mL, respectively, after dosing with 200 mg/kg/day.

KL1333 was assayed for its ability to absorb light within the range of natural sunlight (290 to 700 nm). As the molar extinction coefficient for KL1333 (0.0001 to 0.1 mg/mL) was 6298.6 L mol^{-1} cm^{-1} at 297 nm and 1440.5 L mol^{-1} cm^{-1} at 450 nm, the potential for phototoxic effects could not be ruled out. KL1333 (0 to 100 µg/mL) was evaluated in the presence and absence of UV-A irradiation in a 3T3 Neutral Red Uptake assay and found to be negative for phototoxicity.

1.5. Summary of Nonclinical Pharmacokinetics

KL1333 reacts rapidly with NQO1 to form KL1333·H_2 that is unstable in plasma and either rapidly conjugated to various glucuronide and sulphate conjugates by phase 2 metabolizing enzymes, which are then released into the blood, or converted back to parent KL1333. It has been determined that the KL1333-glucuronides are short lived and rapidly convert back to KL1333, while the KL1333-sulphates are more stable. To measure the total amount of KL1333 in plasma, a 2-analyte method is used to quantify KL1333 as the sum of (1) parent KL1333 and the de-conjugated glucuronidated metabolite(s), and (2) separately (but in the same assay), the sulphated KL1333 metabolite(s). Consequently, derived total KL1333 in

plasma is a measurement of parent KL1333, de-conjugated glucuronidated KL1333 metabolites, and sulphated KL1333 metabolites.

In vivo drug-drug interaction studies in rats showed that KL1333 increased the exposure to a cytochrome P450 (CYP) 1A substrate drug and decreased exposure to a CYP2B substrate drug. In human hepatocytes in vitro, KL1333 did not induce CYP3A, CYP1A, or CYP2B6 at concentrations up to 2 µM. In human liver microsomes, KL1333 at concentrations of up to 100 µM inhibited the enzyme activities of all tested CYPs, and inhibition by KL1333 was shown to be metabolism-dependent for the majority of them. The IC_{50} values for the tested CYPs (CYP2B6, CYP2C8, CYP2C9, CYP2C19, CYP2D6, CYP3A4) were between 0.358 and 3.72 µM. The most potently inhibited were CYP1A2 and CYP3A4 (using testosterone as a substrate), with an IC_{50} of 0.673 and 0.936 µM, respectively, for reversible inhibition and 0.358 and 0.981 µM, respectively, for metabolism-dependent inhibition.

1.6. Summary of Clinical Experience

KL1333 has been administered to 60 healthy male subjects in a first-in-human, randomised, double-blind, placebo-controlled, single ascending dose study in Korea. A preliminary review of the data suggests that a single-dose administration of KL1333, ranging from 25 to 600 mg was well tolerated and a single dose of 800 mg was moderately tolerated. All adverse events (AEs) were considered by the Investigator to be mild in severity and resolved by the end of the study. There were no severe AEs and no subjects discontinued from the study as a result of an AE. The most common system organ class overall was gastrointestinal disorders, with multiple subjects reporting AEs of abdominal discomfort, abdominal distension, abdominal pain, diarrhoea, and nausea. The treatment-emergent AEs (TEAEs) are summarised by preferred term in Table 1.

Table 1: Summary of Adverse Events in Healthy Subjects from Study KL1333_101

	25 mg (N = 4)	50 mg (N = 4)	100 mg (N = 8)	200 mg (N = 8)	400 mg (N = 8)	600 mg (N = 8)	800 mg (N = 8)	Placebo (N = 12)	Total (N = 60)
Number of subjects with TEAE	--	1(25.0)[1]	1(12.5)[1]	4(50.0)[8]	2(25.0)[6]	3(37.5)[7]	6(75.0)[25]	3(25.0)[4]	20(33.3)[52]
Gastrointestinal disorders	--	--	1(12.5)[1]	1(12.5)[3]	2(25.0)[5]	3(37.5)[7]	6(75.0)[18]	--	13(21.7)[34]
Diarrhoea	--	--	--	1(12.5)[1]	2(25.0)[2]	3(37.5)[3]	6(75.0)[7]	--	12(25.0)[13]
Abdominal discomfort	--	--	1(12.5)[1]	--	1(12.5)[1]	--	3(37.5)[3]	--	5(8.3)[5]
Abdominal distension	--	--	--	--	--	2(25.0)[2]	3(37.5)[3]	--	5(8.3)[5]
Abdominal pain	--	--	--	--	1(12.5)[1]	2(25.0)[2]	2(25.0)[2]	--	5(8.3)[5]
Nausea	--	--	--	--	1(12.5)[1]	--	2(25.0)[2]	--	3(5.0)[3]
Abdominal pain upper	--	--	--	1(12.5)[1]	--	--	--	--	1(1.7)[1]
Aphthous ulcer	--	--	--	1(12.5)[1]	--	--	--	--	1(1.7)[1]
Haematochezia	--	--	--	--	--	--	1(12.5)[1]	--	1(1.7)[1]
Respiratory, thoracic and mediastinal disorders	--	1(25.0)[1]	--	2(25.0)[2]	--	--	--	2(16.7)[2]	5(8.3)[5]
Rhinorrhoea	--	1(25.0)[1]	--	2(25.0)[2]	--	--	--	1(8.3)[1]	4(6.7)[4]
Cough	--	--	--	--	--	--	--	1(8.3)[1]	1(1.7)[1]
Nervous system disorders	--	--	--	1(12.5)[1]	1(12.5)[1]	--	1(12.5)[2]	--	3(5.0)[4]
Dizziness	--	--	--	--	--	--	1(12.5)[2]	--	1(1.7)[2]
Dizziness postural	--	--	--	--	1(12.5)[1]	--	--	--	1(1.7)[1]
Headache	--	--	--	1(12.5)[1]	--	--	--	--	1(1.7)[1]
General disorders and administration site conditions	--	--	--	--	--	--	1(12.5)[1]	1(8.3)[1]	2(3.3)[2]
Fatigue	--	--	--	--	--	--	1(12.5)[1]	--	1(1.7)[1]
Secretion discharge	--	--	--	--	--	--	--	1(8.3)[1]	1(1.7)[1]
Infections and infestations	--	--	--	1(12.5)[1]	--	--	1(12.5)[1]	--	2(3.3)[2]
Gastroenteritis	--	--	--	1(12.5)[1]	--	--	--	--	1(1.7)[1]
Upper respiratory tract infection	--	--	--	--	--	--	1(12.5)[1]	--	1(1.7)[1]
Musculoskeletal and connective tissue disorders	--	--	--	1(12.5)[1]	--	--	1(12.5)[1]	--	2(3.3)[2]
Arthralgia	--	--	--	1(12.5)[1]	--	--	--	--	1(1.7)[1]
Myalgia	--	--	--	--	--	--	1(12.5)[1]	--	1(1.7)[1]
Renal and urinary disorders	--	--	--	--	--	--	2(25.0)[2]	--	2(3.3)[2]
Oliguria	--	--	--	--	--	--	2(25.0)[2]	--	2(3.3)[2]

	25 mg (N = 4)	50 mg (N = 4)	100 mg (N = 8)	200 mg (N = 8)	400 mg (N = 8)	600 mg (N = 8)	800 mg (N = 8)	Placebo (N = 12)	Total (N = 60)
Skin and subcutaneous tissue disorders	--	--	--	--	--	--	--	1(8.3)[1]	1(1.7)[1]
Erythema	--	--	--	--	--	--	--	1(8.3)[1]	1(1.7)[1]

Abbreviation: TEAE: treatment-emergent adverse event.
Note: A subject was counted once at the maximum severity if the subject reported one or more events.
Data are presented as the number of subjects who reported TEAEs (the proportion of subjects who reported TEAEs) [the number of TEAEs].

Previous bioanalytical methods were used to measure the amounts of free (parent) KL1333 and total KL1333, which included both parent KL1333 and de-conjugated glucuronide and sulphate metabolites. Following single doses from 25 to 800 mg KL1333, the C_{max} and AUC values for free KL1333 and the C_{max} values for total KL1333 appeared to increase in an approximately dose-proportional manner (Table 2). The AUCs for total KL1333 appeared to increase in a dose-proportional manner following single doses from 50 to 800 mg (Table 3).

It was subsequently determined that due to the instability of the glucuronide metabolites in both in vitro and ex vivo samples, the free KL1333 assay could not be considered a reliable measure of free KL1333 exposure in vivo and the free KL1333 concentration may have been overestimated. Conversely, the sulphate metabolites were found to be more stable; the de-conjugation of the sulphate metabolites only goes to approximately 50% completion, thus the total KL1333 concentration may have been underestimated.

Table 2: Mean (± Standard Deviation) Pharmacokinetics Parameters of Free KL1333 in Study KL1333_101

	25 mg (N = 4)	50 mg (N = 4)	100 mg (N = 8)	200 mg (N = 8)	400 mg (N = 8)	600 mg (N = 8)	800 mg (N = 8)
C_{max} (ng/mL)	6.77 ± 6.39	7.59 ± 4.2	16.29 ± 7.62	24.68 ± 14.43	62.03 ± 11.74	119.4 ± 46.46	177.63 ± 61.7
$AUC_{0\text{-tlast}}$ (ng.h/mL)	24.06 ± 5.55	41.38 ± 13.79	104.71 ± 22.53	203.00 ± 39.78	471.92 ± 210.87	478.52 ± 101.00	723.28 ± 239.68
$AUC_{0\text{-}\infty}$ (ng.h/mL)	26.47 ± 6.51	53.60 ± 26.74	111.00 ± 24.79	205.99 ± 40.80	491.14 ± 213.05	477.71 ± 109.97	734.59 ± 234.43
V_z/F (L)	18324.32 ± 6241.84	33780.5 ± 18982.24	44087.88 ± 37887.22	25300.59 ± 13461.38	41540.28 ± 30767.18	37750.88 ± 3829.53	55544.75 ± 51778.81
CL/F (L/h)	999.35 ± 301.74	1122.1 ± 530.42	942.25 ± 218.13	1003.62 ± 191.41	961.78 ± 439.2	1320.42 ± 328.15	1190.8 ± 381.18
T_{max} (h)[a]	1.25 (0.5 – 2)	1 (0.5 – 2)	1 (0.5 – 2)	2.01 (1 – 3)	2 (1 – 4.02)	2 (1 – 3)	2 (1 – 3)
$t_{1/2}$ (h)	13.73 ± 6.55	23.57 ± 14.11	34.64 ± 37.08	17.33 ± 7.68	33.98 ± 26.41	20.17 ± 7.21	31.23 ± 26.28

Abbreviations: $AUC_{0\text{-}\infty}$ = area under the plasma concentration-time curve from time zero to infinity; $AUC_{0\text{-tlast}}$ = area under the plasma concentration-time curve from time zero to the time of the last quantifiable concentration; CL/F = apparent total plasma clearance; C_{max} = maximum observed plasma concentration; $t_{1/2}$ = apparent plasma terminal elimination half-life; T_{max} = time of the maximum observed plasma concentration; V_z/F = apparent volume of distribution during the terminal phase.

[a] Median (minimum-maximum).

Table 3: Mean (± Standard Deviation) Pharmacokinetics Parameters of Total KL1333 in Study KL1333_101

	25 mg (N = 4)	50 mg (N = 4)	100 mg (N = 8)	200 mg (N = 8)	400 mg (N = 8)	600 mg (N = 8)	800 mg (N = 8)
C_{max} (ng/mL)	294.60 ± 214.16	365.01 ± 125.96	980.18 ± 315.88	1385.12 ± 800.79	2977.67 ± 1111.25	5078.81 ± 2649.28	8247.30 ± 3354.44
$AUC_{0\text{-tlast}}$ (ng.h/mL)	2503.93 ± 680.27	4090.94 ± 1223.35	9579.37 ± 1340.25	12860.68 ± 3460.51	21769.3 ± 6244.16	27173.77 ± 10359.42	38626.53 ± 9965.50
$AUC_{0\text{-}\infty}$ (ng.h/mL)	2978.21 ± 1045.48	5240.32 ± 2846.03	10130.97 ± 1735.47	13447.99 ± 3551.66	24593.09 ± 9440.25	28717.17 ± 10180.37	43220.35 ± 10461.06
V_z/F (L)	301.30 ± 148.13	330.42 ± 83.86	351.79 ± 132.64	557.08 ± 314.47	864.38 ± 567.77	1122.91 ± 1046.53	1625.44 ± 1988.62
CL/F (L/h)	9.48 ± 4.24	11.34 ± 4.64	10.11 ± 1.66	15.92 ± 4.75	18.47 ± 7.01	24.01 ± 10.62	19.62 ± 5.32
T_{max} (h)[a]	1.25 (0.5 – 2)	1 (0.5 – 2)	1 (0.5 – 2)	2.01 (1 – 3)	2 (1 – 4.02)	2 (1 – 3)	2 (1 – 3)
$t_{1/2}$ (h)	23.94 ± 13.79	24.95 ± 16.29	24.83 ± 10.61	24.21 ± 12.89	42.06 ± 46.79	33.82 ± 32.12	68.83 ± 103.18

Abbreviations: $AUC_{0\text{-}\infty}$ = area under the plasma concentration-time curve from time zero to infinity; $AUC_{0\text{-tlast}}$ = area under the plasma concentration-time curve from time zero to the time of the last quantifiable concentration; CL/F = apparent total plasma clearance; C_{max} = maximum observed plasma concentration; $t_{1/2}$ = apparent plasma terminal elimination half-life; T_{max} = time of the maximum observed plasma concentration; V_z/F = apparent volume of distribution during the terminal phase.

[a] Median (minimum-maximum).

1.7. Study Rationale

KL1333 has been administered in a single ascending dose study in Korean subjects. The principal aim of this study is to obtain safety and tolerability data when KL1333 is administered orally as a single dose and as multiple doses to healthy, non-Korean subjects and as multiple doses to patients with mitochondrial disease. This information, together with the pharmacokinetic (PK) data, will help establish the doses and dosing regimen suitable for future studies in patients. The study will also investigate the effects of food on the PK of a single dose of KL1333 in healthy subjects prior to multiple-dose administration to confirm the dietary state for dosing. This study will also investigate the effect of KL1333 on biomarkers following multiple doses in healthy subjects and patients with mitochondrial disease and the effect of KL1333 on clinician- and patient-rated outcome assessments following multiple doses in patients with mitochondrial disease.

1.8. Benefit-risk Assessment

Healthy subjects in the current study will not receive any health benefit (beyond that of an assessment of their medical status) from participating in the study.

The patients with mitochondrial disease randomised to active treatment might clinically benefit from receiving multiple doses of a potential new treatment for their disease. It is possible that by participating in this study the condition of the patients with mitochondrial disease could possibly temporarily improve, but this cannot be guaranteed.

The risks of participation are primarily those associated with adverse reactions to the investigational medicinal product (IMP), although there may also be some discomfort from collection of blood samples and other study procedures. More information about the known

and expected benefits, risks, and reasonably anticipated AEs associated with KL1333 may be found in the Investigator's Brochure (IB).[1]

2. OBJECTIVES AND ENDPOINTS

2.1. Objectives

The primary objectives of the study are:

- To evaluate the safety and tolerability of a single oral dose, with and without food, and multiple ascending oral doses of KL1333 in healthy subjects
- To evaluate the safety and tolerability of multiple oral doses of KL1333 in patients with mitochondrial disease.

The secondary objectives of the study are:

- To determine the single oral dose plasma PK of KL1333 in healthy subjects, including the effect of food intake
- To determine the multiple oral dose plasma PK of KL1333 in healthy subjects and patients with mitochondrial disease.

The exploratory objectives of the study are:

- To explore the multiple-dose pharmacodynamics (PD) of KL1333 in healthy subjects and patients with mitochondrial disease using blood biomarkers
- To explore clinician- and patient-rated outcome assessments following multiple oral doses of KL1333 in patients with mitochondrial disease
- To collect blood samples for analysis of metabolomics following multiple oral doses of KL1333 in healthy subjects and patients with mitochondrial disease
- To assess the effect of KL1333 on ECG parameters, including concentration-QT interval corrected for HR (QTc) analysis, in healthy subjects
- To collect blood samples for NQO1 genotyping from healthy subjects who receive single or multiple oral doses of KL1333 and patients with mitochondrial disease who receive multiple oral doses of KL1333.

2.2. Endpoints

2.2.1. Primary Endpoints

The primary safety endpoints for this study are as follows:

- incidence and severity of AEs
- incidence of laboratory abnormalities, based on haematology, clinical chemistry, and urinalysis test results
- 12-lead ECG parameters

- vital signs measurements
- physical examinations.

2.2.2. Secondary Endpoints

For Part A, the single-dose and food-effect (fed versus fasted dietary status at dosing) PK outcome endpoints of KL1333 are as follows:

- AUC from time zero to infinity ($AUC_{0-\infty}$)
- AUC_{0-24}
- AUC from time zero to the time of the last quantifiable concentration ($AUC_{0-tlast}$)
- C_{max}
- time of the C_{max} (T_{max})
- apparent plasma terminal elimination half-life ($t_{1/2}$)
- mean residence time (MRT)
- apparent total plasma clearance (CL/F)
- apparent volume of distribution during the terminal phase (V_z/F).

For Parts B through D, the multiple-dose PK outcome endpoints of KL1333 are as follows:

- $AUC_{0-\infty}$ (Day 1 only)
- AUC over a dosing interval ($AUC_{0-\tau}$; Days 1 and 10)
- temporal change parameter (TCP; $AUC_{0-\tau}/AUC_{0-\infty}$)
- C_{max}
- minimum observed plasma concentration (C_{min})
- T_{max}
- $t_{1/2}$
- MRT on Days 1 and 10
- CL/F on Days 1 and 10
- V_z/F on Days 1 and 10
- observed accumulation ratio based on $AUC_{0-\tau}$ (RA_{AUC})
- observed accumulation ratio based on C_{max} (RA_{Cmax})
- peak-to-trough ratio (PTR).

Other PK parameters may also be calculated if appropriate.

2.2.3. Exploratory Endpoints

For Parts B through D, the multiple-dose PD outcome endpoints are as follows:

- NAD^+/NADH concentrations and ratio
- FGF21
- GDF15
- lactate/pyruvate concentrations and ratio.

For Part C, the multiple-dose PD outcome endpoints will also include:

- glucose
- glycated albumin/albumin concentrations and ratio.

For Part C, the clinician- and patient-rated outcome endpoints are as follows:

- Newcastle Mitochondrial Disease Adult Scale (NMDAS)
- Clinician Global Impression (CGI)
- Patient Global Impression-Improvement (PGI-I)
- Daily Fatigue Impact Severity (D-FIS)
- Quality of Life in Neurological Disorders Fatigue (Neuro-QoL Fatigue) Short Form
- 30 Second Sit-to-Stand Test.

For Parts A and B, continuous ECGs will be collected, and endpoints for the continuous ECG evaluation will include:

- change from baseline (Δ) HR and QTcF, PR, and QRS intervals (ΔHR, ΔQTcF, ΔPR, and ΔQRS)
- placebo-corrected change from baseline (ΔΔ) in ΔHR, ΔQTcF, ΔPR, and ΔQRS (ΔΔHR, ΔΔQTcF, ΔΔPR, and ΔΔQRS)
- categorical outliers for HR and QTcF, PR, and QRS intervals
- frequency of treatment-emergent changes of T-wave morphology and U-wave presence.

3. INVESTIGATIONAL PLAN

This will be a double-blind, randomised, placebo-controlled, single and multiple oral dose study conducted in 4 parts. An overview of the planned dose levels for all parts of the study is shown in Figure 2.

Figure 1: Study Dose Levels

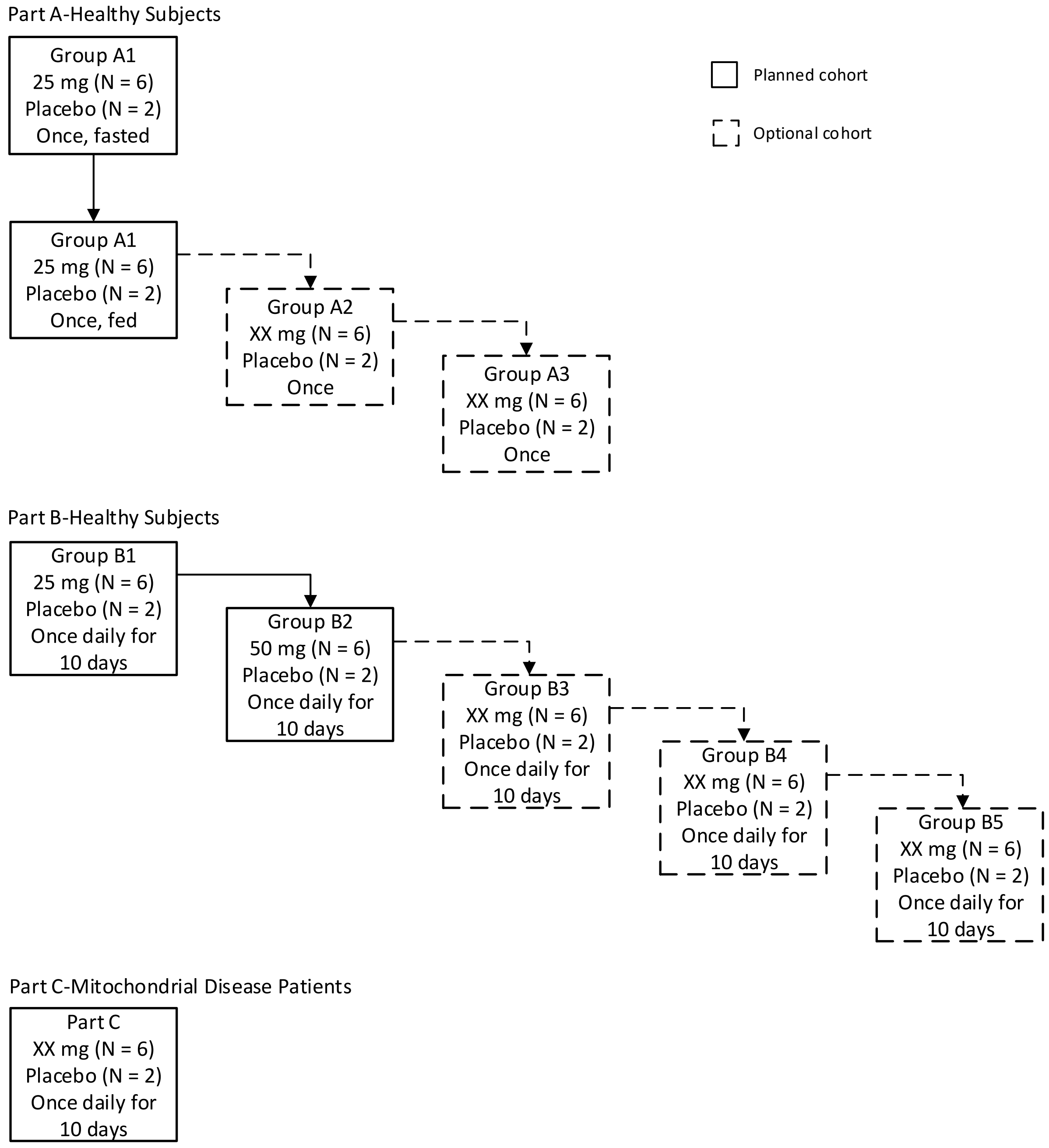

Part D-Healthy Subjects

Group D1
75 mg (N = 6)
Placebo (N = 2)
Twice daily for
10 days

Group D2
50 mg (N = 6)
Placebo (N = 2)
Three times
daily for 10 days

Note: Additional single-dose cohorts Groups A2 and A3 may be enrolled based on data obtained from either Parts A or B. If needed, dose level and dietary state will be determined from review of data in Part A and available data from Part B.
For Part B, dose levels, dose frequency, and dietary state will be confirmed from review of data in Part A and available data from Part B.
For Part C, dose levels, dose frequency, and dietary state will be confirmed following review of data from Part B, and unless deemed very unfavourable for the conduct of the study, the patients will not be required to be fasting prior to dosing.
For Part D, subjects will be fasted for the first dose on Day 1 and single dose on Day 10 for intensive pharmacokinetic sampling and the first dose on Day 7 for clinical laboratory evaluations. All other doses may be given without regard to food.

3.1. Overall Study Design and Plan

3.1.1. Part A

Part A will comprise a randomised, single-dose, single-sequence, placebo-controlled study. Eight healthy subjects will be studied in a single cohort (Group A1).

Potential subjects will be screened to assess their eligibility to enter the study within 28 days prior to the first dose administration. Subjects will participate in 2 treatment periods. For each treatment period, subjects will reside at the Phase I clinical site from Days -1 to 3 (48 hours postdose). Subjects will return to the clinical site for outpatient visits on Days 4 and 5. There will be at least a 10-day washout between doses (from Period 1, Day 1 to Period 2, Day 1).

Six subjects will be randomised to receive 25 mg KL1333 and 2 subjects will be randomised to receive placebo, and subjects will receive the same treatment in both treatment periods. On Treatment Period 1, Day 1, subjects will receive a single oral dose of study drug following an overnight fast of at least 8 hours. On Treatment Period 2, Day 1, subjects will receive a single oral dose of study drug after consuming a standard high-fat breakfast. Following review of safety, tolerability, and PK data, up to 2 additional dose cohorts of healthy subjects may be added if needed to determine the study treatment for Part B. Additional single-dose cohorts may be enrolled based on data obtained from either Parts A or B. If additional cohorts are required, each cohort will consist of 8 subjects, with 6 subjects receiving KL1333 and 2 subjects receiving placebo, and will undergo a single treatment period. The dose level and dietary state for administration of KL1333 in these potential additional cohorts will be decided following review of data in Part A and any available data from Part B, and the dose level could be either less than or greater than 25 mg. The dose level will not exceed 600 mg,

and the predicted exposure following a single dose in any subject in Part A will not exceed an $AUC_{0\text{-}24}$ of 51,800 ng.h/mL for derived total KL1333.

Subjects will return for a Follow-up visit on Day 6, 5 days after their final dose.

An overview of the study design is shown in Figure 2.

Figure 2: Study Schematic (Cohort A1, Fasted versus Fed [High-fat Breakfast] and Additional Optional Cohorts A2 and A3)

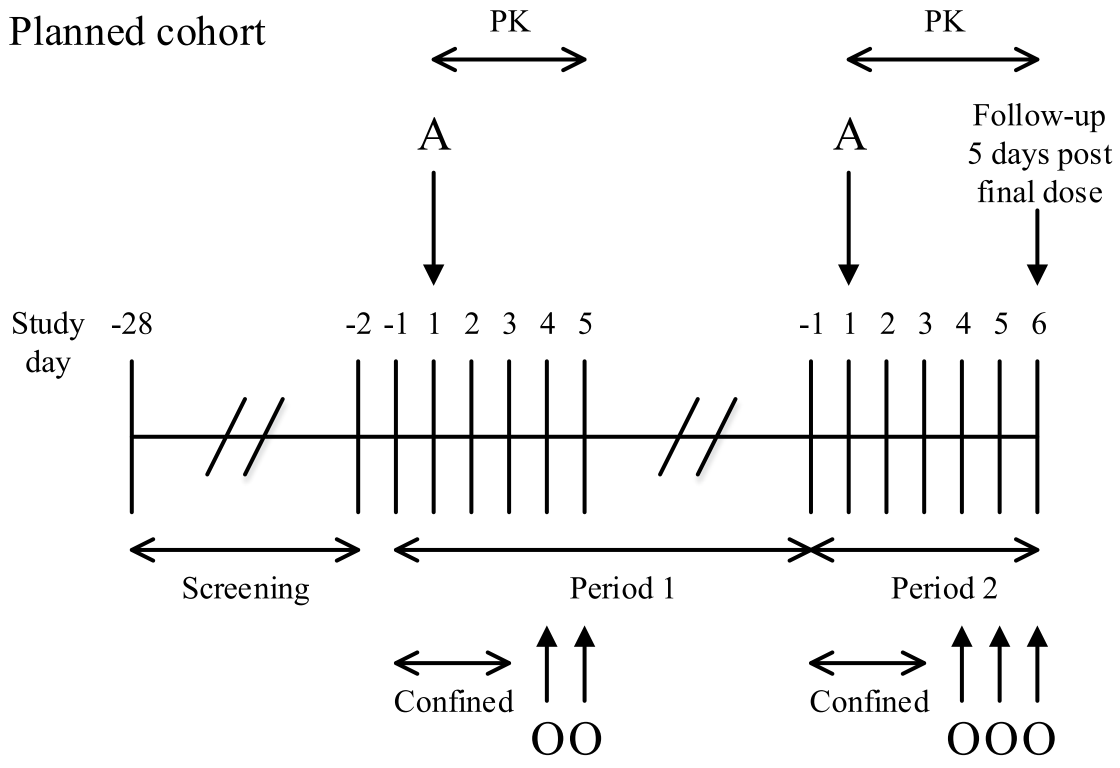

A = KL1333 administration; O = outpatient visit; PK = pharmacokinetics.

In Treatment Period 1, dosing will occur after an overnight fast of at least 8 hours. In Treatment Period 2, dosing will occur 30 minutes after starting a high-fat breakfast. The washout will be at least 10 days between doses (from Period 1, Day 1 to Period 2, Day 1).

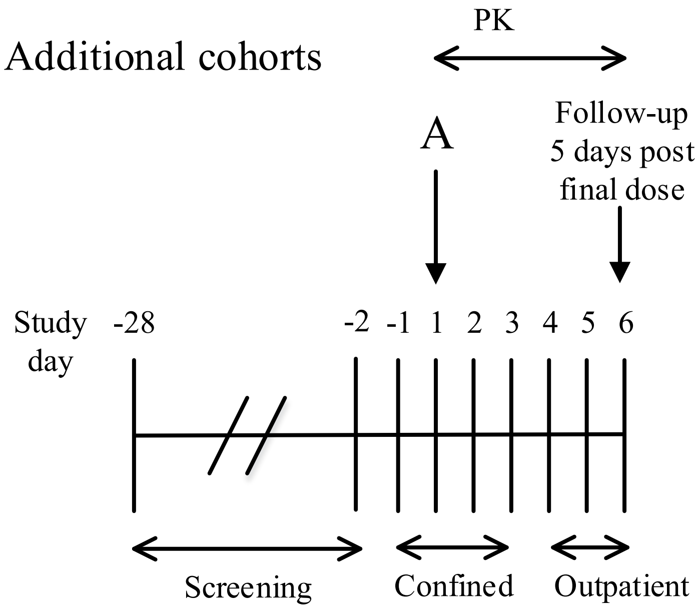

A = KL1333 administration; PK = pharmacokinetics.

If additional cohorts are required, dietary state will be decided following review of data in Part A and available data from Part B.

The total duration of study participation for each subject (from Screening through Follow-up visit) is anticipated to be approximately 6 weeks for Cohort A1 and approximately 5 weeks for additional optional Cohorts A2 and A3.

3.1.2. Part B

Part B will comprise a randomised, multiple-dose, sequential-group, placebo-controlled study. Sixteen healthy subjects will be studied in 2 cohorts (Groups B1 and B2), with each cohort consisting of 8 subjects. Part B may start after completion of Group A1, at a dose equal to or less than given in Part A.

Potential subjects will be screened to assess their eligibility to enter the study within 28 days prior to the first dose administration. All subjects will participate in 1 treatment period and will reside at the Phase I clinical site from Days -1 to 12 (48 hours post final dose). Subjects will return to the clinical site for outpatient visits on Days 13 and 14.

On Day 1, 6 subjects will be randomised to receive KL1333 and 2 subjects will be randomised to receive placebo. The preliminary planned doses of KL1333 for Groups B1 and B2 are 25 and 50 mg, respectively, once daily (QD) on Days 1 to 10. Dose levels, dose frequency, and dietary state will be confirmed following review of safety, tolerability, and PK data from Part A and ongoing data from Part B. Additionally, a dose selection conference meeting will be held before each cohort in Part B where blinded data from the previous cohort will be reviewed before a decision is made about proceeding to the next cohort. Following review of safety, tolerability, and PK data, up to 3 additional dose cohorts of

healthy subjects may be added to further explore the PK, safety, and tolerability of KL1333. If additional cohorts are required, each cohort will consist of 8 subjects, with 6 subjects receiving KL1333 and 2 subjects receiving placebo. The dose level will not exceed 600 mg, and the predicted exposure following multiple daily dose administration in any subject in Part B will not exceed an $AUC_{0\text{-}24}$ of 51,800 ng.h/mL for derived total KL1333. There will be a minimum of 6 days between dose escalations for each cohort (between the last dose of one cohort and the first dose of the next cohort).

Subjects will return for a Follow-up visit on Day 15, 5 days after their final dose.

An overview of the study design is shown in Figure 3.

Figure 3: Study Schematic (Part B)

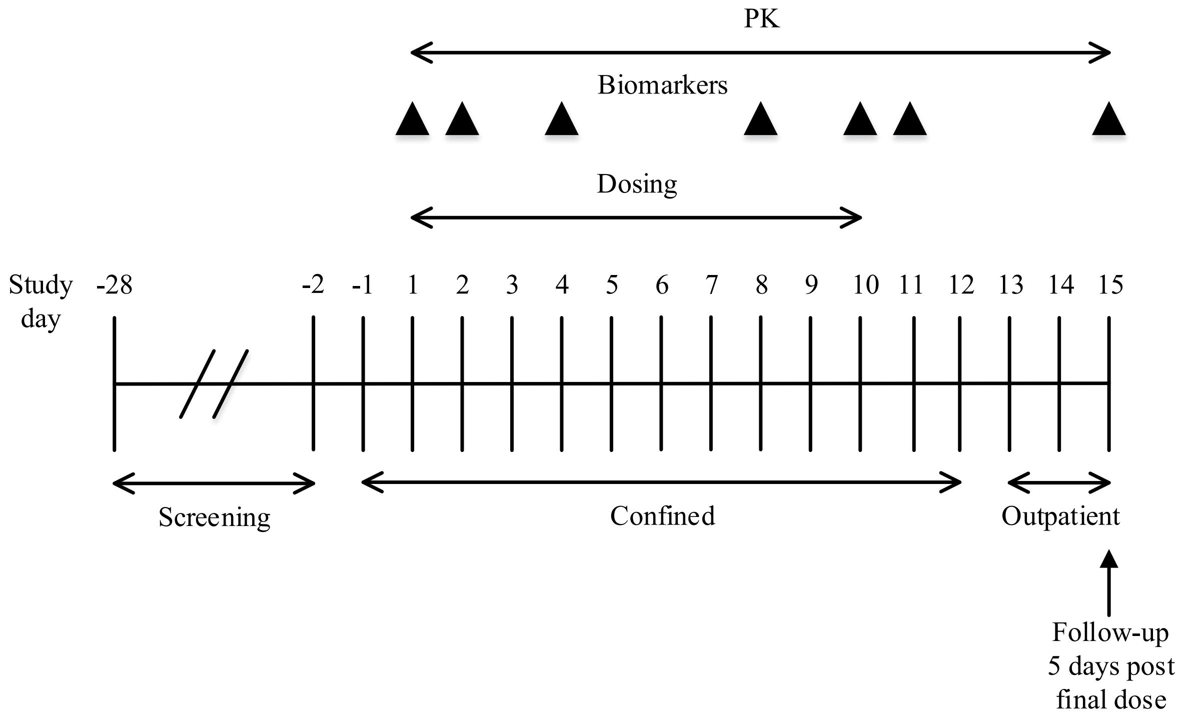

▲ = biomarker sample; PK = pharmacokinetics.

Dietary state will be confirmed from review of data from Part A and available data from Part B.

The total duration of study participation for each subject (from Screening through Follow-up visit) is anticipated to be approximately 6 weeks for Part B.

3.1.3. Part C

Part C will comprise a randomised, multiple-dose, single-group, placebo-controlled study. A total of 8 patients diagnosed with any mitochondrial disease will be enrolled in this part of the study. Part C may start after the dose selection conference has been completed for the final cohort of Part B, at a daily dose no higher than the highest well-tolerated dose in Part B.

Potential study patients will be screened to assess their eligibility to enter the study within 75 days prior to the first dose administration. Patients will reside at the clinical site, or nearby the clinical site at a hotel recommended by the clinical site, from Days -1 to 2 and Days 10 to 11. Patients will return to the clinical site for outpatient visits on Days 4 and 8. Patients will be randomised on Day 1.

Two patients will be dosed initially, with 1 patient receiving KL1333 and 1 patient receiving placebo. If there are no safety or tolerability concerns in these patients following the Day 4 visit, the remaining 6 patients, with 5 patients receiving KL1333 and 1 patient receiving placebo, will be enrolled on a rolling basis. In the event there are safety concerns following completion of the 2 sentinel patients without meeting stopping criteria, the Sponsor may add intermediate cohorts. The dosing schedule of intermediate cohorts will be the same as that of the planned cohort. The schedule of safety assessments of intermediate cohorts will be the same as that of the planned cohort as a general rule. Whether or not to add safety assessments in the intermediate cohorts will be determined by the Sponsor.

It is planned for patients to receive study drug QD on Days 1 to 10. Dose levels, dose frequency, and dietary state will be confirmed following review of safety, tolerability, and PK data from Part B, and unless deemed very unfavourable for the conduct of the study, the patients will not be required to be fasting prior to dosing. Study drug will be administered by clinical site staff when the patients are resident at the clinical site or return for outpatient visits. On all other days, patients will record drug administration and any concomitant medications in a diary that will be provided to each patient. Diaries will be reviewed and checked for compliance during the outpatient visits and as part of the check-in procedures on Day 10. Clinical symptoms that occur while patients are not resident at the site will be collected by the site using standard AE reporting procedures.

Patients will return for a Follow-up visit on Day 15, 5 days after their final dose.

An overview of the study design is shown in Figure 4.

Figure 4: Study Schematic (Part C)

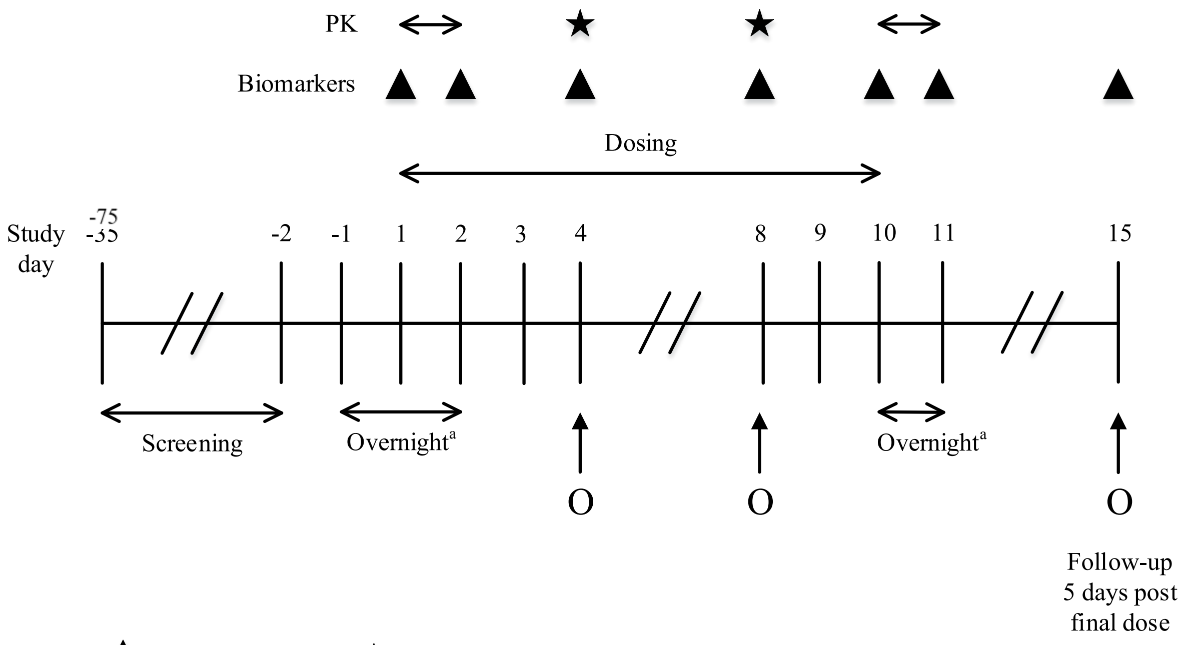

▲= biomarker sample; ★= pharmacokinetic sample; O = outpatient visit;
PK = pharmacokinetics.

Dietary state will be confirmed from review of data from Part B and unless deemed very unfavourable for the conduct of the study, the patients will not be required to be fasting prior to dosing.

[a] Patients can reside at the clinical site or nearby the clinical site at a hotel.

The total duration of study participation for each patient (from Screening through Follow-up visit) is anticipated to be approximately 7-11 weeks for Part C.

3.1.4. Part D

Part D will comprise a randomised, multiple-dose, placebo-controlled study. Sixteen healthy subjects will be studied in 2 cohorts (Groups D1 and D2), with each cohort consisting of 8 subjects. Part D will start after completion of Part B, and the Part D groups may be run in parallel.

Potential subjects will be screened to assess their eligibility to enter the study within 35 days prior to the first dose administration. All subjects will participate in 1 treatment period and will reside at the Phase I clinical site from Days -1 to 12 (48 hours post final dose). Subjects will return to the clinical site for outpatient visits on Days 13 and 14.

On Day 1, 6 subjects will be randomised to receive KL1333 and 2 subjects will be randomised to receive placebo in each group. The doses of KL1333 for Groups D1 and D2 are 75 mg twice daily (BID) and 50 mg 3 times daily (TID), respectively, on Days 1 to 10 with a single dose administration on Day 10. The first dose on Day 1 and the dose on Day 10 will be administered following an overnight fast of at least 8 hours due to intensive PK sampling. The first dose on Day 7 will be administered following an overnight fast of at least 8 hours due to clinical laboratory evaluations.

Subjects will return for a Follow-up visit on Day 15, 5 days after their final dose.

An overview of the study design is shown in Figure 5.

Figure 5: Study Schematic (Part D)

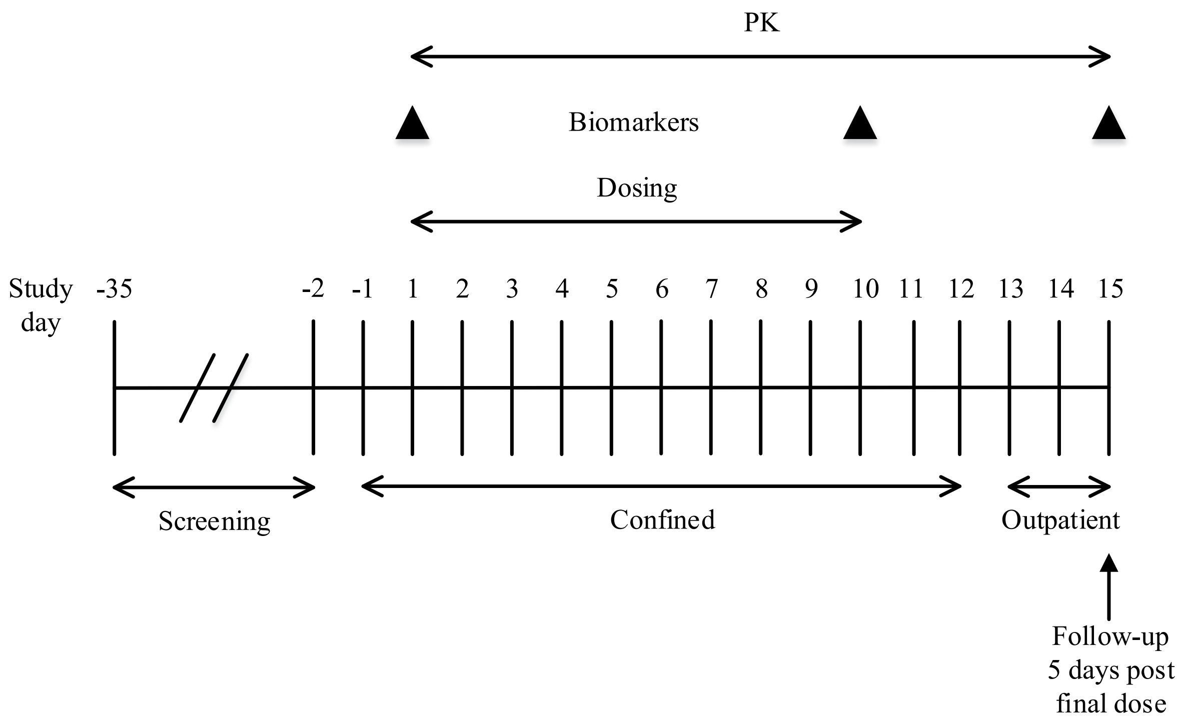

▲ = biomarker sample; PK = pharmacokinetics.

Dosing will be twice daily for Group D1 and 3 times daily for Group D2. There will be a single dose administration on Day 10.
Subjects will be fasted for the first dose on Day 1 and single dose on Day 10 for intensive pharmacokinetic sampling and the first dose on Day 7 for clinical laboratory evaluations.
All other doses may be given without regard to food.

The total duration of study participation for each subject (from Screening through Follow-up visit) is anticipated to be approximately 7 weeks for Part D.

Schedules of Assessments are presented in Appendix 5.

3.2. Study Start and End of Study Definitions

The start of the study is defined as the date the first subsequently enrolled subject signs an Informed Consent Form (ICF). The point of enrolment occurs at the time of subject number allocation. The end of the study is defined as the date of the last subject's last study-specific assessment visit (scheduled or unscheduled).

3.3. Additional Groups

Following review of the safety, tolerability, and PK data, additional dose cohorts (not to exceed the exposure limits in Section 3.7) may be added to the study. Up to 2 further cohorts of 8 subjects (6 active:2 placebo) may be included in Part A and up to 3 further cohorts of 8 subjects (6 active:2 placebo) may be included in Part B. The requirement for additional cohorts will be agreed with the Sponsor.

3.4. Discussion of Study Design, Including the Choice of Control Groups

As KL1333 has previously been given as single ascending doses to Korean subjects in Study KL1333_101, Part A is planned to have 1 single-dose cohort to compare single-dose PK data in non-Korean subjects before administering multiple doses to non-Korean subjects. A crossover design has been chosen to study the food effect, as this gives a within-subject assessment of the influence of food on the PK of KL1333 and so increases the power of the study for the given number of subjects. For Part B, a sequential-group, ascending-dose design has been chosen for safety reasons as KL1333 is in the early stages of clinical development, and this will be the first time it will be administered in multiple doses to humans. Oral doses have been chosen for this study, as this is the intended clinical route of administration and based on the results of the single ascending dose study.

It is the intent of Part B to dose subjects such that steady-state plasma levels of KL1333 are achieved and maintained for several days. Based on the available single ascending dose data, it is expected that this will be achieved following 10 days of once-daily dosing; additionally, a full review of all the safety, tolerability, and PK data from Part A will be performed to confirm the dose regimen for Part B.

Based upon the nonclinical data and the data from the single ascending dose study, the duration of each treatment period is considered adequate to achieve the study objectives. An interval of at least 10 days between treatment periods in Part A is considered adequate to prevent carryover of KL1333, given the observed mean half-life for free KL1333 of 13.73 to 34.64 hours in the single ascending dose study.

This study will be double-blind and placebo-controlled in order to avoid bias in the collection and evaluation of data during its conduct. Placebo has been chosen as the control treatment to assess whether any observed effects are treatment related or simply reflect the study conditions. The safety and PK assessments are standard parameters for clinical studies in drug development. Based on the PK data from Study KL1333_101, the plasma sampling timepoints are expected to sufficiently estimate PK parameters of KL1333 exposure.

Conducting Parts A, B, and D in healthy subjects mitigates the potential confounding effects of the disease state and concomitant medications. Conducting Part C in patients with mitochondrial disease allows for a preliminary assessment of KL1333 in patients, including a preliminary exploration of the selected clinical assessments (Section 7.2.3).

Blood samples will be taken to assess the effect of multiple doses of KL1333 on the selected biomarkers. Blood samples will also be taken for possible later analysis of metabolomics. Continuous 12-lead ECG monitoring will be included in the healthy subjects in this study to collect data that will allow for future investigation of potential KL1333 effects on the QT

interval, with a view of supporting a potential thorough QT study waiver. Blood samples will be taken in case unexpected variability is seen in the PK data that would warrant NQO1 genotyping as there is a variant allele of this enzyme that is associated with a reduction in NQO1 protein and activity that has been shown to be more common in the Korean population than the non-Hispanic white population.[2]

As a result of gastrointestinal AEs seen to date in this study, supplementary safety assessments for C-reactive protein (CRP), erythrocyte sedimentation rate (ESR), and faecal calprotectin will be assessed in Group B5 and Part D healthy subjects and if required/indicated in Part C patients to better evaluate the gastrointestinal AEs by exploring potential levels of general and gastrointestinal tract inflammation.

Part D was added to this study to investigate if splitting the KL1333 dose throughout the day will improve safety and tolerability and increase exposure. This part will include one 75 mg KL1333 BID cohort and one 50 mg KL1333 TID cohort to compare to the 150-mg QD cohort in Part B. To obtain further PD information in Part D, postdose blood samples for biomarkers and metabolomics will be collected and whole blood metabolomics will be evaluated in addition to the serum metabolomics that was performed in Parts A through C. The 2 dose cohorts in Part D may be run in parallel as Part B was completed prior to the addition of Part D to the protocol.

3.4.1. Dose Interval

As KL1333 has already been administered as single doses to Korean subjects up to 800 mg in Study KL1333_101, all subjects in a given dose cohort in Parts A and B of this study can be dosed on the same day.

Dosing in Part C will be such that 2 patients (1 KL1333 and 1 placebo) will be dosed initially as an extra precaution given the fragile patient population. If there are no safety or tolerability concerns per the Investigator in these patients following the Day 4 visit, the remaining 6 patients, with 5 patients receiving KL1333 and 1 patient receiving placebo, will be enrolled on a rolling basis.

All subjects in a given dose cohort in Part D of this study can be dosed on the same day; doses of up to 250 mg KL1333 QD in Part B were shown to be safe and moderately well tolerated (no dose escalation stopping criteria were met), although frequent gastrointestinal AEs were reported prior to the addition of Part D.

3.5. Selection of Doses in the Study

The NOAEL was 120 mg/kg in a 4-week repeat-dose toxicology study in the rat and 40 mg/kg in a 13-week repeat-dose toxicology study in the dog. In the 13-week repeat-dose rat study, the NOAEL was 80 mg/kg/day in males and was not determined (ie, <80 mg/kg/day) in females. The human equivalent dose of 40 mg/kg in dog is 22.2 mg/kg or a dose of >1000 mg in a 60-kg subject. Using the previous bioanalytical methods, the free KL1333 exposure in the dog was C_{max} of 31.491 and 15.886 ng/mL in males and females, respectively, and AUC_{0-24} of 132.880 and 120.280 ng.h/mL in males and females, respectively. The total KL1333 exposure was C_{max} of 1365.7 and 799.5 ng/mL in males and

females, respectively, and AUC_{0-24} of 5335.2 and 3003.3 ng.h/mL in males and females, respectively.

The previous bioanalytical methods may have underestimated the total KL1333 concentration as the de-conjugation of the sulphate metabolites has been shown to achieve only approximately 50% conversion in the sample work-up. The new bioanalytical method is expected to report higher values. Therefore, the safety margins based on the total concentration of KL1333 and conjugated metabolites in the dog or rat may be conservative.

KL1333 was safe and well tolerated when given as single doses up to 600 mg in Korean subjects in Study KL1333_101. The proposed IMP dose levels for Parts A and B are shown in Table 4.

Table 4: Proposed Investigational Medicinal Product Dose Levels for Parts A and B

Study Part	Cohort	Subject Numbers	Treatment Period 1	Treatment Period 2
A	A1	101 – 108	25 mg or placebo (fasted)	25 mg or placebo (fed)
B	B1	201 – 208	Selected from Part A, possibly 25 mg or placebo	
	B2	209 – 216	Selected from Part A and Cohort B1, possibly 50 mg or placebo	

The planned starting doses for Parts A and B are lower than the highest well-tolerated single dose administered to Korean subjects. A starting dose of 25 mg has been selected for this study, as this is expected to have quantifiable PK regardless of any potential change in exposure due to the use of non-Korean subjects, dosing after a high-fat breakfast, or use of a revised bioanalytical method. For subsequent cohorts, dose levels, dosing frequency, and dietary state will be decided, in consultation with the Sponsor, on the basis of available exposure and safety data from the previous cohorts and the predicted accumulation. The dose level in Parts A and B will not exceed 600 mg, and the total daily dose of KL1333 administered during Part C of the study will not exceed the maximum well-tolerated dose level studied in Part B.

A maximum exposure limit was selected based on nonclinical genotoxicity studies. KL1333 did not induce mutations in bacteria in an Ames test or induce MN in rat bone marrow in vivo when tested up to the maximum tolerated dose. However, it did induce chromosomal aberrations in cultured human lymphocytes, both in the absence and presence of metabolic activation, and did induce DNA strand breakage (comets) in both duodenum and liver of rat in vivo. The DNA damage in duodenum was considered most likely to be an indirect consequence of local toxicity and an inflammatory response. However, the DNA damage in liver could be due to extreme pharmacological effects – the action of NQO1 on KL1333 leading to redox cycling and generation of ROS.

Further investigations in vitro demonstrated that cytotoxicity and DNA damage were caused by ROS and could be neutralised by effective scavenging. Thus, in cells and tissues where sufficient antioxidant defences exist, no DNA damage would be induced, but when antioxidant defences are saturated (such as high doses in the in vivo comet assay in rats)

DNA damage will occur. Thus, the mode of action of KL1333-induced genotoxicity indicates a threshold.

In light of the positive comet assay results in rat liver, but with evidence of this being due to oxidative damage and exhibiting a nonlinear dose response, it was possible that the DNA strand breaks may either be repaired or be lethal, and not necessarily lead to stable genetic changes. Therefore, a repeat-dose liver MN study in rats was performed with KL1333 doses up to 200 mg/kg/day. With the data from this study, the risks can be re-evaluated as follows:

- Since no MN were induced, it is clear that the DNA strand breaks induced in the comet assay did not convert into biologically relevant genotoxic changes.
- No genotoxicity was induced after repeated dosing, confirming that accumulation of KL1333 was not any more hazardous than acute dosing.
- The liver MN assay investigated a more biologically relevant endpoint than the comet assay.

The mean AUC_{0-24} at the maximum tested and nongenotoxic dose of 200 mg/kg/day was 259,000 ng.hr/mL using the revised bioanalytical method that measures KL1333 as the sum of parent KL1333, de-conjugated glucuronidated KL1333 metabolites, and sulphated KL1333 metabolites. Therefore, the derived total KL1333 plasma exposure limit in this study will be an AUC_{0-24} of 51,800 ng.hr/mL (ie, 5× lower than the 259,000 ng.hr/mL obtained at 200 mg/kg/day in the liver MN assay).

Dose levels should not increase by more than 3.0-fold between dose cohorts. The highest dose level may exceed the top dose shown in Table 4, providing systemic exposure does not exceed that stated in the dose escalation stopping criteria (Section 3.7). The selected dose levels for each cohort will be documented in the dose escalation meeting minutes. Details of all doses administered in this study will be documented in the electronic Case Report Form (eCRF).

In Part D, KL1333 doses of 75 mg BID and 50 mg TID were chosen to enable comparison to the 150-mg QD dose in Group B3, as all 3 groups will receive a total daily dose of 150 mg KL1333. A 150-mg total daily dose was selected as it was moderately well tolerated in Group B3, with some mild to moderate gastrointestinal TEAEs, including nausea, loose stools, or abdominal pain/stomach cramps in 4 of the subjects. Part D will allow testing of the hypothesis that BID or TID dosing improves tolerability, resulting in a decrease in TEAE incidence. Exposure in Part D will also be evaluated to determine if splitting the dose throughout the day increases the exposure. The doses selected in Part D are still anticipated to be below the maximum exposure limits defined by the protocol.

3.6. Dose Escalation

In Part B, doses will be administered in an escalating manner following satisfactory review by the Sponsor and Phase I healthy subject clinical site Investigator of the safety, tolerability, and PK data from the lower dose levels (including Part A). The review will include safety and tolerability data up to 48 hours postdose, as this covers approximately 2 times the anticipated $t_{1/2}$ and most AEs in Study KL1333_101 were reported within 48 hours postdose, and PK data up to 5 days postdose, as this is approximately 5 times the anticipated $t_{1/2}$. There

will be a minimum of 6 days between dose escalations (between the last dose of one cohort and the first dose of the next cohort) to allow sufficient time for analysis of KL1333 PK samples through 5 days postdose and an adequate safety review.

Doses may be reduced and may be lower than the planned doses.

Dose escalation in Part B will only occur if data from a minimum of 6 subjects have been reviewed from the previous lower dose cohort, such that data from a minimum of 4 subjects who have received KL1333 will be used to make the dose escalation decision.

The justification for this is as follows:

- Based upon nonclinical data, no clinically important off-target effects are expected within the proposed dose range.
- A minimum of 4 subjects receiving the active drug is considered sufficient to characterise the safety profile and PK response to KL1333.

Between each dose escalation, the Phase I healthy subject clinical site Investigator will review all available blinded data to ensure it is safe to proceed with the planned dose escalation. An interim safety report, summarising results from all available safety assessments, will be sent to the Sponsor prior to the start of each successive cohort. Any clinically significant results will be discussed with the Sponsor before dose escalation continues. Interim PK data will also be reviewed in terms of dose escalation and to confirm that the study design remains appropriate. In the event of a disagreement between the Sponsor and Phase I healthy subject clinical site Investigator on the dose escalation decision, the decision of the Investigator will be upheld.

Supplementary safety assessments for CRP, ESR, and faecal calprotectin are planned for Group B5 and optional for Part C. At the final dose selection conference for Part B, timepoints for these supplementary safety assessments in Part C may be adjusted, added, or removed following review of safety, tolerability, and PK data from Part B.

3.7. Dose Escalation Stopping Criteria

The dose level for this study will not exceed 600 mg. In addition, dosing will be stopped and dose progression will be halted until safety information can be reviewed in the event that:

- One or more subjects experience a serious AE (SAE) that is considered to be related to IMP or 2 or more subjects experience severe AEs that are considered to be related to IMP
- There is evidence of clinically significant increases in liver function tests (aspartate aminotransferase, alanine aminotransferase, alkaline phosphatase, bilirubin, and/or gamma-glutamyl transferase), defined as 3 times the upper limit of normal in 3 or more subjects in a cohort compared to Day -1 measurements (confirmed with repeat testing)
- QTcF increases >60 msec compared to baseline and/or absolute QTcF values >500 msec (confirmed by single repeat measurement) in 2 or more subjects in a cohort

- Severe diarrhoea, defined as 7 or more episodes, rated as ≥5 on the Bristol Stool Chart, in 1 day for 2 consecutive days in 2 or more subjects in a cohort
- The exposure for any subject in the planned dosing cohort is predicted to exceed an AUC_{0-24} of 51,800 ng.h/mL for derived total KL1333 (as the sum of parent KL1333, de-conjugated glucuronidated KL1333 metabolites, and sulphated KL1333 metabolites). For Part D, AUC_{0-24} will be determined as $AUC_{0-\tau} \times 2$ for BID and $AUC_{0-\tau} \times 3$ for TID regimens.

If, following an internal safety review, the Sponsor deems it appropriate to restart the study, this can be done following approval of a substantial protocol amendment.

4. SELECTION OF STUDY POPULATION

4.1. Inclusion Criteria

Healthy subjects and patients with mitochondrial disease must satisfy all of the following criteria at the Screening visit unless otherwise stated:

1. Females will not be pregnant or lactating, and females of childbearing potential and males will agree to use contraception as detailed in Appendix 3.
2. Able to comprehend and willing to sign an ICF and to abide by the study restrictions.
3. Able to perform all protocol-specified assessments and comply with the study visit schedule.

Additional inclusion criteria for healthy subjects:

4. Males or females, of any race, between 18 and 65 years of age, inclusive.
5. Weight ≥50 kg and body mass index between 18.0 and 32.0 kg/m^2, inclusive.
6. In good health, determined by no clinically significant findings from medical history, physical examination, 12-lead ECG, vital signs measurements, and clinical laboratory evaluations (congenital nonhaemolytic hyperbilirubinaemia [eg, Gilbert's syndrome] is not acceptable) at Screening and Check-in as assessed by the Investigator.

Additional inclusion criteria for patients with mitochondrial disease:

7. Males or females, of any race, between 18 and 75 years of age, inclusive.
8. Body mass index between 15.0 and 32.0 kg/m^2, inclusive.
9. Any mitochondrial disease that has been genetically confirmed.
10. Clinically stable, apart from symptoms associated with the diagnosis of mitochondrial disease, as determined by medical history, physical examination, 12-lead ECG, vital signs measurements, and clinical laboratory evaluations at Screening and Check-in as assessed by the Investigator.
11. Willing to suspend treatment with idebenone and medications (prescription or nonprescription) that have effects on metabolism or unknown binding sites (eg, vitamin E, co-enzyme 10, arginine) during the Screening and study periods.

4.2. Exclusion Criteria

Healthy subjects and patients with mitochondrial disease will be excluded from the study if they satisfy any of the following criteria at the Screening visit unless otherwise stated:

1. History of significant hypersensitivity, intolerance, or allergy to any drug compound, food, or other substance, including KL1333 or its excipients, unless approved by the Investigator.
2. History of gastroesophageal reflux disease, gastric erosions, peptic ulcer disease, or gastrointestinal bleeding episodes.
3. History of stomach or intestinal surgery or resection that would potentially alter absorption and/or excretion of orally administered drugs including cholecystectomy (uncomplicated appendectomy and hernia repair will be allowed).
4. History of malignancy of any organ system other than localised basal cell carcinoma of the skin, treated or untreated, within 5 years prior to Screening, regardless of whether there is evidence of local recurrence or metastases.
5. History of clinically significant illness (except for mitochondrial disease in the patients in Part C) or surgery within 4 weeks prior to Screening, as determined by the Investigator.
6. History of alcoholism or drug/chemical abuse within 2 years prior to Screening.
7. Alcohol consumption of >28 units per week for males and >21 units per week for females. One unit of alcohol equals ½ pint (285 mL) of beer or lager, 1 glass (125 mL) of wine, or 1/6 gill (25 mL) of spirits.
8. Positive alcohol breath test result or positive urine drug screen (confirmed by repeat) at Screening or Check-in.
9. Positive hepatitis panel and/or positive human immunodeficiency virus test (Appendix 2).
10. Haemoglobin or haematocrit below the lower limits of normal, deemed clinically significant by the Investigator.
11. Participation in a clinical study involving administration of an investigational drug[1] (new chemical entity) within 90 days or 5 half-lives, whichever is longer, prior to the first dose, or concomitant participation in an investigational study involving no drug administration.

[1] *For patients with mitochondrial disease: administration of temporary authorized (and authorized) SARS-CoV-2 virus vaccines, even if technically considered investigational drugs, is allowed unless the patient is participating in another clinical study.*

12. Use or intend to use any medications/products known to alter drug absorption, metabolism, or elimination processes, including St. John's wort, within 30 days prior to the first dose, unless deemed acceptable by the Investigator.
13. Receipt of blood products within 2 months prior to Screening.
14. Donation of blood from 3 months prior to Screening, plasma from 2 weeks prior to Screening, or platelets from 6 weeks prior to Screening.
15. Poor peripheral venous access that precludes collection of blood samples.
16. Have previously completed or withdrawn from this study and have previously received the IMP. Subjects who received IMP in Part A will not be allowed to participate in Part B or Part D.
17. Member of the study personnel or their immediate families, or is a subordinate or immediate family member of a subordinate to any of the study personnel.
18. Has a disease (except for mitochondrial disease in the patients in Part C) or takes medication that could interfere with the conduct or interpretation of the study, in the opinion of the Investigator.
19. Should not participate in this study or is unlikely to comply with the protocol, in the opinion of the Investigator.

Additional exclusion criteria for healthy subjects:

20. Dietary restriction that would prevent the subject from consuming the high-fat breakfast (Table 5).
21. Significant history or clinical manifestation of any metabolic, allergic, dermatological, hepatic, renal, haematological, pulmonary, cardiovascular, gastrointestinal, neurological, respiratory, endocrine, or psychiatric disorder, as determined by the Investigator.
22. Any of the following vital signs abnormalities. A single repeat is allowed for eligibility based on the Investigator's judgment:
    - Systolic blood pressure <90 mmHg or >160 mmHg
    - Diastolic blood pressure <50 mmHg or >90 mmHg
    - Pulse rate <40 bpm or >100 bpm.
23. Any of the following ECG abnormalities. A single repeat testing is allowed for eligibility based on the Investigator's judgment:
    - Abnormal ECG that is clinically significant, as determined by the Investigator
    - PR interval >250 msec
    - QRS interval >130 msec
    - QTcF >450 msec (for males) or >470 msec (for females).
24. Use or intend to use any prescription medications/products within 14 days prior to the first dose, unless deemed acceptable by the Investigator.

25. Use or intend to use slow-release medications/products considered to still be active within 14 days prior to the first dose, unless deemed acceptable by the Investigator.
26. Use or intend to use any nonprescription medications/products including vitamins, minerals, and phytotherapeutic/herbal/plant-derived preparations within 7 days prior to the first dose, unless deemed acceptable by the Investigator.
27. Use of tobacco- or nicotine-containing products within 3 months prior to Screening.

Additional exclusion criteria for patients with mitochondrial disease:

28. Any of the following ECG abnormalities. Triplicate repeat testing is allowed for eligibility based on the Investigator's judgment:
    - Abnormal ECG that is clinically significant, as determined by the Investigator
    - QTcF >500 msec.
29. Patient requires antiepileptic medication that might have an effect on CYP or glucuronyl transferase enzymes (eg, phenytoin, carbamazepine, and valproic acid).
30. Patient requires medication that is metabolized by the following CYPs and the Investigator and Medical Monitor, in consultation with the Sponsor, consider there to be a potential risk for a clinically significant drug interaction:
    - CYP1A2 (eg, clozapine, fluvoxamine, haloperidol, imipramine, nabumetone, naproxen, olanzapine, theophylline, tizanidine, and zolmitriptan)
    - CYP2B (eg, temazepam, diazepam, selegiline, mephobarbital, and mexiletine)
    - CYP3A4 (eg, clarithromycin, erythromycin, quinidine, alprazolam, diazepam→3OH, midazolam, triazolam, cyclosporine, tacrolimus [FK506], sirolimus, astemizole, chlorpheniramine, diltiazem, verapamil, atorvastatin, lovastatin, simvastatin, sildenafil, tadalafil, vardenafil, quinine, alprazolam, ketoconazole, itraconazole, and zolpidem).
31. Use of idebenone or medications (prescription or nonprescription) that have effects on metabolism or unknown binding sites (eg, vitamin E, co-enzyme 10, arginine) within 7 days or 5 half-lives, whichever is longer, prior to the first dose.
32. Use of prescription drugs within 14 days prior to dosing, with the exception of hormone replacement therapy; oral, implantable, transdermal, injectable, or intrauterine hormonal contraceptives; and established therapy for mitochondrial disease and the treatment of associated disorders that has been stable for at least 7 days prior to the first dose, as approved by the Medical Monitor and Investigator, in consultation with the Sponsor.
33. Uncontrolled diabetes mellitus, as determined by the Investigator.
34. Creatinine clearance <45 mL/min as calculated by the Cockcroft-Gault equation.
35. Patient requires a pacemaker or defibrillator or has undergone cardiac surgery within 2 years prior to Screening.

4.3. Subject Number and Identification

Subjects will have a unique identification number used at Screening. Subjects will be assigned a subject number at the time of their randomisation. Assignment of subject numbers will be in ascending order and no numbers will be omitted (eg, in Part A: Subjects 101, 102; in Part B: Subjects 201, 202; in Part C: Patients 301, 302; in Part D: Subjects 401, 402). Sequential numbering may restart for each participating site as the subject identification number will ultimately be comprised of the site number and subject number (eg, Subject 001-101). Replacement subjects (Section 4.4) will be assigned a subject number corresponding to the number of the subject he/she is replacing plus 1000 (eg, Subject 1101 replaces Subject 101).

Subjects will be identified by subject number only on all study documentation. A list identifying the subjects by subject number will be kept in the Site Master File.

4.4. Subject Withdrawal and Replacement

A subject is free to withdraw from the study at any time. In addition, a subject will be withdrawn if any of the following criteria are met:

- change in compliance with any inclusion/exclusion criterion that is clinically relevant and affects subject safety as determined by the Investigator (or designee)
- noncompliance with the study restrictions that might affect subject safety or study assessments/objectives, as considered applicable by the Investigator (or designee)
- any clinically relevant sign or symptom that, in the opinion of the Investigator (or designee), warrants subject withdrawal
- for Part C, QTcF increases >60 msec compared to baseline and/or absolute QTcF values >500 msec (confirmed by repeat triplicate measurement).

If a subject is withdrawn, the Sponsor will be notified and the date and reason(s) for the withdrawal will be documented in the subject's eCRF. If a subject is withdrawn, efforts will be made to perform all follow-up assessments, if possible (Appendix 5). Other procedures may be performed at the Investigator's (or designee's) and/or Sponsor's discretion. If the subject is in-house, these procedures should be performed before the subject is discharged from the clinic. The Investigator (or designee) may also request that the subject return for an additional Follow-up visit. All withdrawn subjects will be followed until resolution of all their AEs or until the unresolved AEs are judged by the Investigator (or designee) to have stabilised.

Subjects who are withdrawn for reasons not related to study drug may be replaced following discussion between the Investigator and the Sponsor. Subjects withdrawn as a result of AEs thought to be related to the study drug will generally not be replaced.

4.5. Study Termination

The study may be discontinued at the discretion of the Phase I healthy subject clinical site Investigator or the Sponsor. The reasons for such action may include, but are not limited to:

- AEs unknown to date (ie, not previously reported in any similar investigational study drug trial with respect to their nature, severity, and/or duration)
- increased frequency, severity, and/or duration of known, anticipated, or previously reported AEs (this may also apply to AEs defined at Check-in as baseline signs and symptoms)
- medical or ethical reasons affecting the continued performance of the study
- difficulties in the recruitment of subjects
- cancelation of drug development.

5. STUDY TREATMENTS

5.1. Description, Storage, Packaging, and Labelling

The IMPs (25 and 100 mg KL1333 tablet and matching placebo) will be supplied by the Sponsor (or designee), along with the batch numbers and Certificates of Analysis. A Certificate of Release authorised by a Qualified Person in the European Union will also be issued for the IMP. The IMP will be provided in high-density polyethylene bottles and stored according to the instructions on the label.

All IMPs will be stored at the study sites in a location that is locked with restricted access.

The bulk drug container and unit dose containers will be labelled in accordance with national laws and regulations. The IMPs will be transferred from bulk supplies into the subject's dose container by qualified staff.

5.2. Study Treatment Administration

When subjects are at a clinical site at the time of dosing, doses of KL1333 and placebo will be administered orally with approximately 240 mL of room temperature water. Timing of meals relative to drug administration is detailed in Section 6.2.

In Part C when patients are nonresident from the clinical sites and self-administer study drug, water can be consumed ad libitum with the dose. Patients will record drug administration in a diary that will be provided to them. Patients will bring the diary with them when returning to the clinical site. Diaries will be reviewed and checked for compliance at each visit and as part of the check-in procedures for clinical site stays, and the dosing information will be recorded in the eCRF.

For doses administered in the clinical site, subjects will be administered the IMP in numerical order while standing and will not be permitted to lie supine for 2 hours after dosing, except as necessitated by the occurrence of an AE(s), the underlying patients' conditions (for Part C patients with mitochondrial disease), and/or study procedures.

5.3. Randomisation

The randomisation code will be produced by the statistics department at Covance using a computer-generated pseudo-random permutation procedure. In each part of the study, 2 subjects per dose cohort will be randomly assigned to receive placebo. Subjects in

Group A1 will receive the same treatment in Treatment Periods 1 and 2. For Part C, sentinel dosing will occur whereby 2 patients (1 active and 1 placebo) will be dosed on Day 1 and, providing no safety concerns arise following the Day 4 visit, the remaining 6 patients can be dosed.

Prior to the start of the study, a copy of the master randomisation code will be supplied in sealed envelopes to the sites’ pharmacy staff and the biopharmaceutical analyst at the bioanalytical laboratory.

5.4. Blinding

The following controls will be employed to maintain the double-blind status of the study:

- The encapsulated placebo tablets will be identical in appearance to the encapsulated KL1333 tablets.
- The Investigator and other members of staff involved with the study will remain blinded to the treatment randomisation code during the assembly procedure.
- Interim bioanalytical data will be provided to Covance and the Sponsor in a blinded manner.
- Where possible, PD data will be provided to Covance and the Sponsor in a blinded manner.

To maintain the blind, the Investigator will be provided with a sealed randomisation code for each subject, containing details of their treatment. These individual sealed envelopes will be kept in a limited access area that is accessible 24 hours a day. In order to manage subject safety or to support dose escalation decisions (in the event of possibly treatment-related SAEs or severe AEs), the decision to unblind resides solely with the Investigator. Whenever possible, and providing it does not interfere with or delay any decision in the best interest of the subject, the Investigator will discuss the intended code-break with the Sponsor. If it becomes necessary to break the code during the study, the date, time, and reason will be recorded in the subject’s source data and on the individual envelope and will be witnessed by a second person.

5.5. Treatment Compliance

The following measures will be employed to ensure treatment compliance:

- For Parts A, B, and D, all doses will be administered under the supervision of suitably qualified study site staff.
- For Part C, when patients are confined in the clinical site or return to the clinical site for outpatient visits, doses will be administered under the supervision of suitably qualified study site staff. While nonresident from the clinical site, patients will self-administer the study drug and will record drug administration and any concomitant medication use in a diary. Diaries will be reviewed and checked for compliance at each visit and as part of the check-in procedures for clinical site stays.
- Immediately after dose administration in the clinical site, visual inspection of the mouth and hands will be performed for each subject.

- At each dosing occasion in the clinical site, a predose and postdose inventory of IMP will be performed on the dose containers.

For Part C, patients will be instructed that if they forget a dose while nonresident from the clinical site and remember on the day that they missed the dose, they should take their dose immediately. If they remember the following day, patients should take that day’s dose as planned and should not take 2 doses in the same day. Patients should document these deviations in their diary.

5.6. Drug Accountability

The Investigator (or designee) will maintain an accurate record of the receipt of the study supplies received. In addition, an accurate drug disposition record will be kept, specifying the amount dispensed to each subject and the date of dispensing. This drug accountability record will be available for inspection at any time. At the completion of the study, the original drug accountability record will be available for review by the Sponsor upon request.

For each batch of unit doses, the empty used unit dose containers will be discarded upon satisfactory completion of the compliance and accountability procedures. Any unused assembled unit doses will be retained until completion of the study.

At the completion of the study, all unused supplies will be returned to the Sponsor or disposed of by the study site, per the Sponsor’s written instructions.

6. CONCOMITANT THERAPIES AND OTHER RESTRICTIONS

6.1. Concomitant Therapies

Subjects in Parts A, B, and D will refrain from use of any prescription or nonprescription medications/products during the study until the Follow-up visit, unless the Investigator (or designee) and/or Sponsor have given their prior consent. Females will refrain from use of hormone replacement therapy and oral, implantable, transdermal, injectable, or intrauterine hormonal contraceptives during the study until the Follow-up visit.

For patients in Part C, hormone replacement therapy and oral, implantable, transdermal, injectable, or intrauterine hormonal contraceptives are allowed. Chronic stable medications necessary for treatment of mitochondrial disease and associated disorders will be permitted if prescribed by the subject’s personal physician and approved by the Medical Monitor and Investigator, in consultation with the Sponsor as needed. While nonresident from the clinical site, patients will record any concomitant medications in a diary that will be provided to them. Patients will refrain from use of idebenone or medications (prescription or nonprescription) that have effects on metabolism or unknown binding sites (eg, vitamin E, co-enzyme 10, arginine) during the study until the Follow-up visit.

Paracetamol/acetaminophen (2 g/day for up to 3 consecutive days) is an acceptable concomitant medication. The administration of any other concomitant medications during the study is prohibited without prior approval of the Investigator (or designee), unless its use is deemed necessary for treatment of an AE. Any medication taken by a subject during the course of the study and the reason for its use will be documented in the source data.

6.2. Diet

Subjects in Parts A, B, and D will be fasted overnight (at least 8 hours) before collection of blood samples for safety laboratory tests. Samples for safety laboratory tests in Part C should be taken at approximately the same time of day within each patient. It is recommended that patients in Part C be fasted up to 2 hours before collection of blood samples for safety laboratory tests when possible, dependent on the Investigator's judgment.

While confined at the study site, subjects will receive a standardised diet at scheduled times that do not conflict with other study-related activities. In Parts A, B, and D on days with intensive PK assessments (Day 1 for Part A and Days 1 and 10 for Parts B and D), meals will be identical for each cohort with the exception of the high-fat breakfast for Group A1.

On Day 1 of each treatment period in Part A, subjects will be fasted for at least 8 hours prior to study drug administration until 4 hours postdose, after which a meal will be provided (lunch times on Day 1 will be staggered between subjects to ensure this).

Subjects in Group A1 in Treatment Period 2 will consume a high-fat breakfast (contents are detailed in Table 5) before dosing. Subjects should start the meal 30 minutes prior to administration of the IMP. Study subjects should eat this meal in 25 minutes or less. The drug product should be administered 30 minutes after start of the meal.

Table 5: High-fat Breakfast Content

High-fat Breakfast
120 g fried eggs (2 eggs) in vegetable oil<br>50 g bacon (2 rashers)<br>72 g toasted white bread (2 slices)<br>13 g butter (2 pats)<br>108 g hash brown (3 each)<br>240 g whole milk
Total calories: 973 kcal

This high-fat meal contains the equivalent of approximately 150 protein calories, 250 carbohydrate calories, and 500 to 600 fat calories.

In Parts B and C, the time interval between meals and dosing will be determined by the PK data from Parts A and B as available and will be documented in the eCRF. Unless deemed very unfavourable for the conduct of the study, patients in Part C will not be fasted prior to dosing, except as needed for collection of blood samples. If necessary for Part C, patients will record the timing of the last meal prior to dosing and the first meal after dosing in a diary when the dose is self-administered while nonresident from the clinical site.

In Part D, subjects will be fasted for at least 8 hours prior to the first study drug administration until 4 hours after the first study drug administration on Day 1 and at least 8 hours prior to the single study drug administration until 4 hours postdose on Day 10. Subjects will also be fasted overnight (at least 8 hours) on Day 7 for collection of blood samples for safety laboratory tests prior to the first dose. All other doses of study drug can be given without regard to food.

Meals will be provided as appropriate at other times when subjects are resident at the clinical site. For Parts A and B, with the exception of water given with the dose, subjects will not be allowed fluids from 1 hour prior to dosing until 2 hours after dosing. For the first dose on Days 1 and 10 for Part D, with the exception of water given with the dose, subjects will not be allowed fluids from 1 hour prior to dosing until 2 hours after dosing. Other than these fluid restrictions, water will be freely available at all times. Water will not be restricted for patients in Part C.

For Parts A, B, and D, foods and beverages containing poppy seeds, grapefruit, or Seville oranges will not be allowed from 7 days prior to Check-in until the Follow-up visit. For Part C, patients will be encouraged to avoid or restrict their intake of grapefruit and Seville oranges from 7 days prior to Check-in until the Follow-up visit.

Consumption of alcohol will not be permitted from 36 hours prior to Check-in until the Follow-up visit.

For Parts A, B, and D, caffeine-containing foods and beverages will not be allowed from 36 hours before Check-in until the final discharge from the clinical site. Decaffeinated tea and coffee may be available with morning and evening meals, except on days of intensive blood sampling.

6.3. Smoking

For Parts A, B, and D, subjects will not be permitted to use tobacco- or nicotine-containing products within 3 months prior to Screening until the Follow-up visit.

6.4. Exercise

For Parts A, B, and D, subjects are required to refrain from strenuous exercise from 7 days before Check-in until the Follow-up visit and will otherwise maintain their normal level of physical activity during this time (ie, will not begin a new exercise program nor participate in any unusually strenuous physical exertion).

For Part C, subjects are required to maintain their normal level of physical activity as much as possible from 7 days before Check-in until the Follow-up visit.

6.5. Blood Donation

Subjects are required to refrain from donation of blood from 3 months prior to Screening, plasma from 2 weeks prior to Screening, and platelets from 6 weeks prior to Screening until 3 months after the Follow-up visit.

7. STUDY ASSESSMENTS AND PROCEDURES

Every effort will be made to schedule and perform the procedures as closely as possible to the nominal time, giving considerations to appropriate posture conditions, practical restrictions, and the other procedures to be performed at the same timepoint.

The highest priority procedures will be performed closest to the nominal time. The order of priority for scheduling procedures around a timepoint is (in descending order of priority):

- dosing
- blood samples
- any other procedures (ECGs will be scheduled before vital signs measurements).

Where activities at a given timepoint coincide, consideration must be given to ensure that the following order of activities is maintained: ECGs, vital signs, blood draws. If continuous ECG extraction windows coincide with safety ECGs, vital signs assessments, and blood draws, procedures will be carried out in said order.

7.1. Pharmacokinetic Assessments

7.1.1. Sample Collection and Processing

Blood samples (approximately 1 × 3 mL) will be collected by venepuncture or cannulation at the times indicated in the Schedule of Assessments in Appendix 5. Furthermore, up to 3 additional blood samples may be taken from each subject per treatment period. Any changes to the scheduled times of PK assessments will be agreed with the Sponsor and documented in the Trial Master File (TMF). Samples taken from subjects who received placebo will not be analysed.

Procedures for collection, processing, and shipping of PK blood samples will be detailed in a separate document.

7.1.2. Analytical Methodology

Plasma concentrations of KL1333 will be determined using validated analytical procedures. Specifics of the analytical methods will be provided in separate documents.

7.2. Pharmacodynamic Assessments

7.2.1. Sample Collection and Processing

For Part B, blood samples for NAD^+/NADH (approximately 1 × 3 mL), pyruvate (approximately 1 × 3 mL), lactate (approximately 1 × 4 mL), and other biomarker assessments and metabolomics (approximately 1 × 8.5 mL) will be collected by venepuncture or cannulation at the times indicated in the Schedule of Assessments in Appendix 5 and may be subject to change based on the ongoing review of the data. For Part C, blood samples for NAD^+/NADH (approximately 1 × 3 mL), pyruvate and lactate (approximately 1 × 4 mL), other biomarker assessments and metabolomics (approximately 1 × 8.5 mL), and glycated albumin and albumin (approximately 1 × 2.5 mL) will be collected by venepuncture or cannulation at the times indicated in the Schedule of Assessments in Appendix 5 and may be subject to change based on the ongoing review of the data. Furthermore, up to 3 additional blood samples may be taken from each subject. Any changes to the scheduled times of PD assessments will be agreed with the Sponsor and documented in the TMF. Subjects in Part B will be fasted at least 8 hours before collection of blood samples for PD. Patients in Part C may also be fasted prior to collection of blood samples for PD, dependent on the Investigator’s judgment and the results from Part B.

For Part D, blood samples for NAD^+/NADH (approximately 1 × 3 mL), pyruvate and lactate (approximately 1 × 4 mL), other serum biomarker assessments and metabolomics (approximately 1 × 8.5 mL), and other whole blood biomarker assessments and metabolomics (approximately 1 × 3 mL) will be collected by venepuncture or cannulation at the times indicated in the Schedule of Assessments in Appendix 5 and may be subject to change based on the ongoing review of the data. Furthermore, up to 3 additional blood samples may be taken from each subject. Any changes to the scheduled times of PD assessments will be agreed with the Sponsor and documented in the TMF. Fasting for subjects in Part D will occur as outlined in Section 6.2.

Procedures for collection, processing, and shipping of PD blood samples will be detailed in a separate document.

Metabolomics samples will be stored for potential later analysis and will not be reported in the scope of this study.

7.2.2. Analytical Methodology

Validated analytical procedures will be used to determine NAD^+/NADH concentrations and ratio, FGF21, GDF15, lactate/pyruvate concentrations and ratio, and glycated albumin/albumin concentrations and ratio. Specifics of the analytical methods will be provided in separate documents. Any remaining sample will be stored for potential later analysis of additional biomarkers.

7.2.3. Clinical Assessments

The following clinical assessments will be performed at the times indicated in the Schedule of Assessments in Appendix 5.

7.2.3.1. Newcastle Mitochondrial Disease Adult Scale

The NMDAS is a validated clinical rating scale designed to capture the natural history of mitochondrial disease.[3] The NMDAS includes 3 domains: current function, system specific involvement, and current clinical, assessed on 6-point Likert-type scale from 0 to 5, as well as a fourth section including a score for the 12-Item Short Form Survey-Version 2.

An experienced clinician can use the NMDAS after a short training session. Clinicians who are deemed to be sufficiently experienced in conducting the scales by the Principal Investigator will not be required to attend a training session and may perform the assessments upon delegation of this task by the Principal Investigator. Cognitive raters who are deemed sufficiently qualified as determined by the Principal Investigator may perform cognitive assessments as part of the NMDAS. It takes approximately 25 to 30 minutes to administer and score the NMDAS.

7.2.3.2. Clinical Global Impression

The CGI is a 2-item observer-rated scale that measures illness severity and global improvement or change.[4] The CGI is rated on a 7-point Likert-type scale, with the severity of illness scale using a range of responses from 1 (normal) to 7 (amongst the most severely ill patients).

An experienced clinician can use the CGI after a short training session. Clinicians who are deemed to be sufficiently experienced in conducting the scales by the Principal Investigator will not be required to attend a training session and may perform the assessments upon delegation of this task by the Principal Investigator. It takes approximately 5 minutes to administer and score the CGI.

7.2.3.3. Patient Global Impression-Improvement

The PGI-I is a patient-rated scale using a 5-point Likert-type scale to assess the severity of illness using a range of responses from 1 (no symptoms) to 5 (very severe symptoms) and a 7-point Likert-type scale using a range of responses from 1 (very much improved) to 7 (very much worse) to assess improvement.[5]

7.2.3.4. Daily Fatigue Impact Severity

The D-FIS is a patient-rated scale developed to assess the symptom of fatigue as part of an underlying chronic disease or condition.[6] The D-FIS includes 8 items assessed on 5-point Likert-type scale from 0 (no problem) to 4 (extreme problem).

7.2.3.5. Quality of Life in Neurological Disorders Fatigue Short Form

The Neuro-QoL Fatigue Short Form is one of several scales that make up the Quality of Life in Neurological Disorders measurement system.[7] It is a reliable and validated brief 8-item survey of fatigue, completed by the subject, with a recall period of the past 7 days. The 8 items are scored from 1 (never) to 5 (always) and, consequently, Neuro-QoL Fatigue Short Form total scores range from 8 to 40, with higher scores indicating greater fatigue and greater impact of mitochondrial disease on activities.

7.2.3.6. Thirty Second Sit-to-Stand Test

The 30 Second Sit-to-Stand Test is a performance test also known as the 30 Second Chair Stand Test.[8] The test requires a straight back chair with a 43 to 46 cm seat height and no arm rests backed against wall to prevent slipping and a stopwatch/timer. The same chair should be used for retesting.

7.3. Safety and Tolerability Assessments

7.3.1. Adverse Events

Adverse event definitions, assignment of severity and causality, and procedures for reporting SAEs are detailed in Appendix 1. See Section 7 of the IB[1] for the reference safety information.

The condition of each subject will be monitored from the time of signing the ICF to final discharge from the study. Subjects will be observed for any signs or symptoms and asked about their condition by open questioning, such as “How have you been feeling since you were last asked?”, at least once each day while resident at the study site and at each study visit. Subjects will also be encouraged to spontaneously report AEs occurring at any other time during the study. For Part C, patients will record any clinical symptoms while they are nonresident from the clinical site in a diary that will be provided to them.

Any AEs and remedial action required will be recorded in the subject's source data. The nature, time of onset, duration, and severity will be documented, together with an Investigator's (or designee's) opinion of the relationship to study drug.

Adverse events recorded during the course of the study will be followed up, where possible, until resolution or until the unresolved AEs are judged by the Investigator (or designee) to have stabilised. This will be completed at the Investigator's (or designee's) discretion.

7.3.2. Clinical Laboratory Evaluations

Blood and urine samples will be collected for clinical laboratory evaluations (including clinical chemistry, haematology, urinalysis, and serology) at the times indicated in the Schedule of Assessments in Appendix 5. Clinical laboratory evaluations are listed in Appendix 2. Additional clinical laboratory evaluations will be performed at other times if judged to be clinically appropriate or if the ongoing review of the data suggests a more detailed assessment of clinical laboratory safety evaluations is required.

All subjects will be asked to provide urine samples for drugs of abuse screen and will undergo an alcohol breath test at the times indicated in the Schedule of Assessments in Appendix 5. Subjects in Parts A, B, and D will also provide urine samples for cotinine testing at the times indicated in the Schedule of Assessments in Appendix 5. Subjects in Part C will have glycated haemoglobin measured at Screening. For all female subjects, a pregnancy test will be performed at the times indicated in the Schedule of Assessments in Appendix 5. For all postmenopausal female subjects, follicle-stimulating hormone will be assessed at Screening.

An Investigator (or designee) will perform a clinical assessment of all clinical laboratory data.

7.3.3. General and Gastrointestinal Inflammation Assessments

For Group B5 and Part D, and optionally for Part C, levels of general and gastrointestinal inflammation will be assessed with CRP, ESR, and faecal calprotectin. Blood samples for CRP and ESR and faecal samples for faecal calprotectin will be collected at the times indicated in the Schedule of Assessments in Appendix 5. Up to 3 additional symptom-directed evaluations may be performed based on the Investigator's judgment. An Investigator (or designee) will perform a clinical assessment of the CRP, ESR, and faecal calprotectin data.

7.3.4. Vital Signs

Supine and standing blood pressure, supine and standing pulse rate, and oral body temperature will be assessed at the times indicated in the Schedule of Assessments in Appendix 5. Vital signs may also be performed at other times if judged to be clinically appropriate or if the ongoing review of the data suggests a more detailed assessment of vital signs is required.

Blood pressure and pulse rate will be measured in triplicate at approximately 2-minute intervals at Day 1 predose for Parts A through C and Day 1 prior to the first dose in Part D. The median value will be used as the baseline value in the data analysis. All subsequent

measurements will be performed singly and repeated once if outside the relevant clinical reference ranges. Oral body temperature will be measured singly.

Subjects must be supine for at least 5 minutes before blood pressure and pulse rate measurements.

For orthostatic vital signs measurements, the supine blood pressure and pulse rate will be measured after the subject has been supine for at least 5 minutes. The subject will then stand for at least 2 minutes and the standing blood pressure and pulse rate will be measured.

7.3.5. Electrocardiogram

7.3.5.1. Safety 12-Lead Electrocardiogram

Resting 12-lead ECGs will be recorded after the subject has been supine and at rest for at least 5 minutes at the times indicated in the Schedule of Assessments in Appendix 5. In Parts A and B, these safety ECGs can be obtained from the digital recorder used for continuous ECG monitoring or from a separate ECG machine. Twelve-lead ECGs will be repeated if either of the following criteria apply:

- QTcF value >500 msec
- QTcF change from the baseline (Day -1) is >60 msec.

Additional 12-lead ECGs may be performed at other times if judged to be clinically appropriate or if the ongoing review of the data suggests a more detailed assessment of ECGs is required. The Investigator (or designee) will perform a clinical assessment of each 12-lead ECG.

For Parts A, B, and D, Day -1 baseline 12-lead ECGs will be measured in triplicate at approximately 2-minute intervals. The mean value will be used as the baseline value in the data analysis. All subsequent measurements will be performed singly and repeated once if outside the relevant clinical reference ranges. For Part C, all ECG measurements will be performed in triplicate at approximately 3- to 5-minute intervals and repeats will be performed in triplicate if outside the relevant clinical reference ranges.

7.3.5.2. Continuous 12-lead Electrocardiogram Monitoring

Continuous 12-lead ECG monitoring using a Mortara Surveyor system will be performed in Parts A and B only at the times indicated in the Schedule of Assessments in Appendix 5. If necessary, the predose timepoints can occur up to 20 minutes prior to the indicated time.

Subjects will be supine for at least 5 minutes before the extraction timepoint and for 5 minutes from the start of each extraction timepoint (each extraction will last for 5 minutes). Environmental distractions (eg, television, radio, conversation) should be avoided during the pre-ECG resting period and during ECG recording.

When coinciding, vital signs assessments and PK sampling should always be performed after the ECG extraction time window. If a separate ECG machine is being used for safety assessments described in Section 7.3.5.1, that machine should be in place prior to the

extraction window to permit safety ECGs to be recorded irrespective of the extraction window. If the machine is not in place prior to the extraction window, safety ECGs must be recorded after the extraction window. If an integral system is used, safety ECGs may be recorded irrespective of the extraction window.

All continuous ECG data collected on study will be archived without extraction or analysis. If the decision is made to extract and analyse the continuous ECG data, the following will be the procedure at the central ECG laboratory (ERT, Rochester, New York, USA), and the analysis will not be reported in the scope of this study:

The continuous 12-lead digital ECG data will be stored prior to uploading to ERT. The ECGs to be used in the analyses will be selected by pre-determined timepoints as defined in the Schedule of Assessments in Appendix 5 and will be read centrally by ERT.

The following principals will be followed in ERT's core laboratory:

- ECG analysts are blinded to the subject, visit, and treatment allocation.
- Baseline and on-treatment ECGs for a particular subject will be over-read on the same lead and will be analysed by the same reader.
- The primary analysis lead is lead II. If lead II is not analysable, then the primary lead of analysis will be changed to another lead for the entire subject data set.

The following is a brief description of ECG analysis methods utilised by ERT's core laboratory.

7.3.5.3. TQT Plus ECG Extraction Technique

Ten 14-second digital 12-lead ECG tracings will be extracted from the continuous Holter recordings using the 'TQT Plus method,' a computer-assisted and statistical process utilised by ERT. The method enables extraction of ECGs with the lowest HR variability and noise within the protocol-specified extraction time window (eg, the HR and QT changes from beat-to-beat in the range of <10%). At each protocol-specified timepoint, 10 ECG replicates will be extracted from a 5-minute "ECG window" (typically, the last 5 minutes of the 15-minute period when the subject is maintained in a supine or semi-recumbent quiet position).

7.3.5.4. Expert-precision QT Analysis

Expert-precision QT analysis will be performed on all analysable (nonartifact) beats in the 10 ECG replicates. Statistical quality control procedures will be used to review and assess all beats and identify "high" and "low" confidence beats using several criteria, including:

- QT or QTc values exceeding or below certain thresholds (biologically unlikely)
- RR values exceeding or below certain thresholds (biologically unlikely)
- rapid changes in QT, QTc, or RR from beat to beat.

Measurements of all primary ECG parameters (QT, QTc, RR) in all recorded beats of all replicates that are deemed "high confidence" will be performed using COMPAS software.

All low confidence beats will be reviewed manually and adjudicated using pass-fail criteria. The final quality control assessment will be performed by a cardiologist. The beats found acceptable by manual review will be included in the analysis. The median QT, QTc, and RR value from each extracted replicate will be calculated, and then the mean of all available medians from a nominal timepoint will be used as the subject's reportable value at that timepoint.

Categorical T-wave morphology analysis (Table 6) and the measurement of PR and QRS intervals will be performed manually in 3 of the 10 ECG replicates at each timepoint. Each fiducial point (onset of P wave, onset of Q wave, offset of S wave, and offset of T wave) will be electronically marked.

Table 6: T-wave Morphology Categories (Assessed Manually)

Category	Description
Normal T wave	Any T wave not meeting any criterion below
Flat T waves	T-wave amplitude <1 mm (either positive or negative) including flat isoelectric line
Notched T wave (+)	Presence of notch(es) of at least 0.05 mV amplitude on ascending or descending arm of the positive T wave
Biphasic	T wave that contains a second component with an opposite phase that is at least 0.1 mV deep (both positive and negative/positive and polyphasic T waves included)
Normal T wave (-)	T-wave amplitude that is negative, without biphasic T wave or notches
Notched T wave (-)	Presence of notch(es) of at least 0.05 mV amplitude on descending or ascending arm of the negative T wave

In addition to the T-wave categorical analysis, the presence of abnormal U waves will be noted.

7.3.6. Physical Examination

A complete physical examination or symptom-directed physical examination will be performed at the timepoints specified in the Schedule of Assessments in Appendix 5. The complete physical examination will include the following systems: general appearance; skin; lymph nodes; head, ears, eyes, nose, and throat; neck; thorax/lungs; cardiovascular; abdomen; musculoskeletal; and neurological.

7.3.7. Body Weight

Body weight (in underclothes) will be recorded at the times indicated in the Schedule of Assessments in Appendix 5.

7.3.8. Genotyping

A 3-mL blood sample for genotyping will be collected at Check-in. Analysis of NQO1 may be performed. Any remaining genotyping sample will be stored in case additional genotyping of uridine 5'-diphospho-glucuronosyltransferase or CYP enzymes would aid in the interpretation of the study results.

7.3.9. Exit Interview

An exit interview will be conducted with all patients in Part C on Day 11. Patients will be asked their perspective on their experiences during study participation.

8. SAMPLE SIZE AND STATISTICAL DATA ANALYSIS

A Statistical Analysis Plan (SAP) describing the handling of data issues and the planned statistical analyses in more detail will be prepared by Covance's Biostatistics Department before database release.

8.1. Determination of Sample Size

No formal statistical assessment, in terms of sample size, has been conducted. However, the number of subjects in each part of the present study is common in early clinical pharmacology studies and is considered sufficient to achieve the objectives of the study.

8.2. Analysis Populations

8.2.1. Pharmacokinetic Population

The PK population will include all subjects who received at least 1 dose of KL1333 and have evaluable PK data. A subject may be excluded from the PK summary statistics and statistical analysis if the subject has an AE of vomiting that occurs at or before 2 times median T_{max}.

8.2.2. Pharmacodynamic Population

The PD population will include all subjects who received at least 1 dose of study treatment (KL1333 or placebo) and for whom PD markers can be evaluated.

8.2.3. Safety Population

The safety population will include all subjects who received at least 1 dose of study treatment (KL1333 or placebo) and have at least 1 postdose safety assessment.

8.2.4. QT/QTc Population

The QT/QTc population will include all subjects in the safety population who had measurements at baseline as well as on treatment, with at least 1 postdose timepoint with a valid ΔQTcF value. This analysis population will be used for the by-timepoint and categorical analyses for the cardiodynamic ECG parameters.

8.2.5. PK/QTc Population

The PK/QTc population will include all subjects who are in both the QT/QTc and PK populations with at least 1 pair of postdose PK and QTcF data from the same timepoint. This analysis population will be used for the concentration-QTc analysis.

8.3. Pharmacokinetic Analyses

Noncompartmental PK analysis will be performed on individual plasma concentration data, using commercial software such as Phoenix WinNonlin®. Plasma concentrations of KL1333 and PK parameters will be listed and summarised using descriptive statistics. Individual and mean KL1333 concentration-time profiles will also be presented graphically.

In Part A, where data are available, the effect of food on KL1333 will be investigated using an analysis of variance (ANOVA) model as appropriate. In Part B, where data are available, KL1333 dose proportionality will be examined across the dose cohorts. The PK parameters will be analysed for dose proportionality using a power model approach or ANOVA model as appropriate.

8.4. Pharmacodynamic Analyses

Pharmacodynamic parameters will be listed and summarised using descriptive statistics. Formal statistical analysis of PD data in Parts C and D is planned and will be described in more detail in the SAP.

8.5. Safety Analysis

Safety parameters will be listed and summarised using descriptive statistics. No formal statistical analysis of safety data is planned. Each AE will be coded using the Medical Dictionary for Regulatory Activities. The number of subjects with abnormalities in clinical laboratory measurements and the number of subjects with vital signs measurements or ECG parameters meeting criteria of potential clinical concern from baseline through the Follow-up visit will be summarised.

The vital signs criteria of potential clinical concern are as follows:

- supine pulse rate <40 bpm or >120 bpm
- standing pulse rate <40 bpm or >140 bpm
- systolic blood pressure ≥30 mmHg change from baseline in same posture or <90 mmHg
- diastolic blood pressure ≥20 mmHg change from baseline in same posture or <50 mmHg.

The ECG criteria of potential clinical concern are as follows:

- PR interval ≥300 msec, ≥25% increase when baseline is >200 msec, or ≥50% increase when baseline is ≤200 msec
- QRS interval ≥140 msec or ≥50% increase from baseline
- QTcF ≥450 msec to <480 msec, ≥480 msec to <500 msec, or ≥500 msec.

8.5.1. Cardiodynamic Electrocardiogram Evaluation

This section addresses the cardiodynamic ECG assessment from continuous 12-lead ECG recordings (Holters) for Parts A and B only. The primary analysis will be based on concentration-QTc modelling of the relationship between KL1333 and ΔQTcF with the intent to exclude an effect >10 msec at clinically relevant KL1333 plasma concentrations. In addition, the effect of KL1333 on the ΔΔQTcF will be evaluated at each postdose timepoint ('by-timepoint' analysis) using the Intersection Union Test. An analysis of categorical outliers will be performed for changes in HR; PR, QRS, and QTcF intervals; T-wave morphology; and U-wave presence.

8.5.1.1. General Methodology

All statistical analyses will be performed using the statistical software SAS for Windows Version 9.4 or newer (SAS Institute, Inc., Cary, NC, USA). In all calculations, zero will be substituted for concentrations below the quantification limit of the assay. Data collected from all randomised subjects will be presented in data listings. Both absolute values and change-from-baseline values for each subject will be given where applicable. All continuous data will be listed with the same precision as will be presented in the database. Data listings will be sorted by treatment, subject number, and timepoint. Missing values will be represented by an empty cell and no imputation will be made.

Continuous data will be summarised using descriptive statistics including number of subjects, mean, median, standard deviation (SD), standard error (SE), 90% confidence interval (CI), minimum, and maximum by treatment and timepoint. Mean and median values will be rounded to the nearest tenth, or to the first nonzero decimal; SD, SE, and CI will be rounded to the nearest hundredth, or to 1 digit more than the nearest nonzero digit. For the concentration-QTc analysis, 3 significant figures will be kept for the effect estimates. *P*-values will be reported with 4 significant digits, except *p*-values less than 0.0001, which will be reported as <0.0001. Categorical data will be summarised 2 ways, by subject and by timepoint. Subject data will be summarised using the count of distinct subjects that fall into the category and the percentage of the total number of subjects. Timepoint data will be summarised using the count of the assessments that fall into the category and the percentage of the total number of assessments. Percentages will be rounded up or down to the next integer percentage. Population counts (either number of subjects or number of timepoints at the assessment) for each treatment group will be used as the denominator in the calculation of percentages unless otherwise specified.

8.5.1.2. Baseline

For all continuous ECG parameters, baseline is defined as the mean of the measured ECG intervals from the 3 ECG timepoints recorded predose (-45, -30, and -15 minutes) on Day 1. For T-wave morphology and U-wave presence, baselines include all categories that occur across all replicates at the 3 predose timepoints on Day 1.

8.5.1.3. Concentration-QTc Analysis (Primary Analysis)

The relationship between KL1333 plasma concentration and ΔQTcF will be quantified using a linear mixed-effects modelling approach with ΔQTcF as a dependent variable, drug plasma

concentration as a continuous covariate (ie, 0 for placebo), centred baseline QTcF (ie, baseline QTcF for individual subject at each postbaseline timepoint subtracting the population mean baseline QTcF for all subjects) as an additional covariate, study treatment (active = 1 or placebo = 0) and time (ie, all postbaseline timepoints on Days 1, 2, and 10) as categorical factors, and a random intercept and slope per subject.[9] The degree of freedom estimates will be determined by the Kenward-Roger method. From the model, the slope (ie, the regression parameter for the concentration) and the treatment effect-specific intercept (defined as the difference between active and placebo) will be estimated together with 2-sided 90% CIs. The estimates for the time effects will be reported with degrees of freedom and SE.

The geometric mean of the individual C_{max} values for subjects at each dose of active drug will be determined. The predicted effect and its 2-sided 90% CI for ΔΔQTcF (ie, slope estimate + treatment effect-specific intercept) at this geometric mean C_{max} will be obtained. If the upper bound of the 2-sided 90% CI of the model-predicted QTcF effect is below 10 msec at clinically relevant plasma levels of KL1333, it will be concluded that KL1333 does not cause clinically-concerning QTc prolongation.

The plot of the observed median-quantile KL1333 concentrations and associated mean ΔΔQTcF (90% CI) adjusted for diurnal effects together with the regression line presenting the predicted ΔΔQTcF will be used to evaluate the adequacy of the model fit to the assumption of linearity and the impact on quantifying the concentration-response relationship.[10] The observed ΔQTcF values from the active groups will be adjusted by the estimated time effect from the concentration-QTc model (ie, the estimated diurnal effect under the placebo treatment). The individually estimated $\Delta\Delta QTcF_{i,j}$ equals the individual $\Delta QTcF_{i,j}$ for subject i administered with KL1333 at timepoint j minus the estimation of time at timepoint j (ie, time effect). Additional exploratory analyses (via graphical displays and/or model fitting) will include accounting for a delayed effect (hysteresis) and the justification for the choice of the PD model (linear versus nonlinear) as follows.

8.5.1.3.1. Investigation of Hysteresis

Hysteresis will be assessed by graphical methods based on the least squares (LS) mean difference of ΔQTcF between KL1333 and placebo (ΔΔQTcF) for each postbaseline timepoint and the mean concentrations of KL1333 at the same timepoints. In addition, hysteresis plots will be given for mean ΔΔQTcF and the mean concentrations. If a QT effect (ΔΔQTcF) >10 msec cannot be excluded in the 2 highest dose groups from the by-timepoint analysis and if a delay between peak plasma levels and peak QT effect of more than 1 hour is present, other concentration-QTc models such as a model with an effect compartment may be explored. With the provision stated above, hysteresis will be assumed if the curve shows a counter clockwise loop. A significant treatment effect-specific intercept is not biologically plausible and therefore may also be indicative of hysteresis, if it cannot be explained by a nonlinear relationship.

8.5.1.3.2. Appropriateness of a Linear Model

To assess the appropriateness of a linear model, normal Q-Q plots for the standardised residuals and the random effects, and plots of standardised residuals versus concentration, fitted values, centred baseline QTcF, nominal time, and active treatment will be produced,

respectively. The scatter plots of standardised residuals versus concentration and centred baseline QTcF by LOESS fitting (ie, locally weighted scatterplot smoothing[11]) will also be produced with optimal smoothing parameters selected by the Akaike information criterion with a correction.[12] In addition, a model with the original term and a quadratic term in concentration will be fitted and the quadratic term will be tested on the 2-sided 5% level. If there is an indication that a linear model is inappropriate, additional models will be fitted, in particular:

- An E_{max} model: ΔQTcF ~ Treatment + E_{max} × Concentration / (EC_{50} + Concentration) + Time + Centred baseline QTcF, where the parameters E_{max}, and EC_{50} are the maximum effect attributable to the drug; and the dose that produces half of E_{max}, respectively.
- A log-transformation model where the KL1333 plasma concentration C is replaced by $\log(C/C_0)$, C_0 is the limit of quantification of the assay used to determine C, and all values below C_0 are replaced by C_0 (ie, $\log[C_0/C_0] = 0$).

The concentration-QTc analysis will then be repeated for the model found to best accommodate the nonlinearity detected.

8.5.1.4. By-timepoint Analysis

The analysis for QTcF will be based on a linear mixed-effects model with ΔQTcF as the dependent variable, time (ie, all postbaseline timepoints on Days 1, 2, and 10: categorical), treatment (KL1333 and pooled placebo), and time by treatment interaction as fixed effects, and baseline QTcF as a covariate. An unstructured covariance matrix will be specified for the repeated measures at postbaseline timepoints within subject. If the model with an unstructured covariance matrix fails to converge, another covariance matrix such as compound symmetry and autoregressive will be considered. From this analysis, the LS mean and a 2-sided 90% CI will be calculated for the contrasts "KL1333 – placebo" for each dose and each postbaseline timepoint on Days 1, 2, and 10, separately.

For HR, PR, and QRS the analysis will be based on the change from baseline postdosing (ΔHR, ΔPR, and ΔQRS). The same (by-timepoint analysis) model will be used as described for QTcF. The LS mean, SE, and 90% CI from the statistical modelling for both change-from-baseline and the time-matched, placebo-adjusted change-from-baseline values will be listed in the tables and graphically displayed.

8.5.1.5. Categorical Analysis

The analyses results for categorical outliers, T-wave morphology, and U-wave presence will be summarised in frequency tables with counts and percentages for both number of subjects and number of timepoints. For categorical outliers, the number (percentage) of subjects as well as timepoints who had increases in absolute QTcF values >450 and ≤480 ms, >480 and ≤500 ms, or >500 ms, and changes from predose baseline of >30 and ≤60 ms, or >60 ms; increase in PR from predose baseline >25% to a PR >200 ms; increase in QRS from predose baseline >25% to a QRS >120 ms; decrease in HR from predose baseline >25% to an HR <50 bpm; and increase in HR from predose baseline >25% to an HR >100 bpm will be determined. For T-wave morphology and U-wave presence, the analysis will be focused on change from baseline (ie, treatment-emergent changes [across all timepoints]).

8.6. Interim Analysis

An interim analysis will be performed following completion of each cohort in Parts A and B to review safety, tolerability, and PK data and determine the dose level, dose frequency, and dietary state of the next cohort. Dose escalation is described in Section 3.6, and stopping criteria are provided in Section 3.7.

9. REFERENCES

1. Abliva AB. KL1333 – Investigator's Brochure (current version).

2. Kelsey KT, Ross D, Traver RD, et al. Ethnic variation in the prevalence of a common NAD(P)H quinone oxidoreductase polymorphism and its implications for anti-cancer chemotherapy. *British J Cancer*. 1997;76(7):852-854.

3. Schaefer AM, Phoenix C, Elson JL, McFarland R, Chinnery PF, Turnbull DM. Mitochondrial disease in adults: a scale to monitor progression and treatment. *Neurology*. 2006;66(12):1932-1934.

4. Guy W, editor. *ECDEU Assessment Manual for Psychopharmacology*. Rockville, MD: US Dept. of Health, Education, and Welfare, Public Health Service, Alcohol, Drug Abuse, and Mental Health Administration, National Institute of Mental Health, Psychopharmacology Research Branch, Division of Extramural Research Programs; 1976.

5. Arnold LM, Zlateva G, Sadosky A, Emir B, Whalen E. Correlations between fibromyalgia symptom and function domains and patient global impression of change: a pooled analysis of three randomized, placebo-controlled trials of pregabalin. *Pain Med*. 2011;12(2):260-267.

6. Fisk JD, Doble SE. Construction and validation of a fatigue impact scale for daily administration (D-FIS). *Qual Life Res*. 2002;11(3):263-272.

7. Cella D, Lai JS, Nowinski CJ, et al. Neuro-QOL: Brief measures of health-related quality of life for clinical research in neurology. *Neurology*. 2012;78(23):1860-1867.

8. Tveter AT, Dagfinrud H, Moseng T, Holm I. Measuring health-related physical fitness in physiotherapy practice: reliability, validity, and feasibility of clinical field tests and a patient-reported measure. *J Orthop Sports Phys Ther*. 2014;44(3):206-216.

9. Garnett C, Bonate PL, Dang Q, et al. Scientific white paper on concentration-QTc modeling. *J Pharmacokinet Pharmacodyn*. 2018;45(3):383-397.

10. Tornøe CW, Garnett CE, Wang Y, Florian J, Li M, Gobburu JV. Creation of a knowledge management system for QT analyses. *J Clin Pharmacol*. 2011;51(7):1035-1042.

11. Cleveland WS. Robust Locally Weighted Regression and Smoothing Scatterplots. *J Amer Statist Assoc*. 1979;74(368):829-836.

12. Hurvich CM, Simonoff JS, Tsai CL. Smoothing parameter selection in nonparametric regression using an improved Akaike information criterion. *J Roy Statist Soc Ser B*. 1998;60(2):271-293.

13. International Committee of Medical Journal Editors. Recommendations for the conduct, reporting, editing, and publication of scholarly work in medical journals. Available at: icmje.org/urm_main.html. August 2013.

10. APPENDICES

Appendix 1: Adverse Event Reporting

Definitions

An adverse event (AE) is any untoward medical occurrence in a patient or clinical investigation subject administered a pharmaceutical product, which does not necessarily have a causal relationship with this treatment. An AE can therefore be any unfavourable and/or unintended sign (including a clinically significant abnormal laboratory finding), symptom, or disease temporally associated with the use of a study drug, whether or not related to the study drug.

Assessment of Severity

The Investigator will be asked to provide an assessment of the severity of the AE using the following categories:

- **Mild:** Usually transient and may require only minimal treatment or therapeutic intervention. The event does not generally interfere with usual activities of daily living.
- **Moderate:** Usually alleviated with additional specific therapeutic intervention. The event interferes with usual activities of daily living, causing discomfort, but poses no significant or permanent risk of harm to the subject.
- **Severe:** Interrupts usual activities of daily living, significantly affects clinical status, or may require intensive therapeutic intervention.

Relationship to Study Treatment

The Investigator (or designee) will make a determination of the relationship of the AE to the study drug using a 4-category system according to the following guidelines:

- **Not Related:** The AE is definitely caused by the subject’s clinical state or the study procedure/conditions.
- **Unlikely Related:** The temporal association between the AE and the drug is such that the drug is not likely to have any reasonable association with the AE.
- **Possibly Related:** The AE follows a reasonable temporal sequence from the time of drug administration but could have been produced by the subject’s clinical state or the study procedures/conditions.
- **Related:** The AE follows a reasonable temporal sequence from administration of the drug, abates upon discontinuation of the drug, follows a known or hypothesized cause-effect relationship, and (if appropriate) reappears when the drug is reintroduced.

Follow-up of Adverse Events

Every reasonable effort will be made to follow-up with subjects who have AEs. Any subject who has an ongoing AE that is possibly related or related to the investigational medicinal product (IMP) or study procedures at the Follow-up visit will be followed up, where possible, until resolution or until the unresolved AE is judged by the Investigator (or designee) to have

stabilised. This will be completed at the Investigator's (or designee's) discretion. Any subject who has an ongoing AE that is not related or unlikely related to the IMP or study procedures at the Follow-up visit can be closed out as ongoing at the Investigator's discretion.

Adverse Drug Reactions

All noxious and unintended responses to an IMP (ie, where a causal relationship between an IMP and an AE is at least a reasonable possibility) related to any dose should be considered adverse drug reactions.

For marketed IMPs, a response to a drug that is noxious and unintended and that occurs at doses normally used in man for prophylaxis, diagnosis, or therapy of diseases or for modification of physiological function is to be considered an adverse drug reaction.

An unexpected adverse drug reaction is defined as an adverse reaction, the nature or severity of which is not consistent with the applicable product information (eg, Investigator's Brochure [IB] for an unapproved IMP).

Serious Adverse Events

A serious AE (SAE) is defined as any untoward medical occurrence that at any dose either:

- results in death
- is life threatening
- requires inpatient hospitalisation or prolongation of existing hospitalisation
- results in persistent or significant disability/incapacity (disability is defined as a substantial disruption of a person's ability to conduct normal life functions)
- results in a congenital anomaly/birth defect
- results in an important medical event (see below).

Important medical events that may not result in death, be life threatening, or require hospitalisation may be considered SAEs when, based upon appropriate medical judgment, they may jeopardise the subject and may require medical or surgical intervention to prevent one of the outcomes listed in this definition.

Instances of death or congenital abnormality, if brought to the attention of the Investigator at any time after cessation of the study treatment and considered by the Investigator to be possibly related to the study treatment, will be reported to the Sponsor.

<u>Definition of Life Threatening</u>

An AE is life threatening if the subject was at immediate risk of death from the event as it occurred (ie, does not include a reaction that might have caused death if it had occurred in a more serious form). For instance, drug-induced hepatitis that resolved without evidence of hepatic failure would not be considered life threatening even though drug-induced hepatitis can be fatal.

Definition of Hospitalisation

Adverse events requiring hospitalisation should be considered serious. In general, hospitalisation signifies that the subject has been detained (usually involving an overnight stay) at the hospital or emergency ward for observation and/or treatment that would not have been appropriate at the clinical site. When in doubt as to whether hospitalisation occurred or was necessary, the AE should be considered as serious.

Hospitalisation for elective surgery or routine clinical procedures, which are not the result of an AE, need not be considered AEs and should be recorded on a Clinical Assessment Form and added to the electronic Case Report Form. If anything untoward is reported during the procedure, this must be reported as an AE and either 'serious' or 'nonserious' attributed according to the usual criteria.

Serious Adverse Event Reporting

Covance Drug Safety Services (DSS) Europe, Maidenhead, UK, are responsible for coordinating the reporting of SAEs in accordance with the European Directive 2001/20/EC.

The Investigator will complete an SAE report form and forward it by facsimile or email to DSS and the Sponsor immediately (within 24 hours) upon becoming aware of an SAE.

The responsibilities of Covance DSS include the following:

- Prepare an AE reporting plan prior to the start of the study. Where this plan differs from the applicable clinical site standard operating procedure on SAE reporting, the Safety Management Plan will always take precedence.
- Receive and review SAE report forms from the clinical site and inform the Sponsor of the SAE within 2 working days of the initial notification to DSS. Drug Safety Services will delete any information from the SAE report forms that may identify the subject.
- Write case narratives and enter the case into Covance's safety database as defined in the AE reporting plan.
- Produce appropriate reports of all Suspected Unexpected Serious Adverse Reactions (SUSARs) and forward to the Ethics Committee, Medicines and Healthcare Products Regulatory Agency, Principal Investigator, and the Sponsor within the timeframes stipulated in the Clinical Trials Directive Guideline (ENTR/CT 3). A SUSAR is any AE that is assessed as serious, unexpected (its nature or intensity is not consistent with the current version of the reference safety information in the IB), and assessed as related to an IMP by either the Investigator or the Sponsor.

The responsibility for reporting SAEs will be transferred to the Sponsor 28 days after the end of the study.

Pregnancy

Pregnancy (maternal or paternal exposure to study drug) does not meet the definition of an AE. However, to fulfil regulatory requirements, any pregnancy should be reported following the SAE process to collect data on the outcome for both mother and foetus.

Appendix 2: Clinical Laboratory Evaluations

Clinical chemistry:	Haematology:	Urinalysis:
Alanine aminotransferase<br>Albumin<br>Alkaline phosphatase<br>Aspartate aminotransferase<br>Calcium<br>Chloride<br>Cholesterol<br>Creatinine kinase<br>Creatinine<br>Creatinine clearance[a]<br>Direct bilirubin<br>Gamma-glutamyl transferase<br>Glucose<br>Inorganic phosphate<br>Potassium<br>Sodium<br>Total bilirubin<br>Total protein<br>Uric acid	Haematocrit<br>Haemoglobin<br>Mean cell haemoglobin<br>Mean cell haemoglobin concentration<br>Mean cell volume<br>Platelet count<br>Red blood cell count<br>White blood cell (WBC) count<br>WBC differential:<br>Basophils<br>Eosinophils<br>Lymphocytes<br>Monocytes<br>Neutrophils	Blood<br>Glucose<br>Ketones<br>pH<br>Protein<br>Specific gravity<br>Urobilinogen<br>Microscopic examination
**Serology[b]:**	**Drug screen[c]:**	**Hormone panel - females only:**
Anti-hepatitis B surface antibody<br>Hepatitis B surface antigen<br>Hepatitis C antibody<br>Human immunodeficiency virus (HIV-1 and HIV-2) antibodies	Including but not limited to:<br>Amphetamines/methamphetamines<br>Barbiturates<br>Benzodiazepines<br>Cotinine[g]<br>Cocaine (metabolite)<br>Methadone<br>Phencyclidine<br>Opiates<br>Tetrahydrocannabinol/cannabinoids<br>Tricyclic antidepressants<br>Alcohol breath test	Follicle-stimulating hormone[d]<br>Serum pregnancy test (human chorionic gonadotropin)[e]<br>Urine pregnancy test[f]
**Other tests:**		
C-reactive protein[h]<br>Erythrocyte sedimentation rate[h]<br>Faecal calprotectin[h]<br>Glycated haemoglobin[a]		

[a] Only analysed at Screening in Part C.
[b] Only analysed at Screening.
[c] Analysed at Screening and Check-in for Parts A, B, and D, and analysed at Screening, Check-in, and Day 10 for Part C.
[d] In postmenopausal females.
[e] Performed at Screening for all females.
[f] Performed for all females at Check-in, early termination, and Follow-up visit. A positive urine pregnancy test will be confirmed with a serum pregnancy test.
[g] Only for Parts A, B, and D.
[h] For Group B5 and Part D, and if required/indicated in Part C.

Appendix 3: Contraception Guidance

Definitions

Women of Childbearing Potential: Premenopausal females who are anatomically and physiologically capable of becoming pregnant following menarche.

Women of Nonchildbearing Potential:

1. **Surgically sterile:** Females who are permanently sterile via hysterectomy, bilateral salpingectomy, and/or bilateral oophorectomy by reported medical history and/or medical records. Surgical sterilisation to have occurred a minimum of 6 weeks, or at the Investigator's discretion, prior to Screening.
2. **Postmenopausal:** Females with amenorrhoea for 12 months without an alternative medical reason with confirmatory follicle-stimulating hormone (FSH) levels of ≥40 mIU/mL. The amenorrhoea should not be induced by a medical condition such as anorexia nervosa, hypothyroid disease, or polycystic ovarian disease, or by extreme exercise. It should not be due to concomitant medications that may have induced the amenorrhoea such as oral contraceptives, hormones, gonadotropin-releasing hormones, anti-oestrogens, or selective oestrogen receptor modulators. Women aged >60 years old whose FSH values are not ≥40 mIU/L may be included at the discretion of the Investigator and in consultation with the Sponsor.

Fertile male: A male that is considered fertile after puberty.

Infertile male: Permanently sterile male via bilateral orchiectomy.

Contraception Guidance

Female Subjects

Female subjects who are of nonchildbearing potential will not be required to use contraception. Female subjects of childbearing potential must be willing to use 2 methods (1 primary and 1 secondary method) of birth control from the time of signing the Informed Consent Form (ICF) until 90 days after the Follow-up visit. Primary (nonbarrier) methods of contraception that are acceptable for all female subjects include:

- surgical method performed at least 3 months prior to the Screening visit:
  - bilateral tubal ligation
  - Essure® (hysteroscopic bilateral tubal occlusion) with confirmation of occlusion of the fallopian tubes
- nonhormonal intrauterine device
- vasectomised male partner (sterilisation performed at least 90 days prior to the Screening visit, with verbal confirmation of surgical success, and the sole partner for the female subject).

For Part C, the following additional primary (nonbarrier) methods of contraception are also acceptable:

- hormonal injection (as prescribed)
- combined oral contraceptive pill or progestin/progestogen-only pill (as prescribed)
- combined hormonal patch (as prescribed)
- combined hormonal vaginal ring (as prescribed)
- hormonal implant
- hormonal intrauterine device.

Secondary (barrier) methods of contraception that are acceptable for all female subjects include:

- male condom with spermicide
- female condom with spermicide
- over-the-counter sponge with spermicide
- cervical cap with spermicide (with appropriate regimen of every 2 years)
- diaphragm with spermicide (with appropriate regimen of every 2 years).

Female subjects of childbearing potential should refrain from donation of ova from Check-in until 90 days after the Follow-up visit.

Male Subjects

Male subjects (even with a history of vasectomy) with partners of childbearing potential must use a male barrier method of contraception (ie, male condom with spermicide) in addition to a second method of acceptable contraception from Check-in until 90 days after the Follow-up visit. Acceptable methods of contraception include:

- hormonal injection
- combined oral contraceptive pill or progestin/progestogen-only pill
- combined hormonal patch
- combined hormonal vaginal ring
- surgical method (bilateral tubal ligation or Essure [hysteroscopic bilateral tubal occlusion])
- hormonal implant
- hormonal or nonhormonal intrauterine device
- over-the-counter sponge with spermicide
- cervical cap with spermicide
- diaphragm with spermicide

- vasectomised male subject (sterilisation performed at least 90 days prior to the Screening visit, with verbal confirmation of surgical success).

For male subjects (even with a history of vasectomy), sexual intercourse with female partners who are pregnant or breastfeeding should be avoided unless condoms are used from the time of the first dose until 90 days after the Follow-up visit. Male subjects are required to refrain from donation of sperm from Check-in until 90 days after the Follow-up visit.

Sexual Abstinence and Same-sex Relationships

Subjects who practice true abstinence, because of the subject's lifestyle choice (ie, the subject should not become abstinent just for the purpose of study participation), are exempt from contraceptive requirements. Periodic abstinence (eg, calendar, ovulation, symptothermal, postovulation methods) and withdrawal are not acceptable methods of contraception. If a subject who is abstinent at the time of signing the ICF becomes sexually active, they must agree to use contraception as described previously.

For subjects who are exclusively in same-sex relationships, contraceptive requirements do not apply. If a subject who is in a same-sex relationship at the time of signing the ICF becomes engaged in a heterosexual relationship, they must agree to use contraception as described previously.

Appendix 4: Regulatory, Ethical, and Study Oversight Considerations

Regulatory and Ethical Considerations

This study will be conducted in accordance with the protocol and with the following:

- Consensus ethical principles derived from international guidelines including the Declaration of Helsinki and Council for International Organizations of Medical Sciences International Ethical Guidelines
- Applicable International Conference on Harmonisation (ICH) Good Clinical Practice (GCP) Guidelines
- Applicable laws and regulations.

The protocol, protocol amendments, Informed Consent Form (ICF), Investigator's Brochure, and other relevant documents must be submitted to an Ethics Committee (EC) by the Investigator and be reviewed and approved by the EC before the study is initiated.

Any substantial protocol amendments, likely to affect the safety of the subjects or the conduct of the study, will require EC and regulatory authority (as locally required) approval before implementation of changes made to the study design, except for changes necessary to eliminate an immediate hazard to study subjects or any nonsubstantial changes, as defined by regulatory requirements.

The Investigator will be responsible for the following:

- Providing written summaries of the status of the study to the EC annually or more frequently in accordance with the requirements, policies, and procedures established by the EC
- Notifying the EC of serious adverse events, Suspected Unexpected Serious Adverse Reactions, or other significant safety findings as required by EC procedures
- Providing oversight of the conduct of the study at the site and adherence to requirements of 21 Code of Federal Regulations (CFR), ICH guidelines, the EC, European regulation 536/2014 for clinical studies (if applicable), and all other applicable local regulations.

Finances and Insurance

Financing and insurance will be addressed in a separate agreement.

Informed Consent

Prior to starting participation in the study, each subject will be provided with a study-specific ICF giving details of the study drugs, procedures, and potential risks of the study. Subjects will be instructed that they are free to obtain further information from the Investigator (or designee) and that their participation is voluntary and they are free to withdraw from the study at any time. Subjects will be given an opportunity to ask questions about the study prior to providing consent for participation.

Following discussion of the study with Clinical Research Unit personnel, subjects will sign 2 copies of the ICF in the presence of a suitably trained member of staff to indicate that they are freely giving their informed consent. One copy will be given to the subject, and the other will be maintained in the subject's records.

Subjects must be reconsented to the most current version of the ICF(s) during their participation in the study.

Subject Data Protection

Subjects will be assigned a unique identifier and will not be identified by name in electronic Case Report Forms (eCRFs), study-related forms, study reports, or any related publications. Subject and Investigator personal data will be treated in compliance with all applicable laws and regulations. In the event the study protocol, study report, or study data are included in a public registry, all identifiable information from individual subjects or Investigators will be redacted according to applicable laws and regulations.

The subject must be informed that his/her personal study-related data will be used by the Sponsor in accordance with European Union General Data Protection Regulation and local data protection law. The level of disclosure must also be explained to the subject. The subject must also be informed that his/her study-related data may be examined by Sponsor or Contract Research Organisation (CRO) auditors or other authorised personnel appointed by the Sponsor, by appropriate EC members, and by inspectors from regulatory authorities.

Disclosure

All information provided regarding the study, as well as all information collected and/or documented during the course of the study, will be regarded as confidential. The Investigator (or designee) agrees not to disclose such information in any way without prior written permission from the Sponsor.

Data Quality Assurance

The following data quality steps will be implemented:

- All relevant subject data relating to the study will be recorded on eCRFs unless directly transmitted to the Sponsor or designee electronically (eg, laboratory data). The Investigator is responsible for verifying that data entries are accurate and correct by electronically signing the eCRF.
- The Investigator must maintain accurate documentation (source data) that supports the information entered in the eCRF.
- The Investigator must permit study-related monitoring, audits, EC review, and regulatory agency inspections and provide direct access to source data documents.
- Covance is responsible for the data management of this study including quality checking of the data. Predefined agreed risks, monitoring thresholds, quality tolerance thresholds, controls, and mitigation plans will be documented in a risk management register. Additional details of quality checking to be performed on the data may be included in a Data Management Plan.

- A Study Monitor will perform ongoing source data verification to confirm that data entered into the eCRF by authorised site personnel are accurate, complete, and verifiable from source documents; that the safety and rights of subjects are being protected; and that the study is being conducted in accordance with the currently approved protocol and any other study agreements, ICH GCP, and all applicable regulatory requirements.
- Records and documents, including signed ICFs, pertaining to the conduct of this study must be retained by the Investigator in the study site archive for at least 5 years after the end of the study unless local regulations or institutional policies require a longer retention period. No records may be destroyed during the retention period without the written approval of the Sponsor. No records may be transferred to another location or party without written notification to the Sponsor.

Investigator Documentation Responsibilities

All individual, subject-specific study data will also be entered into a 21 CFR Part 11-compliant electronic data capture (EDC) system on an eCRF in a timely fashion.

All data generated from external sources (eg, laboratory and bioanalytical data), and transmitted to Covance electronically, will be integrated with the subject's eCRF data in accordance with the Data Management Plan.

An eCRF must be completed for each enrolled subject who undergoes any screening procedures, according to the eCRF completion instructions. The Sponsor, or CRO, will review the supporting source documentation against the data entered into the eCRFs to verify the accuracy of the electronic data. The Investigator will ensure that corrections are made to the eCRFs and that data queries are resolved in a timely fashion by the study staff.

The Investigator will sign and date the eCRF via the EDC system's electronic signature procedure. These signatures will indicate that the Investigator reviewed and approved the data on the eCRF, data queries, and site notifications.

Publications

The results of this study will be submitted for publication.

The primary publication based on this study must be published before any secondary publications are submitted for publication. Authors of the primary publication must fulfil the criteria defined by the International Committee of Medical Journal Editors.[13]

Appendix 5: Schedule of Assessments

Table 1: Schedule of Assessments – Part A

Study Procedures	Screening (Days -28 to -2)	Treatment Periods 1 and 2[a]			Early Termination	Follow-up (Day 6)
		Day -1	Days 1 to 3	Days 4 and 5		
Informed consent	X					
Inclusion/exclusion criteria	X	X				
Demographic data	X					
Medical history	X	X[b]				
Urinary drug screen	X	X				
Alcohol breath test	X	X				
Serology	X					
Genotyping blood sample		X				
Pregnancy test[c]	X	X			X	X
Follicle-stimulating hormone	X[d]					
Height and body weight	X[e]	X	Day 3		X	
**Study residency:**						
Check-in		X				
Check-out			Day 3			
Nonresidential visit	X			X	X	X
**Study drug administration:**						
KL1333 or placebo			Day 1 (0 hour; 30 minutes after starting a high-fat breakfast in Treatment Period 2)[f]			
**Pharmacokinetics:**						
Blood sampling			Predose and 0.25, 0.5, 1, 2, 3, 4, 6, 8, 10, 12, 24, 36, and 48 hours postdose	72 and 96 hours postdose	X	120 hours postdose

Table 1: Schedule of Assessments – Part A

Study Procedures	Screening (Days -28 to -2)	Treatment Periods 1 and 2[a]			Early Termination	Follow-up (Day 6)
		Day -1	Days 1 to 3	Days 4 and 5		
**Safety and tolerability:**						
Adverse event recording	X	X	Ongoing	X	X	X
Prior/concomitant medication monitoring	X	X	Ongoing	X	X	X
Clinical laboratory evaluations[g]	X	X	Day 3		X	X
Blood pressure, pulse rate, and body temperature[h]	X	X	Predose and 2, 4, 12, 24, and 48 hours postdose	X	X	X
Safety 12-lead ECG	X	X[i]	1, 24, and 48 hours postdose		X	X
Continuous 12-lead ECG			From 1 hour predose to 24 hours postdose			
Continuous 12-lead ECG extraction			45, 30, and 15 minutes predose and 0.25, 0.5, 1, 2, 3, 4, 6, 8, 10, 12, and 24 hours postdose			
Complete physical examination	X[j]				X	X
Symptom-directed physical examination			Prior to discharge on Day 3			

Abbreviations: ECG = electrocardiogram.
[a] There will be at least a 10-day washout between KL1333 doses (from Period 1, Day 1 to Period 2, Day 1) for Cohort A1. If additional cohorts are enrolled in Part A, these cohorts will participate in a single treatment period.
[b] Interim medical history. Treatment Period 1 only.
[c] In all females. Performed in serum at Screening and in urine at all other times. A positive urine pregnancy test will be confirmed with a serum pregnancy test.
[d] In postmenopausal females.
[e] Height measured at Screening only.
[f] If additional cohorts are enrolled in Part A, the dietary status will be determined following review of Group A1 results.

[g] Clinical chemistry, haematology, and urinalysis. Subjects will be fasted at least 8 hours.

[h] Supine and standing blood pressure and pulse rate. Day 1 predose blood pressure and pulse rate will be measured in triplicate.

[i] Measured in triplicate.

[j] Performed between the Screening visit and predose assessments.

Table 2: Schedule of Assessments – Part B

Study Procedures	Screening (Days -28 to -2)	Day -1	Days 1 to 12	Days 13 and 14	Early Termination	Follow-up (Day 15)
Informed consent	X					
Inclusion/exclusion criteria	X	X				
Demographic data	X					
Medical history	X	X[a]				
Urinary drug screen	X	X				
Alcohol breath test	X	X				
Serology	X					
Genotyping blood sample		X				
Pregnancy test[b]	X	X			X	X
Follicle-stimulating hormone	X[c]					
Height and body weight	X[d]	X	Day 12		X	
**Study residency:**						
Check-in		X				
Check-out			Day 12			
Nonresidential visit	X			X	X	X
**Study drug administration:**						
KL1333 or placebo			Day 1 to 10 (0 hour)			

Table 2: Schedule of Assessments – Part B

Study Procedures	Screening (Days -28 to -2)	Day -1	Days 1 to 12	Days 13 and 14	Early Termination	Follow-up (Day 15)
**Pharmacokinetics:**						
Blood sampling			Day 1: Predose and 0.25, 0.5, 1, 2, 3, 4, 6, 8, 10, 12, and 24 hours postdose (24-hour postdose sample will be predose on Day 2)<br>Days 3 to 9: Predose<br>Day 10: Predose and 0.25, 0.5, 1, 2, 3, 4, 6, 8, 10, 12, 24, 36, and 48 hours postdose	72 and 96 hours postdose	X	120 hours postdose
**Pharmacodynamics:**						
Blood for biomarkers[e]			Days 1, 2, 4, 8, and 10: Predose<br>Day 11		X	X
Blood for metabolomics[f]			Days 1, 2, 4, 8, and 10: Predose<br>Day 11		X	X
**Safety and tolerability:**						
Adverse event recording	X	X	Ongoing	X	X	X
Prior/concomitant medication monitoring	X	X	Ongoing	X	X	X
Clinical laboratory evaluations[g]	X	X	Day 7: Predose<br>Day 10: Predose and 48 hours postdose		X	X
C-reactive protein, erythrocyte sedimentation rate, and faecal calprotectin[h]		X	Days 4 and 10: Predose			X
Blood pressure, pulse rate, and body temperature[i]	X	X	Day 1: Predose and 2, 4, and 12 hours postdose<br>Days 2 to 10: Predose<br>Days 11 and 12	X	X	X

Table 2: Schedule of Assessments – Part B

Study Procedures	Screening (Days -28 to -2)	Day -1	Days 1 to 12	Days 13 and 14	Early Termination	Follow-up (Day 15)
Safety 12-lead ECG	X	X[j]	Day 1: 2 hours postdose<br>Days 2 to 10: Predose<br>Days 11 and 12		X	X
Continuous 12-lead ECG			Day 1: From 1 hour predose to 24 hours postdose<br>Day 10: From 1 hour predose to 24 hours postdose			
Continuous 12-lead ECG extraction			Day 1: 45, 30, and 15 minutes predose and 0.25, 0.5, 1, 2, 3, 4, 6, 8, 10, 12, and 24 hours postdose (24-hour postdose will be predose on Day 2)<br>Day 10: 45, 30, and 15 minutes predose and 0.25, 0.5, 1, 2, 3, 4, 6, 8, 10, 12, and 24 hours postdose			
Complete physical examination	X[k]				X	X
Symptom-directed physical examination			Days 1 to 10: Predose<br>Days 11 and 12			

Abbreviation: ECG = electrocardiogram.
[a] Interim medical history.
[b] In all females. Performed in serum at Screening and in urine at all other times. A positive urine pregnancy test will be confirmed with a serum pregnancy test.
[c] In postmenopausal females.
[d] Height measured at Screening only.
[e] Biomarkers includes nicotinamide adenine dinucleotide (oxidized form)/nicotinamide adenine dinucleotide (reduced form), pyruvate, lactate, and other biomarkers. Subjects will be fasted at least 8 hours.
[f] Subjects will be fasted at least 8 hours.
[g] Clinical chemistry, haematology, and urinalysis. Subjects will be fasted at least 8 hours.
[h] In Group B5. Up to 3 additional symptom-directed evaluations may be performed based on the Investigator's judgment.
[i] Supine and standing blood pressure and pulse rate. Day 1 predose blood pressure and pulse rate will be measured in triplicate.
[j] Measured in triplicate.
[k] Performed between the Screening visit and predose assessments.

Table 3: Schedule of Assessments – Part C

Study Procedures	Screening (Days -75 to -2)	Day -1	Day 1	Day 2	Day 3	Day 4	Days 5 to 7	Day 8	Day 9	Day 10	Day 11	ET	Follow-up (Day 15)
Informed consent	X												
Inclusion/exclusion criteria	X	X											
Demographic data	X												
Medical history	X	X[a]											
Urinary drug screen	X	X								X			
Alcohol breath test	X	X								X			
Serology	X												
HbA1c	X												
Genotyping blood sample		X											
Pregnancy test[b]	X	X										X	X
Follicle-stimulating hormone	X[c]												
Height and body weight	X[d]	X									X	X	
**Study residency:**													
Check-in		X[e]								X[e]			
Check-out				X[e]							X[e]		
Nonresidential visit	X					X		X				X	X
Clinical site outpatient and diary record					X		X		X				
**Study drug administration:**													
KL1333 or placebo			X	X	X	X	X	X	X	X			

Table 3: Schedule of Assessments – Part C

Study Procedures	Screening (Days -75 to -2)	Day -1	Day 1	Day 2	Day 3	Day 4	Days 5 to 7	Day 8	Day 9	Day 10	Day 11	ET	Follow-up (Day 15)
**Pharmacokinetics:**													
Blood sampling			Predose and 0.25, 0.5, 1, 2, 3, 4, 6, 8, 10, and 12[f] hours postdose	24 hours postdose (predose on Day 2)		X[g]		X[g]		Predose and 0.25, 0.5, 1, 2, 3, 4, 6, 8, 10, and 12[f] hours postdose	24 hours postdose	X	
**Pharmacodynamics:**													
Blood for biomarkers[h]			X[g]	X[g]		X[g]		X[g]		X[g]	X	X	X
Blood for albumin and glycated albumin			X[g]								X	X	X
Blood for metabolomics[i]			X[g]	X[g]		X[g]		X[g]		X[g]	X	X	X
Newcastle Mitochondrial Disease Adult Scale		X								X		X	
Clinical Global Impression		X								X		X	
Patient Global Impression-Improvement		X								X		X	
Daily Fatigue Impact Severity		X	X	X	X	X	X	X	X	X	X	X	
Quality of Life in Neurological Disorders Fatigue Short Form		X								X		X	

Table 3: Schedule of Assessments – Part C

Study Procedures	Screening (Days -75 to -2)	Day -1	Day 1	Day 2	Day 3	Day 4	Days 5 to 7	Day 8	Day 9	Day 10	Day 11	ET	Follow-up (Day 15)
30 Second Sit-to-Stand Test		X								X		X	
**Safety and tolerability:**													
Adverse event recording	X	X	X	X	X	X	X	X	X	X	X	X	X
Prior/concomitant medication monitoring	X	X	X	X	X	X	X	X	X	X	X	X	X
Clinical laboratory evaluations[j]	X	X						X[g]		X[g]	X	X	X
C-reactive protein, erythrocyte sedimentation rate, and faecal calprotectin[k]		X								X[g]			
Blood pressure, pulse rate, and body temperature[l]	X	X	Predose and 2, 4, and 10 hours postdose	X[g]		X[g]		X[g]		X[g]	X	X	X
Safety 12-lead ECG[m]	X	X	2 hours postdose	X[g]		X[g]		X[g]		X[g]	X	X	X
Complete physical examination	X[n]											X	X
Symptom-directed physical examination			X[g]	X[g]		X[g]		X[g]		X[g]	X		
Exit interview											X	X	

Abbreviations: ECG = electrocardiogram; ET = early termination; HbA1c = glycated haemoglobin.
[a] Interim medical history.
[b] In all females. Performed in serum at Screening and in urine at all other times. A positive urine pregnancy test will be confirmed with a serum pregnancy test.

[c] In postmenopausal females.
[d] Height measured at Screening only.
[e] Subjects will reside at or nearby the clinical site after completion of 10-hour postdose procedures.
[f] The 12-hour postdose pharmacokinetic sampling timepoint is optional.
[g] Predose.
[h] Biomarkers includes nicotinamide adenine dinucleotide (oxidized form)/nicotinamide adenine dinucleotide (reduced form), pyruvate, lactate, and other biomarkers. Patients may be fasted when possible, dependent on the Investigator's judgment.
[i] Patients may be fasted when possible, dependent on the Investigator's judgment.
[j] Clinical chemistry, haematology, and urinalysis. Patients may be fasted up to 2 hours when possible, dependent on the Investigator's judgment. Samples should be taken at approximately the same time of day within each patient.
[k] Optional. Up to 3 additional symptom-directed evaluations may be performed based on the Investigator's judgment.
[l] Supine and standing blood pressure and pulse rate. Day 1 predose blood pressure and pulse rate will be measured in triplicate.
[m] Measured in triplicate.
[n] Performed during the Screening period (between the Screening visit and Day -1 Check-in, inclusive).

Table 4: Schedule of Assessments – Part D

Study Procedures	Screening (Days -35 to -2)	Day -1	Days 1 to 12	Days 13 and 14	Early Termination	Follow-up (Day 15)
Informed consent	X					
Inclusion/exclusion criteria	X	X				
Demographic data	X					
Medical history	X	X[a]				
Urinary drug screen	X	X				
Alcohol breath test	X	X				
Serology	X					
Genotyping blood sample		X				
Pregnancy test[b]	X	X			X	X
Follicle-stimulating hormone	X[c]					
Height and body weight	X[d]	X	Day 12		X	
**Study residency:**						
Check-in		X				
Check-out			Day 12			
Nonresidential visit	X			X	X	X
**Study drug administration:**						
KL1333 or placebo			Group D1: Days 1 to 10 (0 and 12 hours) BID with a single dose administration on Day 10<br><br>Group D2: Days 1 to 10 (0, 8, and 16 hours) TID with a single dose administration on Day 10			

Table 4: Schedule of Assessments – Part D

Study Procedures	Screening (Days -35 to -2)	Day -1	Days 1 to 12	Days 13 and 14	Early Termination	Follow-up (Day 15)
**Pharmacokinetics:**						
Blood sampling			Group D1:<br>Day 1: Prior to first dose and 0.25, 0.5, 1, 2, 3, 4, 6, 8, 10, and 12 hours post first dose (12-hour sample will be prior to second dose)<br>Days 2 to 9: Prior to first dose<br>Day 10: Predose and 0.25, 0.5, 1, 2, 3, 4, 6, 8, 10, 12, 24, 36, and 48 hours postdose<br><br>Group D2:<br>Day 1: Prior to first dose and 0.25, 0.5, 1, 2, 3, 4, 6, and 8 hours post first dose (8-hour sample will be prior to second dose)<br>Days 3 to 9: Prior to first dose<br>Day 10: Predose and 0.25, 0.5, 1, 2, 3, 4, 6, 8, 10, 12, 24, 36, and 48 hours postdose	72 and 96 hours postdose	X	120 hours postdose
**Pharmacodynamics:**						
Blood for biomarkers[e]			Day 1: Prior to first dose and 0.5 and 2 hours post first dose<br>Day 10: Predose and 0.5 and 2 hours postdose		X	X
Blood for metabolomics[f]			Day 1: Prior to first dose and 0.5 and 2 hours post first dose<br>Day 10: Predose and 0.5 and 2 hours postdose		X	X
**Safety and tolerability:**						
Adverse event recording	X	X	Ongoing	X	X	X

Table 4: Schedule of Assessments – Part D

Study Procedures	Screening (Days -35 to -2)	Day -1	Days 1 to 12	Days 13 and 14	Early Termination	Follow-up (Day 15)
Prior/concomitant medication monitoring	X	X	Ongoing	X	X	X
Clinical laboratory evaluations[g]	X	X	Day 7: Prior to first dose<br>Day 10: Predose and 48 hours postdose		X	X
C-reactive protein, erythrocyte sedimentation rate, and faecal calprotectin[h]		X	Day 4: Prior to first dose<br>Day 10: Predose			X
Blood pressure, pulse rate, and body temperature[i]	X	X	Day 1: Prior to first dose and 2, 4, and 12 hours post first dose (12-hour sample will be prior to second dose in Group D1)<br>Days 2 to 9: Prior to first dose<br>Day 10: Predose<br>Days 11 and 12	X	X	X
Safety 12-lead ECG	X	X[j]	Day 1: 2 hours post first dose<br>Days 2 to 9: Prior to first dose<br>Day 10: Predose<br>Days 11 and 12		X	X
Complete physical examination	X[k]				X	X
Symptom-directed physical examination			Days 1 to 9: Prior to first dose<br>Day 10: Predose<br>Days 11 and 12			

Abbreviations: BID = twice daily; ECG = electrocardiogram; TID = 3 times daily.
[a] Interim medical history.
[b] In all females. Performed in serum at Screening and in urine at all other times. A positive urine pregnancy test will be confirmed with a serum pregnancy test.
[c] In postmenopausal females.
[d] Height measured at Screening only.
[e] Biomarkers includes nicotinamide adenine dinucleotide (oxidized form)/nicotinamide adenine dinucleotide (reduced form), pyruvate, lactate, and other biomarkers.
[f] Blood for metabolomics will include a whole blood sample and a serum sample at each timepoint.

[g] Clinical chemistry, haematology, and urinalysis. Subjects will be fasted at least 8 hours.
[h] Up to 3 additional symptom-directed evaluations may be performed based on the Investigator’s judgment.
[i] Supine and standing blood pressure and pulse rate. Blood pressure and pulse rate prior to the first dose on Day 1 will be measured in triplicate.
[j] Measured in triplicate.
[k] Performed between the Screening visit and assessments prior to the first dose.
