# Supplementary material for: Optimizing rare disorder trials: a phase 1a/1b randomized study of KL1333 in adults with mitochondrial disease
Source: Brain. 2024 Dec 9;148(1):39–46. doi: 10.1093/brain/awae308 (PMC11706290; doi:10.1093/brain/awae308)
Supplement: awae308_Supplementary_Data [file awae308_supplementary_data.zip › SupplementaryFigures.pdf]

Supplementary Table 1. Study design and treatment duration in healthy volunteers divided in 8 cohorts (Part A, B & D) and one cohort of patients with genetically confirmed PMD (Part C)

Part	Study population	Study Treatment	Treatment duration
A	Healthy volunteers	25 mg KL1333 (n=6) or placebo (n=2)	Single doses fasted and fed
B	Healthy volunteers	25 mg KL1333 (n=6) or placebo (n=2) QD	10 days (fasted)
		50 mg KL1333 (n=6) or placebo (n=2) QD	
		75 mg KL1333 (n=6) or placebo (n=2) QD	
		150 mg KL1333 (n=6) or placebo (n=2) QD	
		250 mg KL1333 (n=6) or placebo (n=2) QD	
C	Patients with PMD	50 mg KL1333 (n=6) or placebo (n=2) QD	10 days (no requirement for fasting)
D	Healthy volunteers	75 mg KL1333 (n=6) or placebo (n=2) BID	10 days (fasted at day 1, 7, and 10)
		50 mg KL1333 (n=6) or placebo (n=2) TID	

PMD = Primary Mitochondrial Disease; QD = once daily; BID = twice daily; TID = three times daily

Supplementary Table 2. Adverse Events (AE) (all causalities) across study cohorts and level of severity

	Part A				Part B						Part C		Part D		
	**Placebo**		**KL1333**		**Placebo**	**KL1333**					**Placebo**	**KL1333**	**Placebo**	**KL1333**	
	**Fasted (N=2)**	**Fed (N=2)**	**25 mg Fasted (N=6)**	**25 mg Fed (N=6)**	**QD (N=10)**	**25 mg QD (N=6)**	**50 mg QD (N=6)**	**75 mg QD (N=6)**	**150 mg QD (N=6)**	**250 mg QD (N=6)**	**QD (N=2)**	**50 mg QD (N=6)**	**BID/TID (N=4)**	**75 mg BID (N=6)**	**50 mg TID (N=6)**
Treatment Emergent AEs															
Overall	1 (50.0%) [3]	2 (100%) [4]	2 (33.3%) [3]	2 (33.3%) [2]	2 (20.0%) [5]	1 (16.7%) [2]	3 (50.0%) [13]	6 (100%) [14]	4 (66.7%) [28]	6 (100%) [30]	1 (50.0%) [7]	6 (100%) [19]	3 (75.0%) [7]	4 (66.7%) [14]	3 (50.0%) [6]
Serious (SAE)	---	---	---	---	---	---	---	---	---	---	---	---	---	---	---
Leading to Discontinuation	---	---	---	---	---	---	---	---	---	---	---	---	---	---	---
Leading to Death	---	---	---	---	---	---	---	---	---	---	---	---	---	---	---
Intensity															
Mild	1 (50.0%) [3]	2 (100%) [4]	2 (33.3%) [3]	2 (33.3%) [2]	2 (20.0%) [5]	1 (16.7%) [2]	3 (50.0%) [12]	6 (100%) [11]	4 (66.7%) [23]	6 (100%) [25]	1 (50.0%) [4]	6 (100%) [17]	3 (75.0%) [7]	4 (66.7%) [11]	3 (50.0%) [6]
Moderate	---	---	---	---	---	---	1 (16.7%) [1]	1 (16.7%) [3]	2 (33.3%) [5]	3 (50.0%) [5]	1 (50.0%) [3]	1 (16.7%) [1]	---	1 (16.7%) [3]	---
Severe	---	---	---	---	---	---	---	---	---	---	---	1 (16.7%) [1]	---	---	---

Number of subjects with an adverse event (percentage of subjects with an adverse event) and [number of adverse events]. QD once daily; BID twice daily; TID three times daily

Supplementary Table 3. Adverse Events (AE) (all causalities) occurring in at least 3 subjects, according to System Organ Class and Preferred Term using Medical Dictionary for Regulatory Activities (MedDRA), version 21.1

	**Part A**				**Part B**						**Part C**		**Part D**		
	**Placebo**		**KL1333**		**Placebo**	**KL1333**					**Placebo**	**KL1333**	**Placebo**	**KL1333**	
	**Fasted (N=2)**	**Fed (N=2)**	**25 mg Fasted (N=6)**	**25 mg Fed (N=6)**	**QD (N=10)**	**25 mg QD (N=6)**	**50 mg QD (N=6)**	**75 mg QD (N=6)**	**150 mg QD (N=6)**	**250 mg QD (N=6)**	**QD (N=2)**	**50 mg QD (N=6)**	**BID/ TID (N=4)**	**75 mg BID (N=6)**	**50 mg TID (N=6)**
Gastrointestinal disorders															
Abdominal pain upper	---	---	---	---	---	---	2 (33.3%) [2]	3 (50.0%) [3]	4 (66.7%) [4]	5 (83.3%) [5]	---	---	1 (25.0%) [1]	3 (50.0%) [3]	2 (33.3%) [2]
Diarrhoea	---	---	1 (16.7%) [2]	1 (16.7%) [2]	---	---	1 (16.7%) [1]	2 (33.3%) [2]	3 (50.0%) [3]	6 (100%) [6]	---	1 (16.7%) [1]	2 (50.0%) [2]	3 (50.0%) [3]	1 (16.7%) [1]
Nausea	1 (50.0%) [2]	1 (50.0%) [2]	---	---	1 (10.0%) [1]	---	1 (16.7%) [1]	1 (16.7%) [1]	3 (50.0%) [3]	2 (33.3%) [2]	---	2 (33.3%) [2]	1 (25.0%) [1]	---	1 (16.7%) [1]
Abdominal pain lower	---	---	---	---	1 (10.0%) [1]	---	---	1 (16.7%) [1]	2 (33.3%) [2]	---	---	---	1 (25.0%) [1]	---	---
Constipation	---	---	---	---	1 (10.0%) [1]	1 (16.7%) [1]	1 (16.7%) [1]	---	---	1 (16.7%) [1]	---	---	---	---	---
Dyspepsia	---	---	---	---	---	---	---	1 (16.7%) [1]	1 (16.7%) [1]	1 (16.7%) [1]	---	---	---	---	---
Mouth ulceration	---	---	---	---	---	---	1 (16.7%) [1]	1 (16.7%) [1]	---	1 (16.7%) [1]	---	---	---	---	---

Nervous system disorders															
Headache	---	---	---	---	---	---	3 (50.0%) [3]	1 (16.7%) [1]	3 (50.0%) [3]	2 (33.3%) [2]	1 (50.0%) [1]	1 (16.7%) [1]	2 (50.0%) [2]	---	---
Dizziness	1 (50.0%)	---	---	---	---	---	1 (16.7%) [1]	1 (16.7%) [1]	2 (33.3%) [2]	1 (16.7%) [1]	1 (50.0%) [1]	---	---	---	---
General disorders and administration site conditions															
Fatigue	---	---	---	---	---	---	---	---	---	1 (16.7%) [1]	1 (50.0%) [1]	2 (33.3%) [2]	---	---	---
Musculoskeletal and connective tissue disorders															
Pain in extremity	---	---	---	---	---	---	---	---	1 (16.7%) [1]	---	1 (50.0%) [1]	1 (16.7%) [1]	---	---	---
Respiratory, thoracic and mediastinal disorders															
Oropharyngeal pain	---	---	---	---	1 (10.0%) [1]	---	---	---	---	---	1 (50.0%) [1]	---	---	1 (16.7%) [1]	---

Number of subjects with an adverse event (percentage of subjects with an adverse event) and [number of adverse events]

Supplementary Table 4. Baseline biomarker values in healthy volunteers (Part B and D, combined) and subjects with PMD (Part C)

	**Healthy volunteers (N=56)**<br>Median (95% CI)	**PMD subjects (N=8)**<br>Median (95% CI)	**Median difference (90% CI)**
**Lactate (mmol/L)**	0.4390 (0.4170, 0.5664)	1.112 (0.5741, 1.656)	**0.540 (0.1790, 1.063)**
**Pyruvate (mmol/L)**	0.03900 (0.03149, 0.04158)	0.03200 (0.02092, 0.05883)	0.001500 (-0.01000, 0.1400)
**Lactate/Pyruvate Ratio**	11.96 (14.08, 26.72)	22.62 (13.87, 49.93)	**10.61 (5.374, 16.53)**
**NAD^+ (pmol/µL)**	3.654 (3.922, 7.143)	3.527 (1.969, 6.566)	0.0095 (-3.009, 1.995)
**NADH (pmol/µL)**	3.603 (4.502, 8.244)	3.824 (1.761, 6.188)	-0.3350 (-3.878, 1.808)
**NAD^+/NADH Ratio**	0.9855 (1.194, 2.058)	1.375 (0.6520, 2.310)	0.1015 (-0.5100, 0.8930)
**FGF21 (ng/L)**	125.3 (125.8, 199.9)[§]	251.1 (117.3, 568.8)	**86.72 (17.66, 247.0)**
**GDF15 (ng/L)**	384.4 (369.5, 486.6)[§]	2344 (1112, 3594)	**1820 (934.7, 2546)**

CI Confidence interval; NAD+ Nicotinamide adenine dinucleotide oxidised form; NADH Nicotinamide adenine dinucleotide reduced form; FGF21 Fibroblast growth factor 21; GDF15 Growth Differentiation Factor 15; PMD Primary mitochondrial disease. [§]Only measured in healthy volunteers in Part B (N=40). Bold font indicates 90% CI for the Hodges-Lehmann estimate of median difference from the Wilcoxon rank sum test was fully above zero.

Supplementary Table 5. Biomarker values at baseline (day 1) and day 10.

		Part B		Part C (PMD)		Part D	
	**Day**	**Placebo (N=10)**	**KL1333 (N=30)**	**Placebo (N=2)**	**KL1333 (N=6)**	**Placebo (N=4)**	**KL1333 (N=12)**
**Lactate (mmol/L)**	1	0.377 (0.222); 0.307	0.444 (0.213); 0.417	0.605 (0.24); 0.605	1.29 (0.66); 1.49	0.553 (0.0284); 0.549	0.686 (0.41); 0.524
	10	0.584 (0.196); 0.509	0.517 (0.411); 0.439	0.799 (0.113); 0.799	1.18 (0.583); 1.21	0.549 (0.146); 0.538	0.628 (0.405); 0.424
**Pyruvate (mmol/L)**	1	0.0406 (0.0249); 0.0275	0.0384 (0.0168); 0.0415	0.036 (0.0184); 0.036	0.0412 (0.0254); 0.0320	0.0325 (0.0152); 0.0335	0.0298 (0.0194); 0.0295
	10	0.0351 (0.0204); 0.0290	0.0361 (0.0197); 0.0360	0.0235 (0.0191); 0.0235	0.0658 (0.0485); 0.0595	0.031 (0.0127); 0.0315	0.031 (0.0128); 0.0300
**Lactate/Pyruvate Ratio**	1	12.4 (10.9); 10.1	16.1 (16.4); 10.2	17.5 (2.36); 17.5	36.7 (23.2); 25.6	20.6 (10.8); 18.3	37.8 (38.9); 30.1
	10	22.5 (14.2); 19.1	23.9 (27.2); 11.5	49.5 (35.9); 49.5	30.6 (33); 20.3	20.4 (12); 15.04	22 (12.2); 16.8
**NAD^+ (pmol/µL)**	1	6.66 (5.23); 6.59	6.8 (7.13); 6.51	2.64 (1.5); 2.64	4.81 (2.95); 4.84	1.31 (0.606); 1.32	2.83 (2.09); 2.14
	10	8.76 (10.5); 4.71	6.76 (8.25); 3.25	5.26 (5.67); 5.26	4.52 (2.87); 4.48	6.81 (4.32); 6.31	9.81 (4.38); 11.2
**NADH (pmol/µL)**	1	8.67 (9.2); 5.07	7.99 (7.01); 7.27	2.57 (2.92); 2.57	4.44 (2.66); 4.01	1.85 (1.64); 1.67	1.91 (1.87); 0.897
	10	4.41 (3.67); 4.02	3.74 (3.84); 1.78	1.62 (1.51); 1.62	4.19 (2.06); 4.62	4.23 (1.72); 4.89	6.17 (2.08); 6.15
**NAD^+/NADH Ratio**	1	1.39 (1.23); 1.02	1.44 (1.49); 0.782	1.94 (1.62); 1.94	1.33 (0.858); 1.38	1.79 (2.43); 0.628	2.23 (1.94); 1.36
	10	3.7 (6.48); 1.72	2.75 (2.5); 2.25	2.84 (0.848); 2.84	1.23 (0.841); 1.06	1.53 (0.526); 1.33	1.58 (0.642); 1.42
**FGF21 (ng/L)**	1	142 (111); 99.3	165 (118); 132	230 (130); 230	381 (303); 274	N/A	N/A
	10	156 (176); 71.9	190 (134); 184	223 (63.9); 223	388 (290); 291	N/A	N/A
**GDF15 (ng/L)**	1	443 (206); 391	423 (178); 378	2050 (1040); 2050	2450 (1680); 2430	N/A	N/A
	10	416 (160); 393	473 (229); 393	1940 (1140); 1940	2580 (1600); 2700	N/A	N/A

Data is displayed as Mean (SD); Median. NAD^+: Nicotinamide adenine dinucleotide oxidized form; NADH: Nicotinamide adenine dinucleotide reduced form; FGF21: Fibroblast growth factor 21; GDF15: Growth Differentiation Factor 15

Supplementary Table 6. Summary of Clinical Outcomes Assessments in subjects with PMD (Part C) at day-1 and day 10

		Timepoint	**Placebo QD (N=2)** Mean (SD); Median	**50 mg KL1333 QD (N=6)** Mean (SD); Median
**Quality of Life in Neurological Disorders Fatigue (Neuro-QoL Fatigue)**	Raw total score	Day -1	18.0 (2.83); 18.0	26.5 (4.81); 26.0
		Day 10	19.5 (2.12); 19.5	24.2 (5.91); 24.5
		LS Means difference day -1 to 10 (90% CI)	1.50 (-2.69, 5.69)	-2.33 (-4.75, 0.0866)
	Standardised total T-score	Day -1	45.6 (2.55); 45.6	54.0 (5.24); 53.3
		Day 10	47.0 (1.98); 47.0	51.7 (5.92); 51.8
		LS Means difference day -1 to 10 (90% CI)	1.40 (-2.65, 5.45)	**-2.35 (-4.69, -0.00976)**
**30 Second Sit to Stand (30s STS) Test**	Repetitions	Day -1	12.5 (0.71); 12.5	12.200 (4.66); 13.0*
		Day 10	13.0 (2.83); 13.0	14.40 (4.83); 15.0*
		LS Means difference day -1 to 10 (90% CI)	0.500 (-2.09, 3.09)	**2.200 (0.5238, 3.876)***
**Clinical Global Impression**	Severity of Illness	Day -1	3.0 (1.41); 3.0	3.7 (0.82); 3.5
		Day 10	3.0 (1.41); 3.0	3.7 (0.82); 3.5
		Median difference day -1 to 10 (90% CI)	0 (0, 0)	0 (0, 0)
	Improvement	Day 10	3.5 (0.71); 3.5	4.2 (0.75); 4.0
**Patient Global Impression**	Severity of Illness	Day -1	2.5 (0.71); 2.5	3.0 (0.63); 3.0
		Day 10	2.0 (0); 2.0	2.7 (0.82); 2.5
		Median difference day -1 to 10 (90% CI)	-0.500 (-1.00, 0)	-0.500 (-1.00, 0)
	Improvement	Day 10	3.5 (0.71); 3.5	3.8 (0.41); 4.0

CI = confidence interval, LS = least squares. LS Means differences from mixed-effects model or Hodges-Lehmann estimate of median difference from the Wilcoxon rank sum test with. Bold font indicates 90% CI for the difference does not contain zero. *One participant with advanced myopathy was unable to perform the 30s STS test; N=5

Supplementary Table 7. Key pharmacokinetic (PK) parameters across the study cohorts.

Parameter	Geometric mean (%CV)									
	Part A		Part B					Part C	Part D	
	25 mg<br>N=6	25 mg<br>N=6	25 mg<br>QD<br>N=6	50 mg<br>QD<br>N=6	75 mg<br>QD<br>N=6	150 mg<br>QD<br>N=6	250 mg<br>QD<br>N=6	50 mg<br>QD<br>N=6	75 mg<br>BID<br>N=6	50 mg<br>TID<br>N=6
	fasted	fed	Day 1,<br>Day 10	Day 1,<br>Day 10	Day 1,<br>Day 10	Day 1,<br>Day 10	Day 1,<br>Day 10	Day 1,<br>Day 10	Day 1,<br>Day 10	Day 1,<br>Day 10
**AUC_{0-24} [A,D]/ $AUC_{0-\tau}$ [B,C] (ng•h/mL)**	1040 (54.1)	818 (66.1)	1070 (35.8)<br>2670 (36.1)	2190 (8.9)<br>4100 (22.4)	2820 (34.8)<br>4530 (48.0)	6210 (39.1)<br>8900 (28.4)	6510 (24.0)<br>7050 (28.9)	1400 (53.1)<br>4150 (62.4)	NA<br>5130 (9.7)	NA<br>6580 (31.8)
**C_{max} (ng/mL)**	180 (67.8)	81.1 (54.6)	210 (30.3)<br>256 (34.2)	387 (28.2)<br>474 (18.1)	386 (39.1)<br>578 (49.5)	1220 (55.8)<br>928 (50.9)	954 (33.1)<br>1440 (17.0)	210 (89.1)<br>334 (72.6)	397 (41.4)<br>570 (39.1)	304 (52.5)<br>475 (37.4)
**C_{trough} (n/mL)**	NA	NA	44.2 (45.2)<br>116 (48.7)	87.9 (16.2)<br>144 (70.0)	92.8 (32.2)<br>154 (57.5)	NC<br>302 (22.3)	210 (30.0)<br>150 (85.7)	56.4 (39.2)<br>161 (81.3)	84.4 (26.9)<br>120 (43.4)	44.9 (88.6)<br>159 (37.1)
**T_{max} (h)[a]**	0.500 (0.500-2.02)	2.04 (1.02-10.0)	0.767 (0.500-4.02)<br>0.758 (0.500-2.02)	0.500 (0.500-0.500)<br>0.758 (0.500-1.02)	1.02 (0.500-4.05)<br>1.02 (0.500-1.02)	1.02 (1.02-2.02)<br>3.02 (0.500-3.02)	1.02 (1.02-2.02)<br>1.02 (1.02-3.02)	0.750 (0.283-3.00)<br>1.41 (0.500-23.9)	1.00 (1.00-2.00)<br>1.00 (5.00-2.00)	1.00 (0.500-6.00)<br>1.00 (1.00-6.00)
**$t_{½}$ (h)**	29.6 (69.0)	38.7 (90.4)	NC<br>23.9 (28.9)	NC<br>23.4 (55.9)	NC<br>31.6 (35.8)	NC<br>26.9 (55.1)	NC<br>26.5 (42.8)	NC<br>NC	NC<br>30.1 (41.4)	NC<br>29.5 (56.1)
**RA_{AUC}**	NA	NA	NA<br>2.49 (16.1)	NA<br>1.88 (25.2)	NA<br>1.61 (42.9)	NA<br>1.44 (17.3)	NA<br>1.08 (26.8)	NA<br>2.96 (39.4)	NA<br>1.96 (27.8)	NA<br>3.12 (35.4)

AUC_{0-24} area under the concentration-time curve from time zero to 24 hours postdose; $AUC_{0-\tau}$ = area under the concentration-time curve over a dosing interval (τ); BID twice daily, C_{max} maximum observed plasma concentration; C_{trough} plasma concentration at the end of the dosing interval; CV coefficient of variation; $t_{1/2}$ apparent plasma terminal elimination half-life; N number of subjects; NA not available; NC not calculated; QD once daily; RA_{AUC} = accumulation ratio based upon $AUC_{0-\tau}$; TID three times daily, T_{max} time of the maximum observed plasma concentration. [a] Median (minimum-maximum).

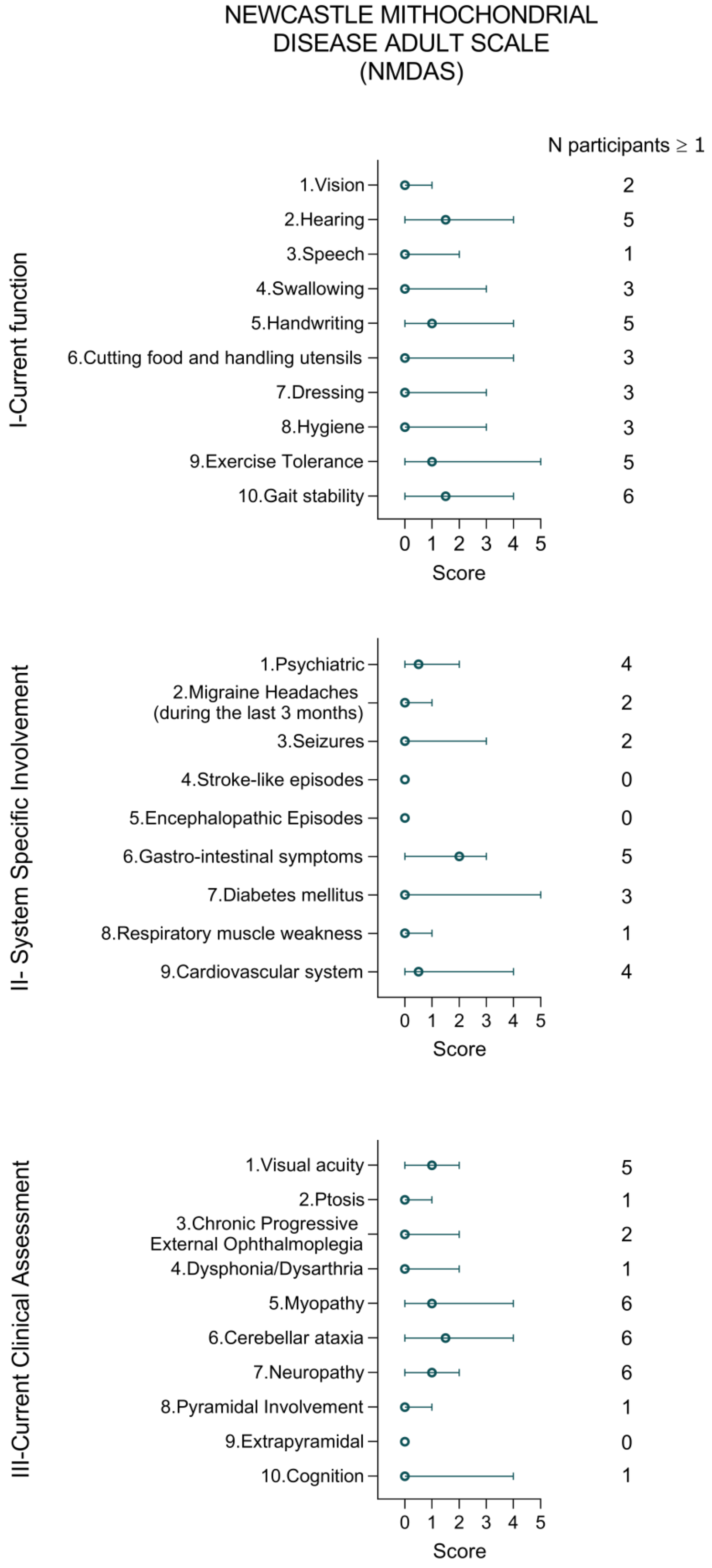
NEWCASTLE MITHOCHONDRIAL
DISEASE ADULT SCALE
(NMDAS)
N participants ≥ 1
I-Current function
1.Vision
2.Hearing
3.Speech
4.Swallowing
5.Handwriting
6.Cutting food and handling utensils
7.Dressing
8.Hygiene
9.Exercise Tolerance
10.Gait stability
2
5
1
3
5
3
3
3
5
6
0 1 2 3 4 5
Score
II- System Specific Involvement
1.Psychiatric
2.Migraine Headaches (during the last 3 months)
3.Seizures
4.Stroke-like episodes
5.Encephalopathic Episodes
6.Gastro-intestinal symptoms
7.Diabetes mellitus
8.Respiratory muscle weakness
9.Cardiovascular system
4
2
2
0
0
5
3
1
4
0 1 2 3 4 5
Score
III-Current Clinical Assessment
1.Visual acuity
2.Ptosis
3.Chronic Progressive External Ophthalmoplegia
4.Dysphonia/Dysarthria
5.Myopathy
6.Cerebellar ataxia
7.Neuropathy
8.Pyramidal Involvement
9.Extrapyramidal
10.Cognition
5
1
2
1
6
6
6
1
0
1
0 1 2 3 4 5
Score

Supplementary Fig. 1 Baseline characteristics of the eight subjects with PMD (Part C) evaluated by the Newcastle Mitochondrial Disease Adult Scale (NMDAS) including current function, system specific involvement and current clinical assessment. Scores are presented as median + range, with higher scores indicative of greater disease severity. Number (N) of participants with PMD with NMDAS subscale score >0 is indicated in the right side of the figure.

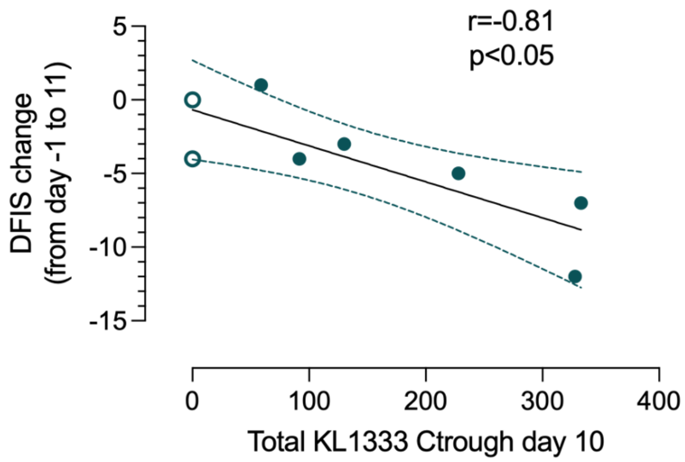

Supplementary Fig. 2 Changes in self-reported Daily Fatigue Impact Scale (DFIS) in patients with PMD. Changes in total DFIS scores from baseline (day -1) to last measure (day 11) plotted against total KL1333 C_{trough} values at steady state (day 10) in participants receiving daily oral doses of 50 mg KL1333 or placebo. Simple linear regressions correlation lines (95% CI) and Pearson correlation coefficients are shown.

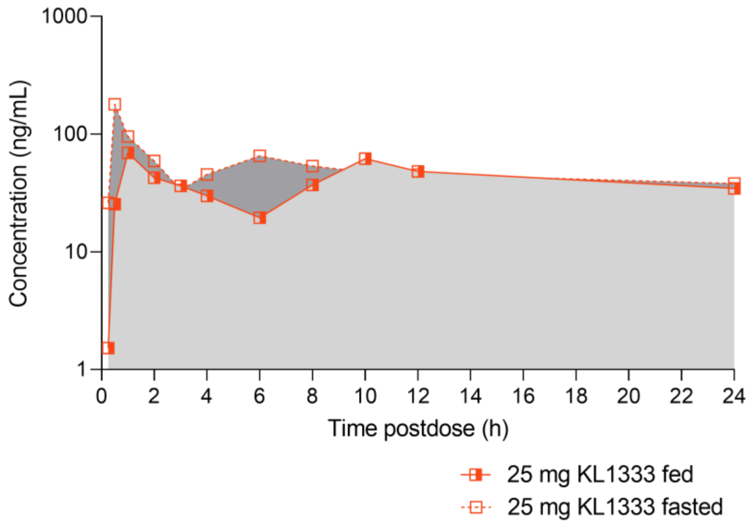

Supplementary Fig. 3 Food effect evaluated by Total KL1333 plasma concentrations. Data are represented as arithmetic mean plasma concentration versus time profiles after an oral 25 mg KL1333 dose in fasted or fed participants (Part A).

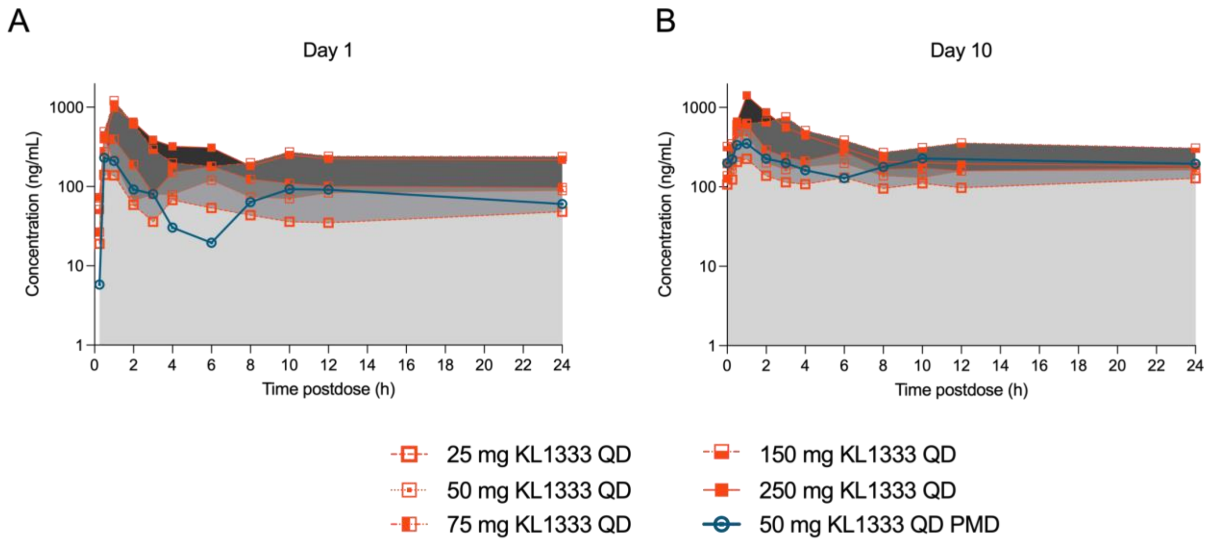

Supplementary Fig. 4 Arithmetic mean plasma concentration versus time profiles of total KL1333 displayed up to 24 hours after treatment initiation on day 1 **(A)** and on day 10 **(B)** following once daily (QD) oral doses ranging from 25 to 250 mg in healthy volunteers and 50 mg in patients with PMD.
