# Supplementary material for: Optimizing rare disorder trials: a phase 1a/1b randomized study of KL1333 in adults with mitochondrial disease
Source: Brain. 2024 Dec 9;148(1):39–46. doi: 10.1093/brain/awae308 (PMC11706290; doi:10.1093/brain/awae308)
Supplement: awae308_Supplementary_Data [file awae308_supplementary_data.zip › SupplementaryText.pdf]

Supplementary material

Rationale

The oxidised and reduced nicotinamide adenine dinucleotide (NAD) forms (NAD^+, NADH) are key factors in energy metabolism, as well as several other cellular processes.[1] Complex I of the mitochondrial electron transport chain (ETC) is an important site for cellular reoxidation of NADH to NAD^+. Impairments of the ETC in PMD patients lead to depletion of NAD^+ and a disrupted NAD^+:NADH ratio, as evidenced by measurements of nucleotide levels in tissues as well as plasma/serum levels of metabolites in redox balance with intracellular NAD^+:NADH, such as lactate:pyruvate ratio.[2-5] The abnormal NAD^+:NADH ratio leads to reductive stress, which impedes NAD^+-dependent energy metabolism and signalling pathways and may contribute to the disease progression.[1, 6-8]

KL1333 (2-isopropyl-3H-naphtho[2,1-d]imidazole-4,5-dione) is a novel, orally available small molecule in therapeutic development for PMD. It is reduced to KL1333H_2 by the widely distributed cytoplasmic flavoprotein NAD(P)H dehydrogenase [quinone]1 (NQO1), using NADH as the electron donor.[9, 10] KL1333H_2 is unstable and is converted back to KL1333 by cytochrome C of the ETC. As a result, KL1333 undergoes multiple redox cycles, with one molecule of NADH being converted to NAD^+ in each cycle. The redox cycles thus function as an alternative way to increase NAD^+ levels. KL1333 has been shown to normalize the NAD^+:NADH ratio in fibroblasts from patients with mitochondrial encephalomyopathy, lactic acidosis, and stroke-like episodes (MELAS) (harbouring m.3243A>G), triggering activation of mitochondrial biogenesis pathways, improving mitochondrial mass and oxidative phosphorylation, and reducing lactate and reactive oxygen species levels.[11]

Supplementary material and methods

Study design

Part A included one cohort, whose healthy participants received a single oral dose of study drug following an overnight fast of at least 8 hours and, following a washout period of at least 10 days, the same treatment after consuming a standard high-fat breakfast. Part B comprised five healthy participant cohorts receiving 25, 50, 75, 150 or 250 mg KL1333 after an overnight fast once daily (QD) for 10 days to achieve steady-state plasma levels of KL1333. Before each cohort in Part B, blinded data from the previous cohort was reviewed in a dose selection

conference meeting before a decision was made about proceeding to the next cohort. Part D contained two healthy volunteer cohorts receiving 75 mg KL1333 twice daily (BID) or 50 mg KL1333 thrice daily (TID) for 10 days with an overnight fast at day 1, 7 and 10. Part C included eight subjects with PMD receiving 50 mg KL1333 QD for 10 days without any requirement for fasting. In Part C, the age and BMI range of PMD subjects was broader that healthy volunteers; the reason for this was to be as inclusive as possible and reflects the reduced BMI of this population.

Study assessments

The condition of each subject was monitored from the time of signing the ICF to final discharge from the study. Subjects were observed for any signs or symptoms and asked about their condition by open questioning. The Investigator provided an assessment of the severity of an adverse event (AE) using the standardized mild, moderate, and severe categories (see Protocol).

Blood samples to assess plasma KL1333 concentrations and evaluate pharmacokinetics (PK) parameters were obtained up to 120 hours after dosing in Parts A, B and D, and up to 24 hours after the final dose in Part C. Predose samples were taken every day in Part B and D, and on day 2, 4, 8 and 10 in Part C. KL1333 and its metabolites (deconjugated KL1333-glucuronide and KL1333-sulphate) were simultaneously measured by liquid chromatography tandem mass spectrometry (LC-MS/MS). Total KL1333 levels were used in the PK analysis, which was conducted using WinNonlin Version 8.1 (Certara, NJ, USA). PK parameters calculated included T_{max}, C_{max}, C_{trough}, area under the curve $(AUC)_{0\text{-}\tau}$, and $AUC_{0\text{-}tlast}$.

To assess pharmacodynamic (PD) biomarkers, additional blood samples were obtained predose in Parts B to D and at 0.5 and 2 hours postdose in Part D, while participants were still fasting. These included lactate and pyruvate concentrations and ratio in whole blood, measured using L-Lactate and Pyruvate Assay Kits (cat no. ab65330 and ab65342; Abcam, Cambridge, UK), and FGF21 and GDF15 concentrations in serum, measured using FGF21 SimpleStep and GDF15 Human ELISA kits (cat no. ab222506 and ab155432; Abcam). Total NAD^+ and NADH concentrations and ratio were evaluated in whole blood using a colorimetric assay based on a cyclic enzymatic reaction in acidic and alkaline extracts, respectively.[12, 13] Biomarker analyses were performed by Complement Genomics Ltd. (Durham, UK).

The Newcastle Mitochondrial Disease Adult Scale (NMDAS), a validated clinical rating scale designed to capture the natural history of mitochondrial disease, was included to assess the

multidimensional spectrum of disease involvement.[14] NMDAS sections I through III include 29 items scored on a 6-point numeric rating scale (NRS) from 0 to 5. Summing the item scores yields a total score ranging from 0 to 145, where higher scores indicate more extensive and severe system involvements. Patient Global Impression (PGI) and Clinician Global Impression (CGI) of disease severity were scored using a 5-point NRS and a 7-point NRS, respectively. PGI and CGI of disease improvement were scored on a 7-point Likert-type scale.

Statistical methods

The frequency of AEs was summarised by treatment and system organ class and preferred term (MedDRA version 21.1 or higher).

To investigate the food effect, natural log (ln) transformed PK parameters were analysed using a mixed model that included treatment received as a fixed effect and participant as a random effect. The least squares (LS) mean for each treatment, difference in LS means between the fed and fasted treatments and corresponding 90% confidence interval (CI) were calculated and back transformed to give the geometric LS mean (GLSM), ratio of GLSMs, and corresponding 90% CI. Dose proportionality of PK parameters was analysed using a power model to calculate a pooled estimate of the slope, corresponding 95% CI, and between participant coefficient of variation.

Supplementary Results

Study population

Within the healthy volunteers, there was a high proportion that was assessed for eligibility but did not enter the study. This finding is not surprising for healthy volunteer Phase 1 studies. Figure 1 provides a high-level overview of the reasons for non-inclusion. About half of those not entering the study did not meet the inclusion criteria. Reasons for screening failure included Gilberts syndrome, ECG abnormalities, blood pressure or BMI out of range, poor venous access, unallowed medication, positive test for recreational drug use, positive hepatitis B, mood disorder. Other common reasons for exclusion were recruitment being already full or subjects not showing up to check in.

In PMD subjects, the baseline NMDAS section I-III total score ranged from 8 to 48. The most frequent symptoms, as evaluated by the NMDAS items, were on gait stability, proximal

myopathy, cerebellar ataxia, neuropathy, each affecting 6 patients (75%), and hearing, exercise tolerance, gastrointestinal symptoms, visual acuity, each affecting five patients (62.5%) (Supplementary Fig. 1).

Safety and tolerability

Across the study, the incidence of AEs ranged from 16.7% to 100% in the different study cohorts for participants exposed to KL1333 and from 20.0% to 100% for participants who received placebo (Supplementary Table 2). In total, 157 AEs were reported, and 104 of those were assessed as at least possibly related to study treatment. Most AEs were of mild (n=135) or moderate (n=21) intensity. There was one severe intensity AE of facial pain occurring concurrently with a mild AE of parosmia in a subject with PMD in Part C, which began on day 1 of dosing of 50 mg KL1333. Dosing for this individual was halted on day 2 and then resumed, and the facial pain and parosmia persisted with varying intensities until day 13.

There were no serious adverse events (SAEs) or participant discontinuation due to AEs.

Pharmacokinetics

KL1333 appeared rapidly in plasma, with a primary peak typically being detected between 0.5- and 3-hours post-dose. The profiles generally showed secondary peaks indicative of enterohepatic recirculation of KL1333. The arithmetic mean plasma concentration versus time profiles of total KL1333 following once-daily oral doses are displayed in Supplementary Fig. 4. Compared to a fasted state, a high fat meal resulted in a later mean T_{max} (2.04 hours versus 0.5 hours) following a single oral dose of 25 mg KL1333 in healthy participants (Supplementary Fig. 3). The GLSM ratio for $AUC_{0-tlast}$ was 0.822, with the 90% CI spanning unity (0.644-1.05), and the GLSM ratio for C_{max} was 0.452 (90% CI 0.353-0.579). In Parts B, C, and D, steady state generally appeared to have been reached by day 6 to 9. The increase in exposure with increasing dose appeared to be slightly subproportional on day 1, with slopes for $AUC_{0-\tau}$ and C_{max} of 0.815 and 0.745, respectively. On day 10, the subproportionality was more marked, with slopes for $AUC_{0-\tau}$ and C_{max} of 0.475 and 0.727, respectively, upper 95% CI bounds that were all <1, and no AUC increase between the 150 and 250 mg dose levels (Supplementary Table 7). The elimination phase started at around 24 hours post-dose, with a geometric mean $t_{1/2}$ of between 23.4 and 38.7 hours for total KL1333 across all healthy participant cohorts. In subjects with PMD, the shapes of the plasma concentration versus time profiles were generally comparable to healthy participants (Supplementary Fig. 3 and 4). $t_{1/2}$

could not be calculated in PMD subjects due to the shorter duration of blood sample collection following the last dose. For 50 mg KL1333 QD, exposures in PMD subjects were lower when compared to the healthy participants on day 1 but were similar at day 10 (Supplementary Table 7).

Classification of Evidence

This study provides Class I evidence that KL1333 is safe and well tolerated when administered to healthy individuals as single oral doses of 25 mg with and without food, as QD doses of 25 to 75 mg, as TID doses of 50 mg for 10 days, and when administered to adult patients with genetically confirmed multisystemic PMDs as QD doses of 50 mg for 10 days.

References

1. Patgiri A, Skinner OS, Miyazaki Y, et al. An engineered enzyme that targets circulating lactate to alleviate intracellular NADH:NAD(+) imbalance. Nat Biotechnol 2020;38:309-313.
2. Pirinen E, Auranen M, Khan NA, et al. Niacin Cures Systemic NAD(+) Deficiency and Improves Muscle Performance in Adult-Onset Mitochondrial Myopathy. Cell Metab 2020;32:144.
3. Thompson Legault J, Strittmatter L, Tardif J, et al. A Metabolic Signature of Mitochondrial Dysfunction Revealed through a Monogenic Form of Leigh Syndrome. Cell Rep 2015;13:981-989.
4. Fujita Y, Ito M, Kojima T, Yatsuga S, Koga Y, Tanaka M. GDF15 is a novel biomarker to evaluate efficacy of pyruvate therapy for mitochondrial diseases. Mitochondrion 2015;20:34-42.
5. Sharma R, Reinstadler B, Engelstad K, et al. Circulating markers of NADH-reductive stress correlate with mitochondrial disease severity. J Clin Invest 2021;131.
6. Gores GJ, Flarsheim CE, Dawson TL, Nieminen AL, Herman B, Lemasters JJ. Swelling, reductive stress, and cell death during chemical hypoxia in hepatocytes. Am J Physiol 1989;257:C347-354.
7. Titov DV, Cracan V, Goodman RP, Peng J, Grabarek Z, Mootha VK. Complementation of mitochondrial electron transport chain by manipulation of the NAD+/NADH ratio. Science 2016;352:231-235.

8. McElroy GS, Reczek CR, Reyfman PA, Mithal DS, Horbinski CM, Chandel NS. NAD+ Regeneration Rescues Lifespan, but Not Ataxia, in a Mouse Model of Brain Mitochondrial Complex I Dysfunction. Cell Metab 2020;32:301-308 e306.

9. Jaiswal AK. Regulation of genes encoding NAD(P)H:quinone oxidoreductases. Free Radic Biol Med 2000;29:254-262.

10. Hwang JH, Kim DW, Jo EJ, et al. Pharmacological stimulation of NADH oxidation ameliorates obesity and related phenotypes in mice. Diabetes 2009;58:965-974.

11. Seo KS, Kim JH, Min KN, et al. KL1333, a Novel NAD(+) Modulator, Improves Energy Metabolism and Mitochondrial Dysfunction in MELAS Fibroblasts. Front Neurol 2018;9:552.

12. Lowry OH, Passonneau JV, Schulz DW, Rock MK. The measurement of pyridine nucleotides by enzymatic cycling. J Biol Chem 1961;236:2746-2755.

13. Matsumura H, Miyachi S. [43] Cycling assay for nicotinamide adenine dinucleotides. In: San Pietro A, ed. Methods in Enzymology: Academic Press, 1980: 465-470.

14. Schaefer AM, Phoenix C, Elson JL, McFarland R, Chinnery PF, Turnbull DM. Mitochondrial disease in adults: a scale to monitor progression and treatment. Neurology 2006;66:1932-1934.
